# Novel saccharin analogs as promising antibacterial and anticancer agents: synthesis, DFT, POM analysis, molecular docking, molecular dynamic simulations, and cell-based assay

**DOI:** 10.3389/fphar.2022.958379

**Published:** 2022-10-04

**Authors:** Magda H. Abdellattif, Ahmed Elkamhawy, Mohamed Hagar, Taibi Ben Hadda, Wesam S. Shehab, Wael Mansy, Amany Belal, M. M. H. Arief, Mostafa A. Hussien

**Affiliations:** ^1^ Department of Chemistry, College of Science, Taif University, Taif, Saudi Arabia; ^2^ College of Pharmacy, Dongguk University-Seoul, Goyang, South Korea; ^3^ Department of Pharmaceutical Organic Chemistry, Faculty of Pharmacy, Mansoura University, Mansoura, Egypt; ^4^ Chemistry Department, Faculty of Science, Alexandria University, Alexandria, Egypt; ^5^ Department of Pharmaceutical Chemistry, Faculty of Pharmacy, Umm Al-Qura University, Makkah, Saudi Arabia; ^6^ Laboratory of Applied Chemistry and Environment, Faculty of Sciences, Mohammed Premier University, Oujda, Morocco; ^7^ Department of Chemistry, Faculty of Science, Zagazig University, Zagazig, Egypt; ^8^ Department of Clinical Pharmacy, College of Pharmacy, King Saud University, Riyadh, Saudi Arabia; ^9^ Department of Pharmaceutical Chemistry, College of Pharmacy Taif University, Taif, Saudi Arabia; ^10^ Department of Chemistry, Faculty of Science, Benha University, Benha, Egypt; ^11^ Department of Chemistry, Faculty of Science, King Abdulaziz University, Jeddah, Saudi Arabia; ^12^ Department of Chemistry, Faculty of Science, Port Said University, Port Said, Egypt

**Keywords:** saccharinyl hydrazide, DFT, docking, POM analyses, pharmacophore sites, MD

## Abstract

Saccharine is a pharmacologically significant active scaffold for various biological activities, including antibacterial and anticancer activities. Herein, saccharinyl hydrazide (**1**) was synthesized and converted into 2-[(2Z)-2-(1,1-dioxo-1,2-dihydro-3H-1λ^6^,2- benzothiazole-3-ylidene) hydrazinyl] acetohydrazide (**5**), which was employed as a key precursor for synthesizing a novel series of small molecules bearing different moieties of monosaccharides, aldehydes, and anhydrides. Potent biological activities were found against ***Staphylococcus*** and ***Escherichia coli***, and the results indicated that compounds **6c** and **10a** were the most active analogs with an inhibition zone diameter of 30–35 mm ***.*** In cell-based anticancer assay over Ovcar-3 and M-14 cell lines, compound **10a** was the most potent analog with IC_50_ values of 7.64 ± 0.01 and 8.66 ± 0.01 µM, respectively. The Petra Orisis Molinspiration (POM) theoretical method was used to calculate the drug score of tested compounds and compare them with their experimental screening data. Theoretical DFT calculations were carried out in a gas phase in a set of B3LYP 6-311G (d,p). Molecular docking studies utilizing the MOE indicated the best binding mode with the highest energy interaction within the binding sites. The molecular docking for Ovcar-3 was carried out on the ovarian cancer protein (3W2S), while the molecular docking for M-14 melanoma was carried out on the melanoma cancer protein (2OPZ). The MD performed about 2ns simulations to validate selected compounds’ theoretical studies.

## 1 Introduction

Saccharin (1,2-benzo isothiazole-3-one-1, 1-dioxide) is a heterocyclic compound used as a sweetener in its sodium salt. Furthermore, it has been widely incorporated into a variety of biologically active compounds (Biswa et al., 2014), human mast cell tryptase inhibitors (Borah et al., 2021; Combrink et al., 1998), and anti-inflammatory (Dhole et al., 2013), and antioxidants (Hamama et al., 2011). Another advantage of saccharine was using it as a catalyst in synthesizing some dihydro-2-oxypyrrole derivatives (Mohamadpour et al., 2016). So, in connection (Huang et al., 2021, Abdel-Rahman et al., 2013, and Al Bayati et al., 2015; Magda et al., 2016) with our ongoing interest, the goal was to develop new synthetic strategies for constructing systems involving saccharin or its derivatives due to its significant biological and pharmacological activities. Furthermore, Schiff bases have been reported to exhibit a broad range of biological and pharmacological properties.

Schiff-base formation from the reaction of tryptophan and saccharin (Mahmood and AlJuboori, 2020).


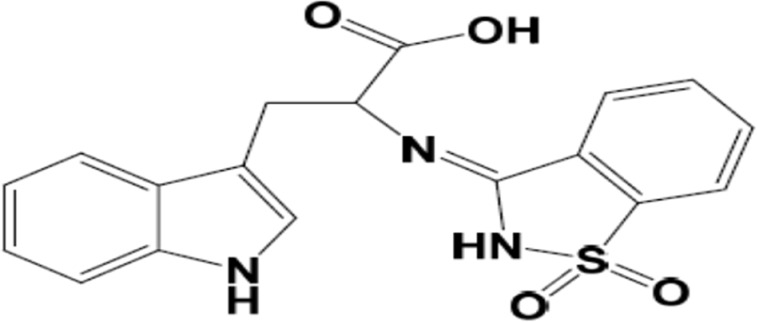


Structures of 1,2-benzisothiazole-3-one 1,1-dioxide (**1**, saccharin), 1*H*-tetrazole (**2**), and 1,3,4-1,3,4-thiadiazole (**3)** (Frija et al., 2019).


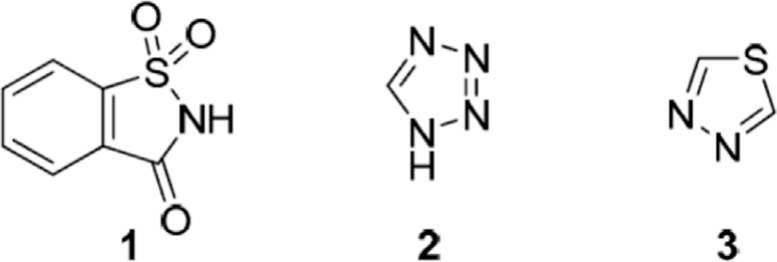


Due to the aforementioned facts, while we continue to pursue our interest in the attachment of carbohydrate residues, compound searching for potent leads as antimicrobial and anticancer agents continues (Abdellattif et al., 2020).

Similar compounds with non-blocker biological activities


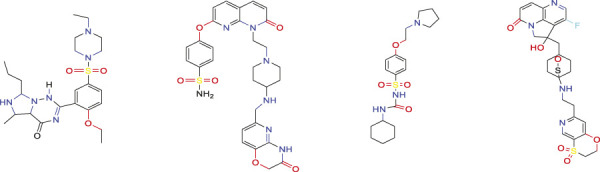


Molecular docking studies are essential in proving or predicting the biological activities obtained from experimental studies (Abdellattif et al., 2021). 2OPZ is an XIAP–BIR3 in complex with an AVPF peptide. The XIAP protein provides instructions for making a protein found in many cells, including immune cells. It helps to protect these cells from self-destructing (undergoing apoptosis) by blocking (inhibiting) the action of certain enzymes called caspases, which are necessary for apoptosis. In particular, the XIAP protein inhibits caspase enzymes 3, 7, and 9. The XIAP protein also plays a role in several other signaling pathways involved in various body functions (Holcik et al., 2001, Marsh et al., 2010, and Obexer and Ausserlechner, 2014). From the perspective of docetaxel-resistant ovarian cancer, many studies attempt to explore the therapeutic potential of many drugs and show a high degree of binding to the EGFR kinase “3W2S.” Expanding medicate affectability by focusing on EGFR receptors is one of the basic techniques in ovarian disease treatment (Sogabe et al., 2013; Sait et al., 2019).

Another method, theoretical DFT, and molecular docking studies were also used to predict and investigate the biological activities of the new promising synthesized compounds that were then proven (Malhotra et al., 2017 and Kumer et al., 2019). POM analysis is one of the well-known approaches to accessing synthetic drugs’ pharmacokinetic properties. It helps to identify and indicate the type of pharmacophore site that affects biological activity, wherever there may have been changes in the chemical substitution (Rachedi et al., 2019). Most of the synthesized compounds’ drug scores were an essential parameter for the compound possessing the drug properties (Kumar et al., 2018; Hagar et al., 2019). All the obtained results were validated by molecular dynamics studies (Joshi et al., 2018; Ali et al., 2018). The consequence of the present research strengthens the applicability of these compounds by encouraging anticancer and antibacterial drugs that could help medicinal chemists and pharmaceuticals further design and synthesize more effective drug candidates (Shawon et al., 2018 and Jayakumar and Anishetty, 2014).

## 2 Results and discussion

### 2.1 Synthesis of compounds 1–10

#### 2.1.1 Synthesis of compounds 1–4

**SCHEME 1:**
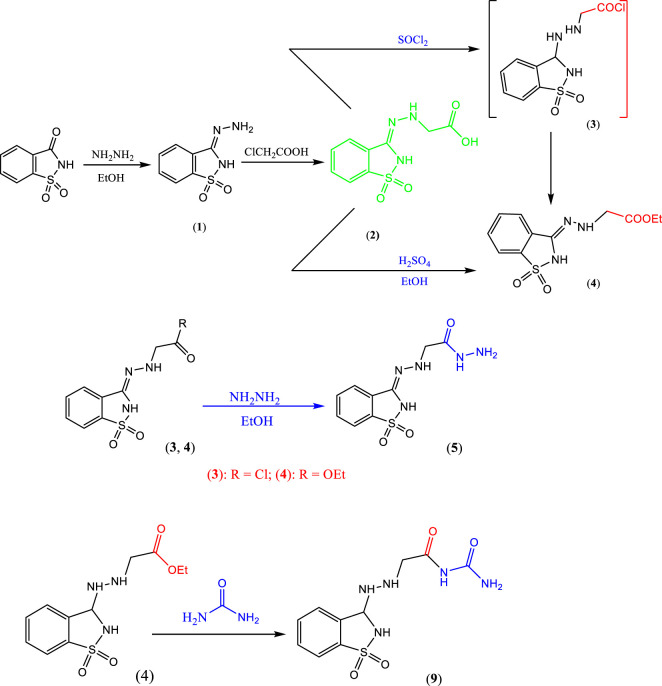
Synthesis of precursor compounds **1, 2, 3, 4, 5, and 9**.

The key starting material **1** (Abdellattif et al., 2020) was prepared by refluxing saccharin and hydrazine hydrate in ethanol with a few drops of glacial acetic acid (Scheme 1), where its structure was confirmed by correct elemental analysis, the IR spectrum, and showed broad bands at 3,323 cm^−1^ for NH, aromatic CH, 1,659 cm^−1^ for C=N, and 1,340 cm^−1^ for SO_2_.

When compound **1** was refluxed with chloroacetic acid in xylene, it afforded the hydrazinyl acetic acid derivative **2** (Scheme 1). The structure of compound **2** was confirmed from its correct elemental analysis, the IR spectrum, which showed the new functional group bands at 3,423 cm^−1^ broad for OH of acid and 1717 cm^−1^ for CO of acid. Mass spectrum of compound **2** showed a short molecular ion peak at m/z = 255 (12%) and the base peak at m/z = 155.

#### 2.1.2 Synthesis of compounds 3–5

##### 2.1.2.1 Synthesis of compound 5

The acetic acid hydrazide derivative **5** was synthesized via two routes, namely, hydrazinolysis of prepared acid chloride **3** (*in situ*) or ester **4** in refluxing ethanol (Scheme 1). The structure of compound **4** was established from its correct elemental analysis, and the IR spectrum displayed bands for the new -COOEt at 2,980 cm^−1^ for aliphatic CH and 1,720 cm^−1^ for CO. The structure of acetic acid hydrazide derivative **5** was inferred from its correct elemental analysis, and the IR spectrum displayed a band of the new functional at 3,441 cm^−1^ for NH_2_ and 3,167 cm^−1^ for NH. The mass spectrum showed a molecular ion peak at m/z = 269 (11%).

#### 2.1.3 Synthesis of compounds 6a–c

**SCHEME 2:**
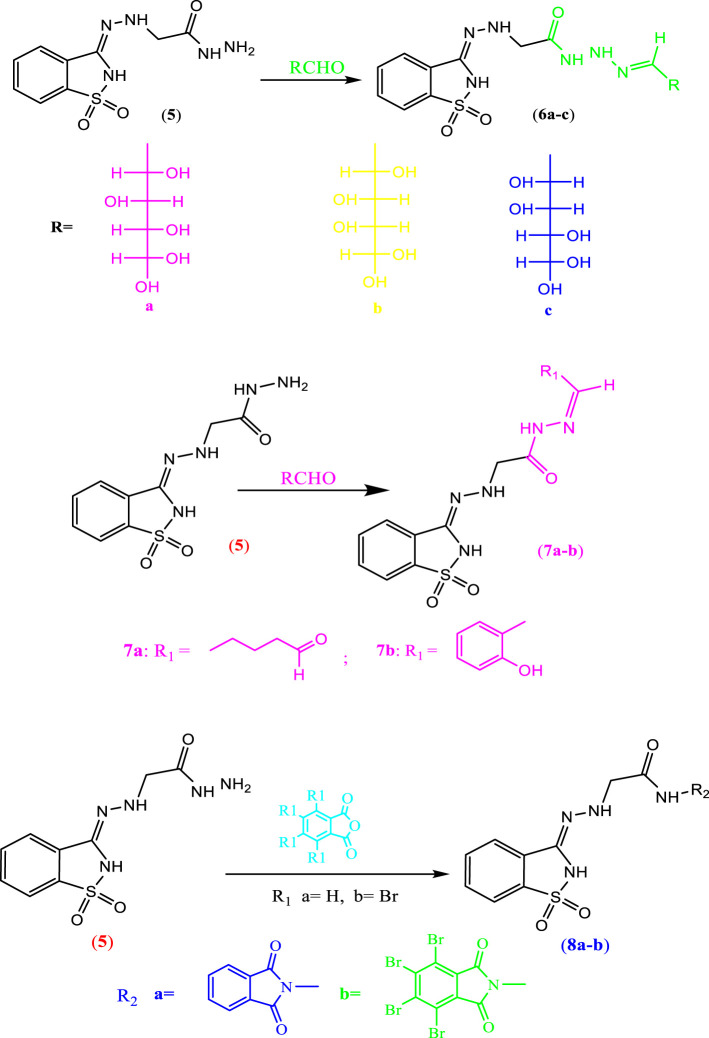
Synthesis of the precursor compounds **6a–c, 7a–b, and 8a–b**.

Most saccharin derivatives are important in medicinal chemistry (Huang et al., 2021; Abdel-Rahman et al., 2013 and Al Bayati et al., 2015). Furthermore, Schiff bases have a broad spectrum of biological activities, such as anti-inflammatory, anticonvulsant, and anti-HIV. So, when hydrazide **5** was treated with monosaccharides, namely, glucose, galactose, mannose, and xylose, the corresponding sugar hydrazone **6a–c** was produced in good yield (Scheme 2).

The structure of compounds **6a–c** was elucidated from their correct elemental analysis and spectral data, and the IR of compound **6a** showed bands at 3,379 cm^−1^ broad of OH groups and 3,224 cm^−1^ for aliphatic CH groups. The mass spectrum of the compound showed a molecular ion peak at m/z = 431 (0.1%).

The IR of compound **6b** showed bands at 3,400 cm^−1^ broad for OH groups and 2,972 cm^−1^ for aliphatic CH. The mass spectrum of compound **6b** showed a molecular ion peak at m/z = 431 (0.3%).

The IR of compound **6c** showed bands at 3,340 cm^−1^ and 3,234 cm^−1^ (broad) for OH and aliphatic CH groups, respectively. The mass spectrum of compound **6c** showed a molecular ion peak at m/z = 431 (1.5%).

#### 2.1.4 Synthesis of compounds 7a, b, and 8a, b

##### 2.1.4.1 Reaction of hydrazide derivative 5 with aldehyde

In this investigation, the hydrazide derivative **5** reacted with aldehyde, namely, glutaric dialdehyde or salicylaldehyde in ethanol, and with a few drops of acetic acid (glacial), it gave the corresponding Schiff bases **7a** and **7b,** respectively (Scheme 2). The structure of compounds **7a** and **7b** were confirmed from their correct elemental analysis, and the IR of compound **7a** showed bands at CH, 2,934 cm^−1^; 2,865 cm^−1^ for aliphatic CH, 1,688 cm^−1^; 1,629 cm^−1^ for CO. The IR of compound **7b** showed bands at 3,383 cm^−1^ for OH (phenolic) and 2,920 cm^−1^ for aliphatic CH. The mass spectrum of compound **7b** showed a molecular ion peak at m/z = 373 (0.1%) and a base peak at m/z = 240 (100%).

##### 2.1.4.2 Reaction of hydrazide derivative 5 with anhydride

Alternatively, hydrazide derivative **5** reacted with anhydride (phthalic anhydride and tetrabromo phthalic anhydride) by refluxing in ethanol and with a few drops of glacial acetic acid to **8a–8b**, respectively. The structure of compounds **8a** and **8b** was confirmed from their correct elemental analysis and spectroscopic data. The IR of compound **8a** displayed bands at 3,240 cm^−1^ for aliphatic CH, and 1,770 cm^−1^; 1727 cm^−1^; 1,750 cm^−1^ for CO and the IR of compound **8b** exhibited bands at 3,243 cm^−1^ for aliphatic CH, and 1,777 cm^−1^; 1,705 cm^−1^ for CO. The mass spectrum of compound **8b** did not give the molecular ion peak (as it proved very unstable), but it showed the following fragments at m/z = 462 (1%), 428 (1%), 253 (5%), 283 (5%), 210 (4%), 200 (2%), 183 (100%), and 140 (7.5%).

#### 2.1.5 Synthesis of compound 9

When the saccharine derivative was reacted with urea in refluxing absolute ethanol containing a few drops of glacial acetic acid, it provided the desired compound **9** (Scheme 1). The structure of compound **9** was established from its correct elemental analysis. The IR spectrum showed bands at 3,327 cm^−1^ for NH, 3,167 cm^−1^ for NH_2_, and 1,777 cm^−1,^ and 1,705 cm^−1^ for CO.

#### 2.1.6 Synthesis of compounds 10a, b

**SCHEME 3:**
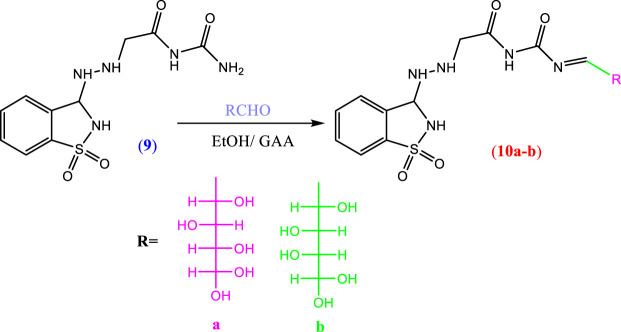
Synthesis of the precursor compound (**10a–b)**.

When compound **9** was refluxed with different monosaccharides, namely, glucose or galactose, in absolute ethanol containing a few drops of glacial acetic acid, it furnished the corresponding glycoside derivatives **10a, b (** Scheme 3).

The structure of compound **10a** was established from its correct elemental analysis. The IR spectrum showed bands at 3,406 cm^−1^ (broad) for OH. In addition, the structure of compound **10b** was confirmed from its correct elemental analysis. The IR spectrum showed bands at 3,459 cm^−1^ (broad) for OH additionally as new functional groups.

### 2.2 Modeling and theoretical studies

#### 2.2.1 DFT molecular geometry

**FIGURE 1:**
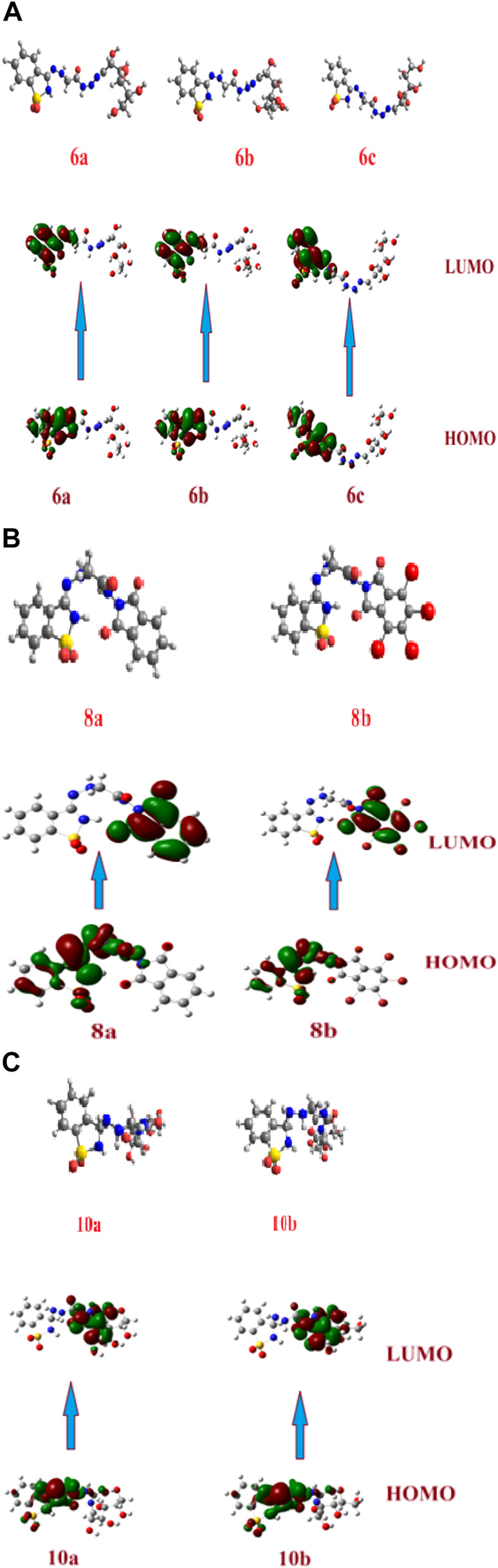
**(A)** Optimized geometrical structures of the prepared compounds (6a–c) and surface plots calculated from ground state isodensity for FMOs for the investigated compounds **6a–c**. **(B)**. Optimized geometrical structures of the prepared compounds (8a–b) and surface plots calculated from ground state isodensity for FMOs for the investigated compounds 8a–b. **(C)** Optimized geometrical structures of the prepared compounds (10a–b) and surface plots calculated from ground state isodensity for FMOs for the investigated compounds 10a–b.

Theoretical DFT stimulation was achieved in the gas phase using the B3LYP/6-311G (d,p) basis set introduced into Gaussian 9, which included predicting each prepared compound’s geometrical optimization (**6a–c, 8a, b, and 10 a, b**) to determine the molecular structure of the lowest energy. Afterward, it was time to calculate the vibrational frequencies at the optimum geometrical structure, during which several thermochemical parameters were obtained. Due to the absence of imaginary frequency, all optimized geometrical structures of the prepared compounds are stable. The calculations were carried out for the prepared compounds. It entailed performing a geometrical optimization on each compound to evaluate the minimum energy structure, calculating the frequency at the optimized geometry, and computing various thermochemical quantities. The results of the theoretical calculations of DFT showed that none of the compounds are planar, as shown in Figures 1A–C.

Many chemical reactivity modes have been illustrated using the frontier orbitals; the highest occupied molecular orbital (HOMO) and lowest unoccupied molecular orbital (LUMO). The frontier orbitals have recently depicted the biological reactivity of newly discovered compounds, with their energy gab factored in. In addition, there is a connection between the energy level and the energy gap between the frontier orbitals and a variety of biological activities, such as antifungal (Malhotra et al., 2017; Kumer et al., 2019; Kumar et al., 2018; Hagar et al., 2019; and Joshi et al., 2018), anticancer (Ali et al., 2018; Rachedi et al., 2019; and Shawon et al., 2018), antimicrobial (Jayakumar and Anishetty, 2014; Wist et al., 2007; and Abdellattif et al., 2020), and cytotoxic (da Costa et al., 2018) activities and a new drug design field that could be investigated.

Several important factors, such as the potential need for electronic ionization of HOMO electrons (I = -EHOMO) and the electron affinity calculated from the LUMO energy level (A = -ELUMO), could be predicted from the energy level and the energy gap of the frontier molecular orbitals (E_HOMO_, E_LUMO_, and ΔE). Softness (*δ*), global hardness (*η*), electronegativity (χ), and electrophilicity (*ω*) are only a few of the chemical reactivity descriptors that FMO can evaluate. These variables could be determined using the formulas as follows (Lewis 2003):

$$\chi =-\frac{1}{2}\left(E_{HOMO}+E_{LUMO}\right),$$ (1)

$$\eta =-\frac{1}{2}\left(E_{HOMO}-E_{LUMO}\right),$$ (2)

$$\delta =\frac{1}{\eta },$$ (3)

$$\omega =\frac{\chi^{2}}{2\eta }.$$ (4)

The electronegativity (χ) value measures the ability of a compound to accept electrons, also known as Lewis acidity. Global hardness (η) can predict the degree to which molecules are prohibited from transferring charge; however, global softness (δ) measures electronic transition capability.

The electrophilicity (*ω*) is an assessment of the lower energy for the electronic transition due to the large distance between the acceptor LUMO and the donor HOMO, which can be easily determined using the calculated electronegativity and chemical hardness.

The DFT evaluation intensifies for the wilderness atomic orbitals, as far as the energy level of HOMO and LUMO, just as with their energy gap of the examined ones (**6a–c, 8a, b, and 10a, b**) was contemplated, Figures 1A–C. Recently, it has been noted that the energy level and the energy gap of the frontier molecular orbitals (HOMO and LUMO) could impact the limiting proclivity of mixtures and how it administers the interaction with receptor proteins. The levels of HOMOs for the prepared compounds 6a–c are in the range of −5.82 to −6.05 eV, and the energy level of the LUMO is in the range of −1.83 eV to −2.00 eV. Since there is no change in the degree of conjugation and the electronic nature of the attached polar groups to affect the difference in the energy level of the compounds under investigation, the energy difference is almost the same at 4.00 eV. Based on the energy level of the investigated compounds’ HOMOs, compound 6b is expected to have the highest lying HOMO (−6.05 e.V.) and is the most vulnerable for its ability to be an electron donor.

On the contrary, 6c has been determined as the best electron acceptor E_LUMO_ = −1.83 eV. The high level of HOMO could allow a reasonable degree of electron transfer from the compound to the receptor, and therefore; this would explain the highest value of binding affinity (binding energy = −6.63) of 6b relative to the other analogous compounds. On the other hand, the energy gaps of the FMOs of the diimide compounds 8a and 8b are 2.81 and 3.26 eV, respectively, showing clearly that the compounds 8a in comparison with 8b are the least of the energy differences. Furthermore, it could illustrate the high binding affinity in molecular coupling scores (binding energy = −6.23 and −6.53 for 8a and 8b, respectively). Compounds 10a and b have almost the same energy gap between the FMOs. This is because the attached groups have neither conjugation nor polarity Table 1.

**TABLE 1:** Chemical reactivity descriptors and dipole moment (µ, Debye) of investigated compounds (**6a–c, 8a, b, and 10a, b**).

Compound	6a	6b	6c	8a	8b	10a	10b
HOMO	−5.91	−6.05	−5.82	−6.08	−6.22	−6.69	−6.76
LUMO	−1.89	−2.00	−1.83	−2.81	−3.31	−1.80	−1.97
**ΔE**	4.02	4.05	3.99	3.26	2.91	4.90	4.78
Χ	3.90	4.03	3.83	4.45	4.77	4.25	4.37
Η	2.01	2.03	2.00	1.63	1.46	2.45	2.39
Δ	0.50	0.49	0.50	0.61	0.69	0.41	0.42
Ω	3.78	4.00	3.67	6.06	7.80	3.68	3.99
A	1.89	2.00	1.83	2.81	3.31	1.80	1.97
I	5.91	6.05	5.82	6.08	6.22	6.69	6.76
µ	4.87	5.87	6.87	8.87	9.87	10.87	11.87

The results of the molecular docking score and the energies of all investigated compounds could reveal that compound **6b** is expected to possess the best binding affinity score, which could be illustrated from its chemical descriptor values of electronegativity (χ = 4.03), global hardness (η = 2.03), global softness (δ = 0.49), and its electrophilicity (ω = 4.00). Therefore, these parameters could be shared to explain the appraised molecular docking results.

### 2.3 Molecular electrostatic potential

The molecular electrostatic potential (MEP) is an important parameter to predict, as it validates evidence related to the reactivity of the compounds under investigation as enzyme inhibitors. Furthermore, because the MEP represents the molecular size and form of the positive, negative, and neutral electrostatic potentials, it can predict the relationships of physicochemical properties with the molecular structure. Furthermore, the molecular electrostatic potential can be used to predict the sensitivity of the substances under investigation to electrophiles and nucleophiles.

**FIGURE 2:**
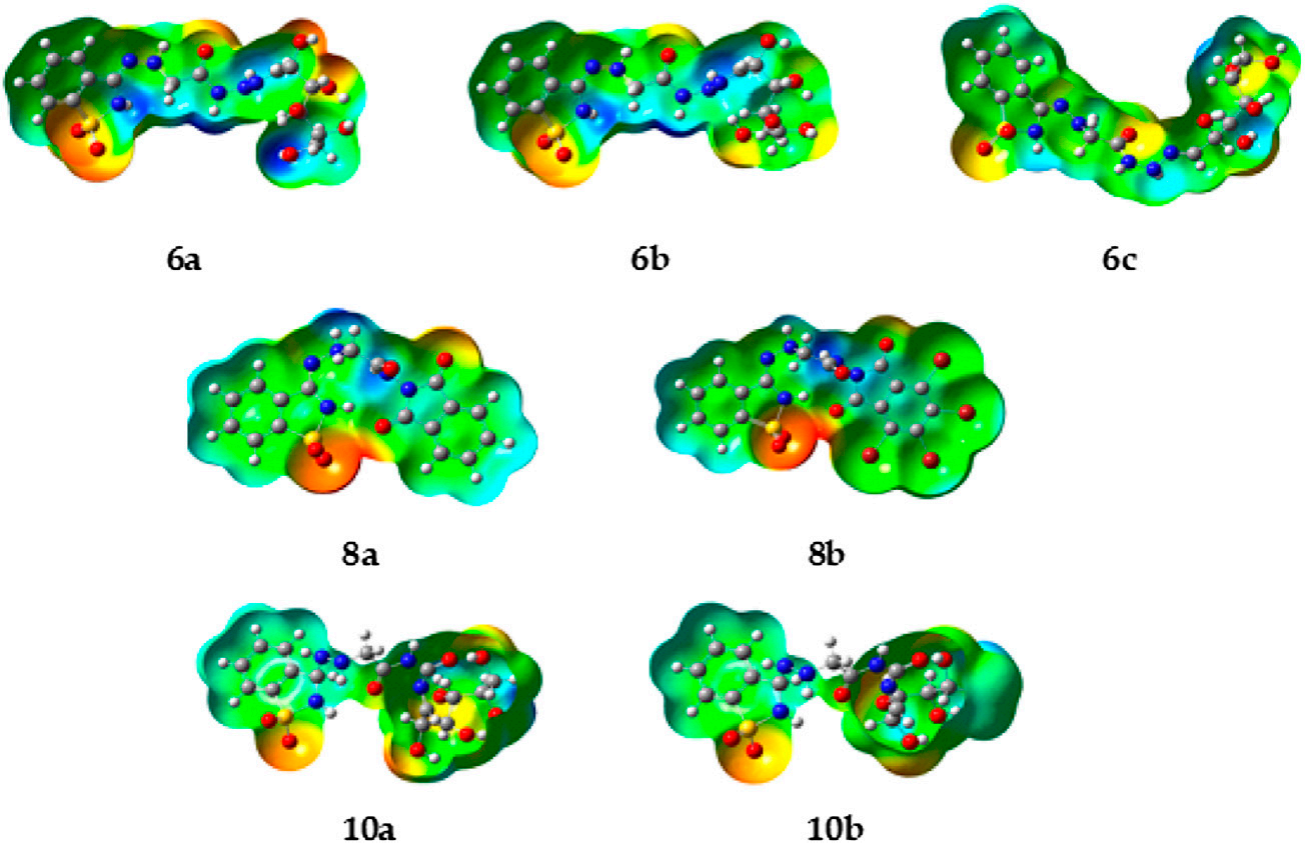
Molecular electrostatic potentials (MEP) of the investigated compounds **6a–c, 8a, b, and 10a–b**.

Figure 2 shows the molecular electrostatic potential determined using the same procedure for the same basis sets—the red color for oxygen, the blue color for nitrogen, and the yellow color for sulfur. The higher negative region of the MEP is the preferred site for an electrophilic attack, as indicated by the red color. As a result, an attacking electrophile will be attracted to negatively charged sites, while the red regions will be attracted to positively charged sites. The compound’s electronic nature and the attached groups’ electronegativity influence the molecular size and shape and the orientation of the negative, positive, and neutral electrostatic potentials. Therefore, the degree of binding affinity of the studied compounds to active site receptors might be attributed to differences in mapping the electrostatic potential around the compounds.

### 2.4 Molecular docking result

**FIGURE 3:**
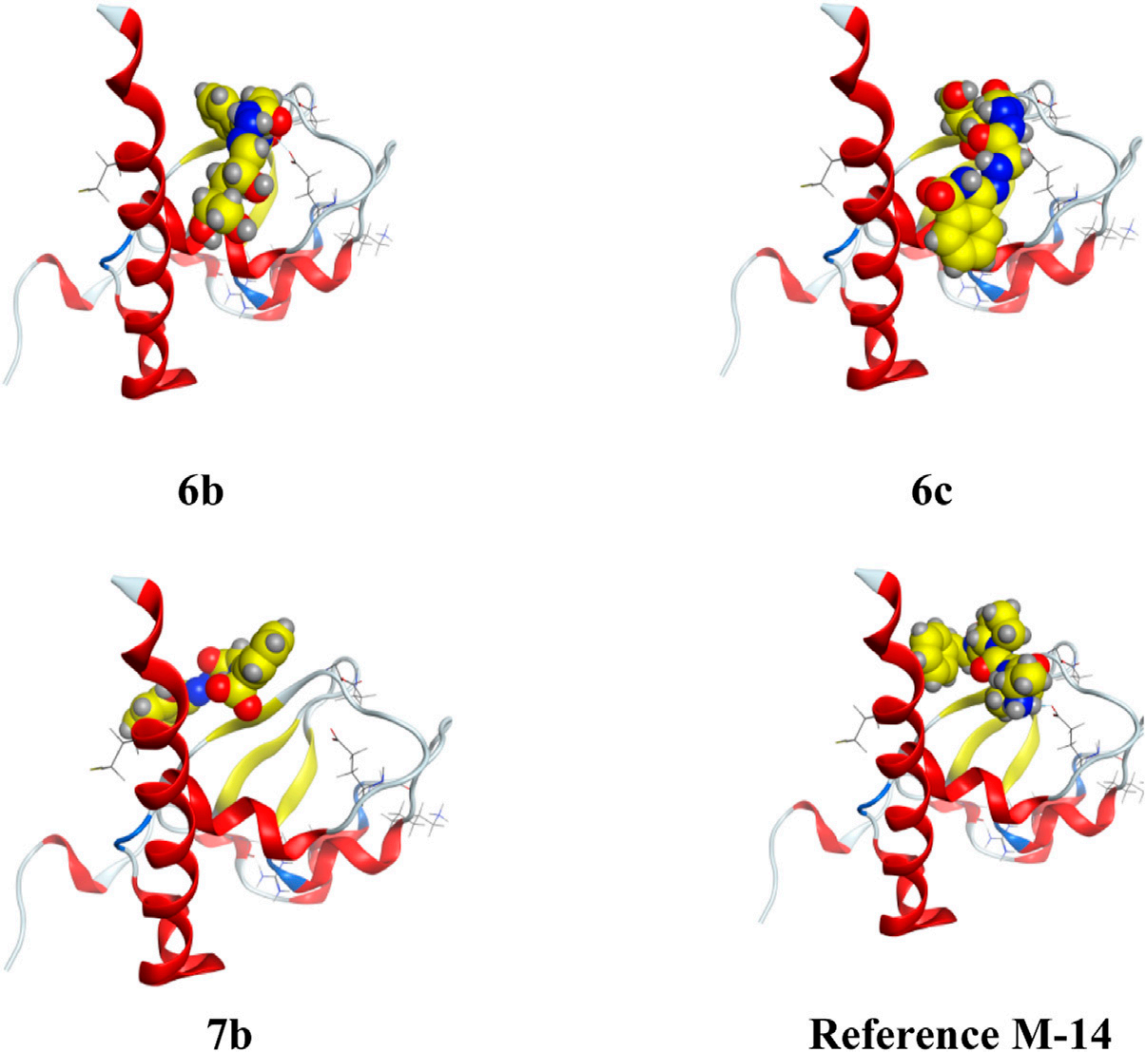
3D interaction of higher, lower, and reference compounds with 2OPZ’ melanoma cancer protein."

**FIGURE 4:**
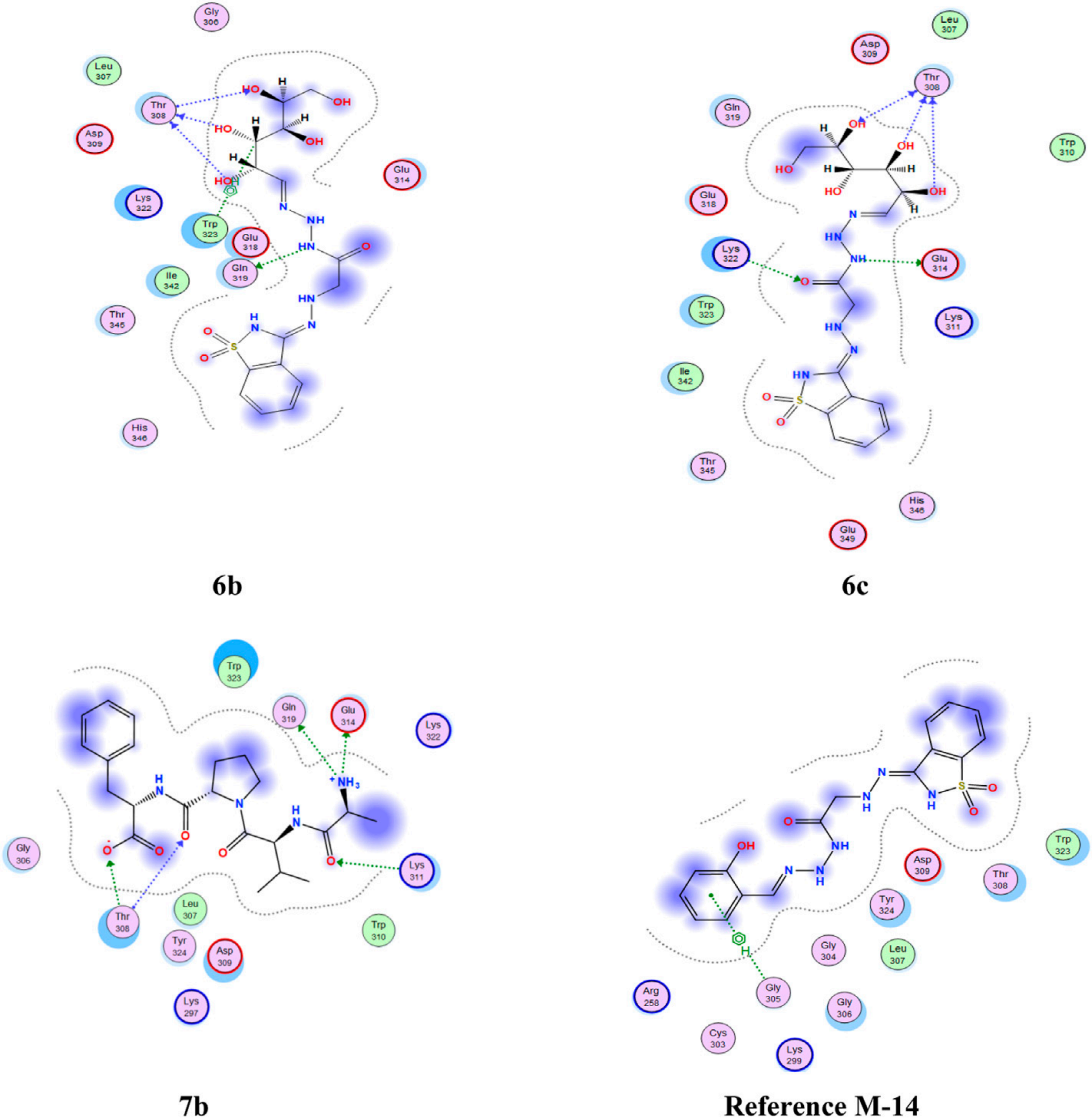
2D interaction of higher, lower, and reference compounds with 2OPZ’ melanoma cancer protein.'

Apoptosis inhibitor protein **2OPZ**, related to melanoma cancer, and 3W2S, EGFR kinase transferase protein, correlated to the inhibition of ovarian cancer, whereby the results obtained of the docking score energy are illustrated in Supplementary Materials Tables 2, 3, respectively. From the results obtained, **2OPZ** (Table 2) and Figures 3, 4, compounds (**6a, 6b, 6c, 6d, 7a, 7b, 8a, 8b, 9, 10a, 10b, and M-14 reference**) showed energy scores of **-6.39, -6.63, -6.56, -6.19, - 6.13, -5.72, -6.53, -5.92, -6.52, -6.25, and -6.54** kcal/mol**,** respectively. Compounds **6b**, **6c,** and **10a** showed a higher docking score energy of **-6.63**, **-6.56,** and **-6.54** kcal/mol**,** respectively**; 6 b** and **6c** have higher values than the reference compound, while compound **10a** has the same docking score value as the reference compound**,** while compound **7b** showed the lowest docking score energy of **−5.72** kcal/mol (Hosny et al., 2020).

**TABLE 2:** Docking score and energies of All compounds with 2OPZ “melanoma cancer protein.”

Comp.	S	rmsd_refine	E_conf	E_place	E_score1	E_refine	E_score2
6a	−6.39	1.52	59.05	−83.99	−8.17	−38.73	−6.39
	−6.28	2.71	65.74	−43.91	−8.11	−38.10	−6.28
	−6.20	2.73	51.53	−55.62	−9.50	−36.48	−6.20
	−6.18	1.41	56.78	−104.26	−9.00	−37.76	−6.18
	−6.04	1.99	55.93	−100.07	−10.70	−43.52	−6.04
6b	−6.63	2.19	60.83	−116.61	−10.28	−41.35	−6.63
	−6.31	1.56	68.83	−96.56	−10.28	−36.58	−6.31
	−6.29	2.52	61.00	−65.80	−9.08	−36.97	−6.29
	−6.27	1.34	62.32	−94.36	−10.38	−36.06	−6.27
	−6.24	1.17	57.98	−84.23	−8.90	−32.41	−6.24
6c	−6.56	3.19	65.79	−40.22	−9.10	−43.51	−6.56
	−6.49	2.67	72.07	−70.33	−9.62	−42.17	−6.49
	−6.47	1.68	65.87	−70.70	−9.54	−34.73	−6.47
	−6.46	1.69	62.25	−77.62	−11.69	−38.21	−6.46
	−6.43	2.81	56.45	−82.94	−10.76	−40.78	−6.43
7a	−6.13	1.95	−7.89	−76.20	−8.69	−38.46	−6.87
	−6.05	2.20	−10.49	−59.39	−8.85	−33.95	−6.55
	−6.00	2.67	−13.73	−41.79	−8.57	−35.55	−6.13
	−5.89	1.83	−8.92	−60.21	−8.72	−27.18	−5.89
	−5.80	2.46	−9.76	−80.07	−10.01	−29.61	−5.80
7b	−5.72	1.38	−6.60	−64.32	−8.50	−29.12	−5.72
	−5.40	1.00	32.61	−85.97	−9.77	−35.83	−5.40
	−5.38	3.74	−10.80	−49.87	−8.98	−32.24	−5.38
	−5.29	2.92	−12.16	−75.18	−9.36	−34.68	−5.29
	−5.21	4.04	−7.22	−63.95	−10.01	−12.83	−5.21
8a	−6.23	1.57	9.53	−80.26	−9.68	−34.78	−6.59
	−6.17	1.01	10.44	−86.78	−8.88	−25.53	−6.17
	−5.83	2.18	11.55	−65.22	−9.05	−27.61	−5.83
	−5.83	1.78	10.89	−38.06	−8.28	−37.31	−5.83
	−5.74	1.68	11.31	−90.05	−8.66	−31.07	−5.74
8b	−6.53	2.19	−14.57	−46.82	−8.31	−35.80	−6.33
	−6.10	2.50	−16.56	−36.81	−8.15	−30.05	−6.10
	−5.88	3.16	−10.84	−57.46	−8.44	−31.03	−5.88
	−5.74	4.05	−13.24	−59.81	−8.71	−34.15	−5.74
	−5.47	1.93	−11.54	−67.98	−8.72	−27.81	−5.47
9	−5.92	2.70	−130.55	−52.71	−8.99	−29.33	−5.92
	−5.29	2.13	−129.88	−64.07	−10.01	−22.17	−5.29
	−5.20	1.27	−128.78	−64.66	−10.70	−27.92	−5.20
	−5.14	2.20	−132.02	−47.16	−10.10	−27.35	−5.14
	−5.12	1.37	−127.98	−91.95	−9.98	−24.20	−5.12
10a	−6.52	1.59	−21.07	−41.63	−10.67	−42.94	−6.52
	−6.20	2.61	−23.51	−59.09	−9.68	−38.74	−6.20
	−6.19	2.78	−20.34	−83.19	−11.89	−29.88	−6.19
	−6.10	3.53	−20.55	−47.11	−10.45	−41.62	−6.10
	−5.97	1.48	−11.02	−63.04	−9.78	−36.61	−5.97
10b	−6.25	3.31	−19.32	−87.94	−11.01	−34.21	−6.65
	−6.02	2.39	−14.74	−60.21	−10.42	−32.39	−6.52
	−5.86	2.27	−15.20	−72.10	−10.03	−34.67	−6.46
	−5.14	2.08	−7.18	−64.24	−9.63	−41.82	−6.14
	−4.94	2.64	−3.89	−94.78	−9.20	−38.16	−5.94
ReffM14	−6.54	1.11	−127.91	−58.53	−12.37	−31.39	−6.54
	−6.23	1.93	−144.79	−53.07	−9.52	−36.77	−6.23
	−5.94	2.88	−139.51	−49.42	−11.84	−35.91	−5.94
	−5.80	2.15	−134.15	−33.52	−9.54	−30.17	−5.80
	−5.73	1.87	−142.99	−41.81	−10.12	−29.95	−5.73

**TABLE 3:** Docking score and energies of All compounds with 3W2S “ovarian cancer protein.”

Comp.	S	rmsd_refine	E_conf	E_place	E_score1	E_refine	E_score2
6a	−8.33	2.29	69.93	−106.20	−10.63	−46.21	−8.33
	−8.30	2.74	65.61	−84.18	−11.13	−32.80	−8.30
	−8.28	1.45	64.14	−113.74	−10.83	−56.80	−8.28
	−7.62	1.79	59.69	−108.12	−12.01	−49.02	−7.62
	−7.45	2.08	52.81	−87.19	−10.43	−30.52	−7.45
6b	−8.53	2.13	54.38	−109.65	−11.52	−41.34	−8.53
	−8.52	2.15	61.67	−76.10	−12.17	−42.97	−8.52
	−7.62	1.74	71.21	−75.57	−10.76	−40.36	−7.62
	−7.62	1.91	72.21	−75.55	−11.90	−43.03	−7.62
	−7.45	1.59	58.89	−109.72	−12.31	−44.09	−7.45
6c	−8.74	2.46	64.93	−81.59	−10.56	−50.73	−8.74
	−8.39	1.73	61.70	−113.67	−11.37	−42.97	−8.39
	−7.88	2.24	62.18	−81.19	−10.92	−54.02	−7.88
	−7.53	1.83	61.09	−79.47	−11.64	−40.09	−7.53
	−7.48	1.51	66.99	−104.27	−10.98	−44.67	−7.48
7a	−7.74	2.83	−4.13	−79.46	−10.63	−42.94	−7.74
	−7.69	1.09	−1.08	−96.91	−11.13	−34.58	−7.69
	−7.52	1.52	−4.33	−85.13	−11.59	−36.88	−7.52
	−7.48	2.14	−3.16	−92.38	−10.38	−45.14	−7.48
	−7.28	1.68	−11.67	−59.49	−10.47	−20.93	−7.28
7b	−8.32	1.62	6.01	−95.93	−11.07	−28.31	−8.32
	−8.01	2.05	−1.33	−104.84	−10.37	−40.71	−8.01
	−8.01	2.10	−9.30	−63.41	−10.27	−36.57	−8.01
	−7.64	1.42	−7.47	−99.10	−10.66	−46.18	−7.64
	−7.61	2.62	−5.54	−57.14	−11.59	−47.84	−7.61
8a	−8.32	2.17	24.76	−77.52	−11.16	−29.78	−8.32
	−7.44	1.21	14.24	−86.42	−10.32	−38.59	−7.44
	−7.08	1.38	13.31	−77.50	−10.58	−38.95	−7.08
	−6.80	1.22	11.00	−90.67	−10.26	−29.44	−6.80
	−6.65	2.01	25.52	−86.16	−9.92	−35.79	−6.65
8b	−8.48	1.96	−1.49	−52.09	−10.63	−43.90	−8.48
	−8.21	2.25	−9.18	−57.66	−11.57	−33.16	−8.21
	−8.19	1.05	−7.87	−68.31	−14.22	−31.24	−8.19
	−8.11	1.84	−16.45	−64.79	−10.78	−47.65	−8.11
	−8.10	3.20	−17.58	−75.30	−11.36	−52.21	−8.10
9	−6.79	1.78	−128.88	−60.51	−10.71	−34.18	−6.79
	−6.64	1.50	−126.93	−65.84	−10.69	−30.43	−6.64
	−6.09	2.10	−124.53	−69.95	−12.55	−27.91	−6.09
	−5.58	2.24	−124.52	−90.02	−10.64	−22.42	−5.58
	−5.58	1.10	−116.04	−66.13	−11.48	−7.77	−5.58
10a	−8.79	1.49	−21.29	−107.34	−11.46	−39.97	−8.79
	−8.40	3.00	−9.93	−57.77	−11.68	−43.62	−8.40
	−7.98	1.86	−2.26	−70.75	−11.79	−44.35	−7.98
	−7.93	1.97	−17.41	−102.28	−13.76	−37.46	−7.93
	−7.58	1.67	−3.91	−67.15	−13.24	−36.12	−7.58
10b	−8.67	1.61	2.12	−83.65	−11.38	−24.41	−8.67
	−8.49	1.68	−17.51	−114.11	−11.41	−45.70	−8.49
	−8.35	1.60	−5.46	−90.03	−11.45	−33.05	−8.35
	−7.84	2.34	−22.34	−74.19	−11.20	−20.77	−7.84
	−7.80	1.82	−13.38	−58.23	−11.55	−40.04	−7.80
Reffovarian	−11.07	3.00	−114.46	−22.82	−10.33	−71.66	−11.07
	−9.36	1.37	−98.31	−81.93	−10.96	−51.51	−9.36
	−8.78	2.38	−96.90	−57.56	−9.35	−47.68	−8.78
	−8.61	2.12	−96.05	−60.56	−10.19	−46.62	−8.61
	−8.54	1.52	−109.43	−130.36	−10.04	−44.69	−8.54

**FIGURE 5:**
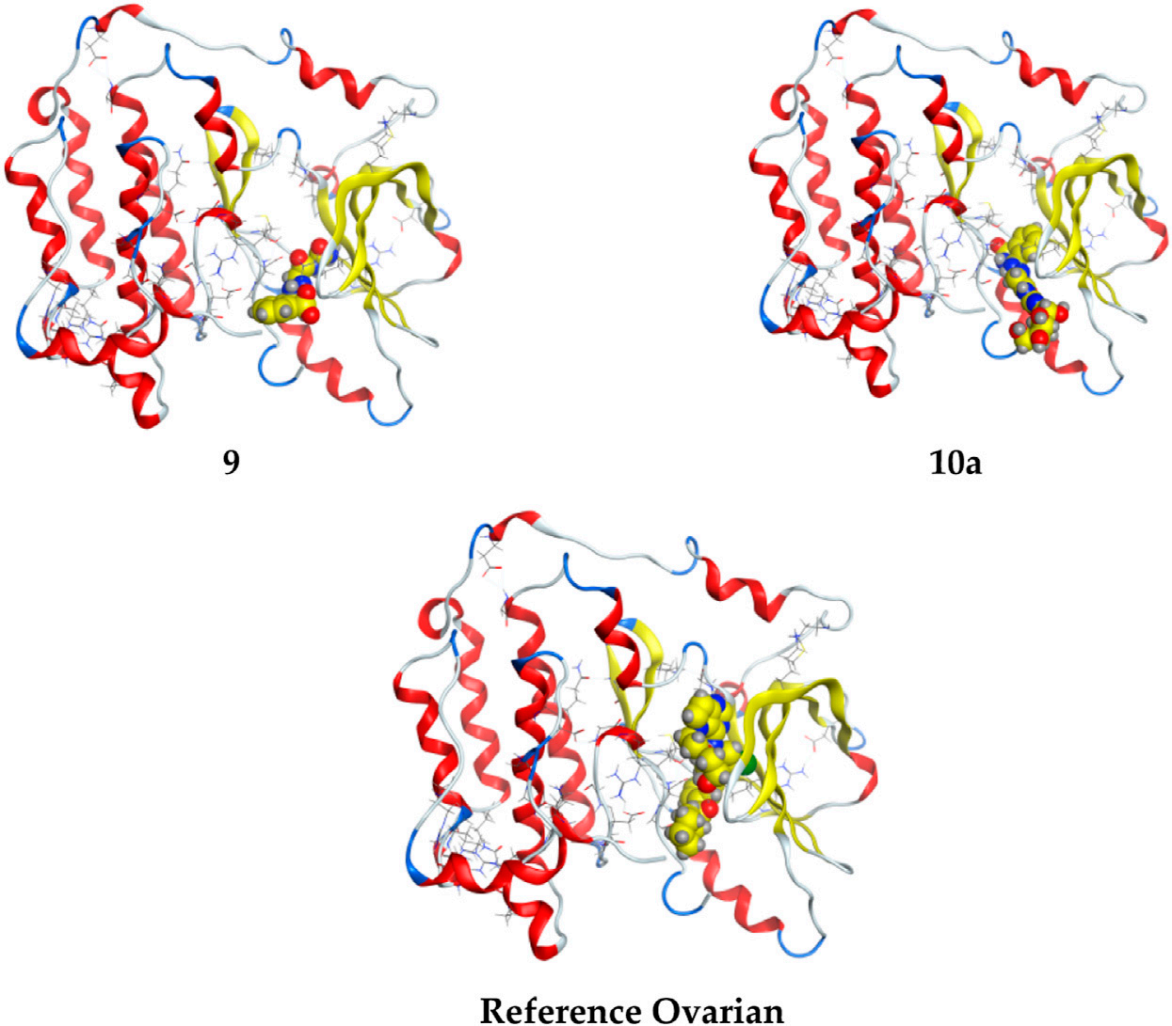
3D interaction of higher, lower, and reference compounds with the 3W2S’ ovarian cancer protein'.

**FIGURE 6:**
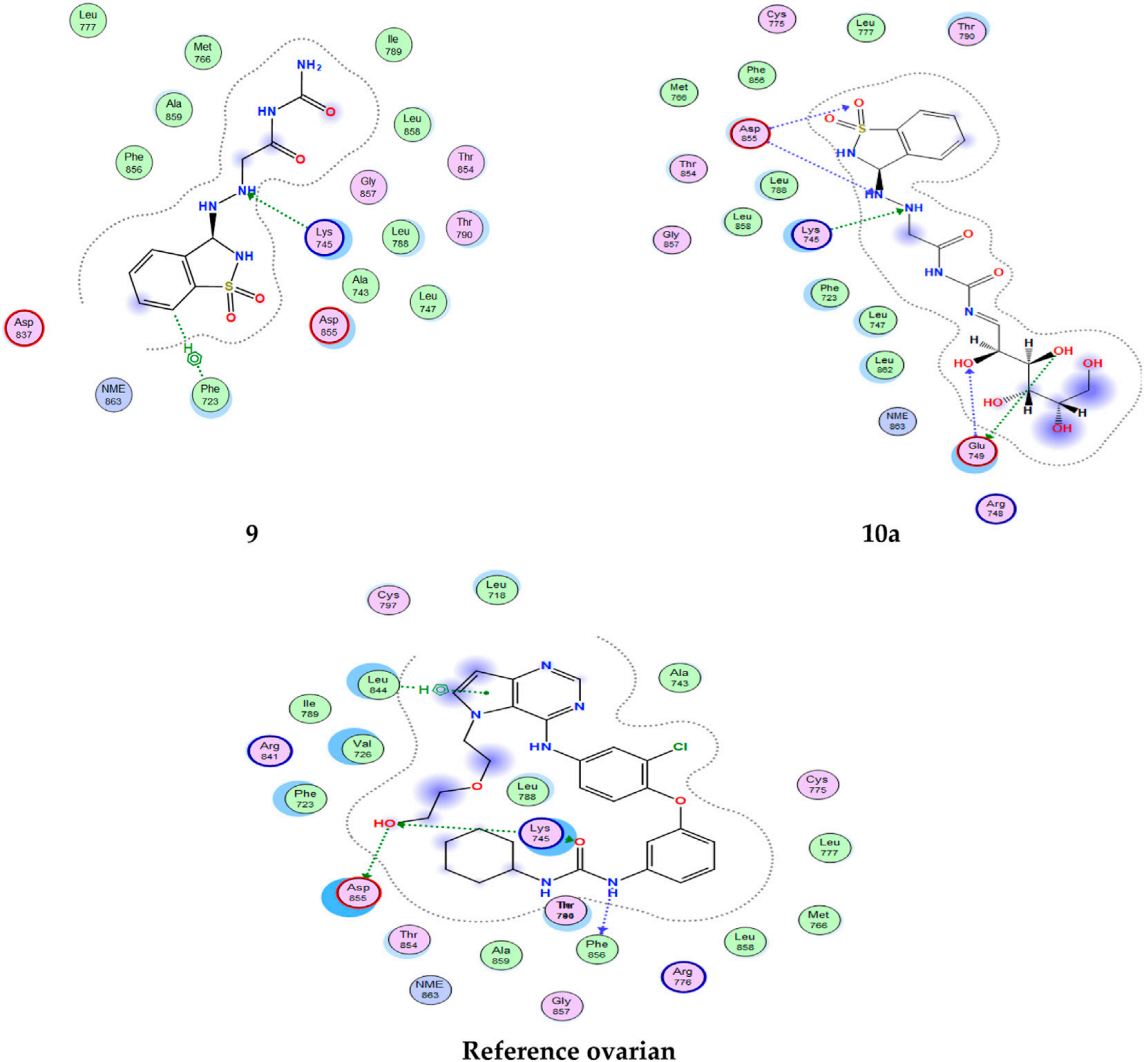
2D interaction of all compounds with 3W2S’ ovarian cancer protein.'

From the results obtained from **3W2S,** EGFR kinase transferase protein (Table 3), and Figures 5, 6 unfortunately, no compounds showed higher activity than the reference compound **−11.07**; however, they have shown promising activity. For example, the higher compound docking energy score was for compound **10a** at **−**8.79 kcal/mol, while the lowest was for compound 9 with (**−6.79** kcal/mol**)** [Aislyn et al., 2007].

**FIGURE 7:**
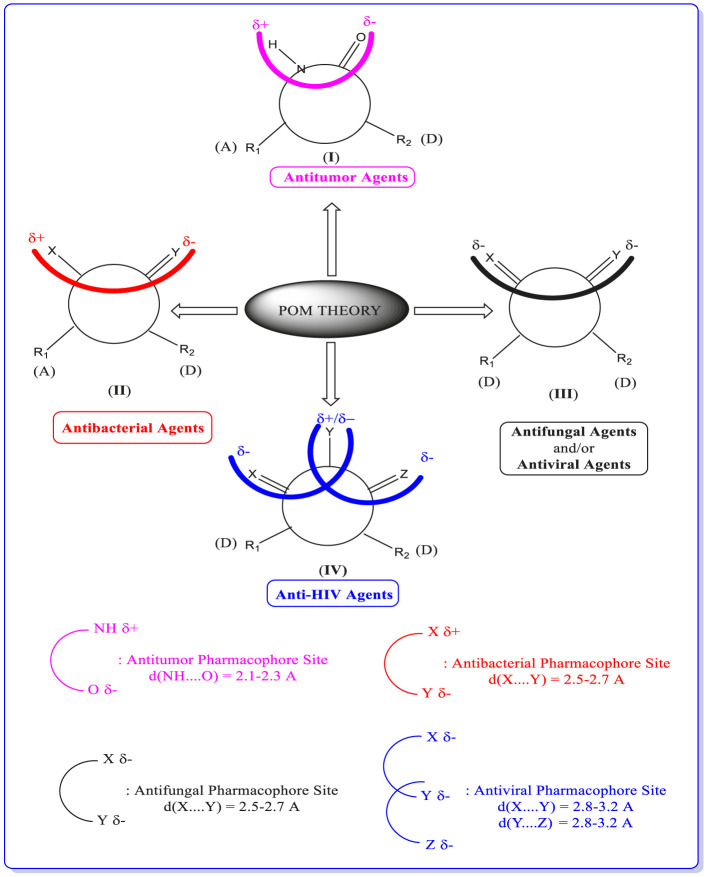
Concept and applications of POM theory in identifying and optimizing pharmacophore sites of various classes of drugs*,* developed by Prof. T. Ben Hadda (principal inventor of POM theory) in collaboration with NCI and TAACF of the United States (https://www.maroc.ma/fr/actualites/research-excellence-awards-une-vingtaine-de-travaux-de-recherche-et-dinnovation-primes).

All interactions of all compounds are listed in full in Figure 6 for 3D interactions and Figure 7 for 2D interactions in Supplementary Materials.

### 2.5 POM analyses and identification of pharmacophore sites

Synthesized compounds were also selected for the *in silico* POM study to calculate various general properties and predict the drug score for various bioactivities. Data were analyzed and compared with standard antibacterial and antitumor drugs. Osiris and Molinspiration are cheminformatics-based software tools that help to predict any possible side effects (Table 4) offered in the Supplementary Materials and calculate molecular properties, along with forecasting the compounds’ bioactivity scores in (Supplementary Tables 6) and found in the Supplementary Materials. The molecular inspiration data in Table 5 show that the synthesized compounds that satisfy the Lipinski rule behave like a drug and have modest protease and low enzyme inhibition properties. As 2/9 of the compounds tested have a molecular weight of less than 450, it can be highly absorbed since most traded drugs, that is, approximately 80% or more, have molecular weights in the same range. Compound **8b** with a molecular weight of 714.97 also shows a good drug similarity of 3.36, but its drug score is unacceptable for further consideration (DS = 9%).

**TABLE 4:** Docking interaction of all compounds with 3W2S “ovarian cancer protein.”

Compound	Ligand	Receptor	Interaction	Distance E	(kcal/mol)
6a	N 12	OD1 ASP 855 (A)	H-donor	2.98	−1.9
	O 38	O ASP 855 (A)	H-donor	2.93	−1.0
	N 48	OD1 ASP 855 (A)	H-donor	3.42	−1.8
	O 34	NZ LYS 745 (A)	H-acceptor	3.12	−0.7
	6-ring	CG1 VAL 726 (A)	pi-H	4.23	−0.7
6b	O 26	O PHE 856 (A)	H-donor	2.88	−0.7
	O 31	NZ LYS 745 (A)	H-acceptor	3.15	−1.4
	C 41	6-ring PHE 723 (A)	H-pi	4.13	−0.7
6c	O 27	OD1 ASP 837 (A)	H-donor	2.72	−1.9
7a	O 42	OD1 ASP 837 (A)	H-donor	2.75	−5.2
7b	O 17	CE LYS 745 (A)	H-acceptor	3.09	−1.6
	O 19	N PHE 856 (A)	H-acceptor	3.27	−0.6
8a	O 26	NZ LYS 745 (A)	H-acceptor	2.94	−0.5
	C 5	6-ring PHE 723 (A)	H-pi	3.44	−0.6
8b	N 12	O PHE 856 (A)	H-donor	2.85	−5.2
	N 18	O PHE 856 (A)	H-donor	2.94	−1.4
	BR 37	OD1 ASP 837 (A)	H-donor	3.37	−0.5
	O 26	NZ LYS 745 (A)	H-acceptor	3.01	−2.7
9	N 20	NZ LYS 745 (A)	H-acceptor	3.30	−3.7
	C 5	6-ring PHE 723 (A)	H-pi	3.60	−1.2
10a	O 40	OE1 GLU 749 (A)	H-donor	3.03	−1.8
	N 16	CA ASP 855 (A)	H-acceptor	3.37	−0.6
	O 18	N ASP 855 (A)	H-acceptor	3.30	−0.6
	N 20	NZ LYS 745 (A)	H-acceptor	3.48	−3.1
	O 35	N GLU 749 (A)	H-acceptor	3.33	−0.6
10b	O 35	O PHE 856 (A)	H-donor	3.01	−0.8
	O 18	NZ LYS 745 (A)	H-acceptor	3.03	−1.6
	O 31	N PHE 856 (A)	H-acceptor	3.27	−0.9
	O 40	NZ LYS 745 (A)	H-acceptor	3.08	−2.6
Reff. Ovarian	N 34	O PHE 856 (A)	H-donor	3.01	−3.4
	O 37	OD2 ASP 855 (A)	H-donor	3.28	−0.9
	O 36	NZ LYS 745 (A)	H-acceptor	3.05	−5.2
	O 37	NZ LYS 745 (A)	H-acceptor	3.06	−4.0
	5-ring	CD2 LEU 844 (A)	pi-H	3.58	−0.7

**TABLE 5:** Antibacterial activity (inhibition zone diameter in mm) of the tested compounds.

Sample ID	MW (g/mol)	Molecular Formula	cLog*P*	Inhibition zone diameter (mm)	
				*Staphylococcus*	*Escherichia coli*
6a	432.42	C_15_H2_21_N_5_O_8_S	−3.44	17	15
6b	432.42	C_15_H2_21_N_5_O_8_S	−3.44	25	15
6c	432.42	C_15_H_21_N_5_O_8_S	−3.44	30	30
7a	351.39	C_14_H_16_N_5_O_4_S	0.120	20	20
7b	373.39	C_16_H_15_N_5_O_4_S	1.31	20	20
8a	399.39	C_17_H_13_N_5_O_5_S	−0.48	13	15
8b	714.97	C_17_H_9_N_5_SO_5_Br_4_	2.42	15	12
9	299.31	C_10_H_11_N_5_O_4_S	−2.55	13	10
10a	461.45	C_16_H_21_N_5_O_9_S	−5.02	35	30
Control	—	—	—	15^a^	40^b^

a cefoxitin for Gram (+ve) bacteria.

b chloramphenicol for Gram (−ve) bacteria.

#### 2.5.1 Identification of pharmacophore sites

Once again*,* POM theory confirms that it is beneficial to identify various and different pharmacophore sites as (antibacterial/antiviral/antifungal/antitumor/antiparasitic).

Thus, POM programs act as a new efficient bioinformatics platform. It is possible to process practically all organic and most organometallic compounds, which will lead to identifying their pharmacophore sites and their optimization, as shown in Figure 7.

Based on geometrical*,* physicochemical parameters of each site and electronic charge repartition of the corresponding X*,* Y*,* and Z heteroatoms (Sogabe et al., 2013; El Ati et al., 2019; ELMeskini et al., 2019; Guerf et al., 2020; Rachedi et al., 2020; Titi et al., 2019; Rad et al., 2017 and Pezzuto et al., 1991).

**FIGURE 8:**
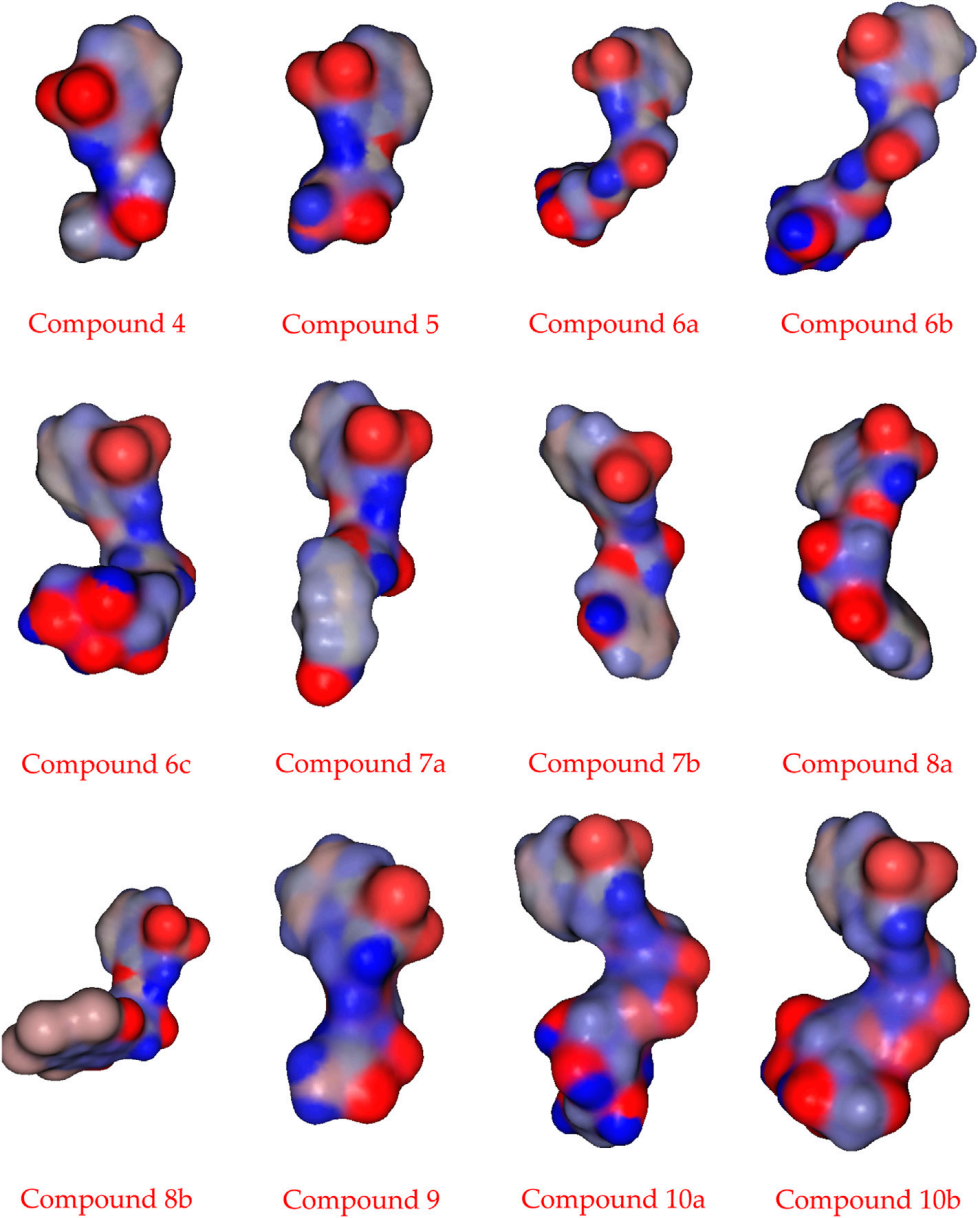
Geometric form of compounds.

**FIGURE 9:**
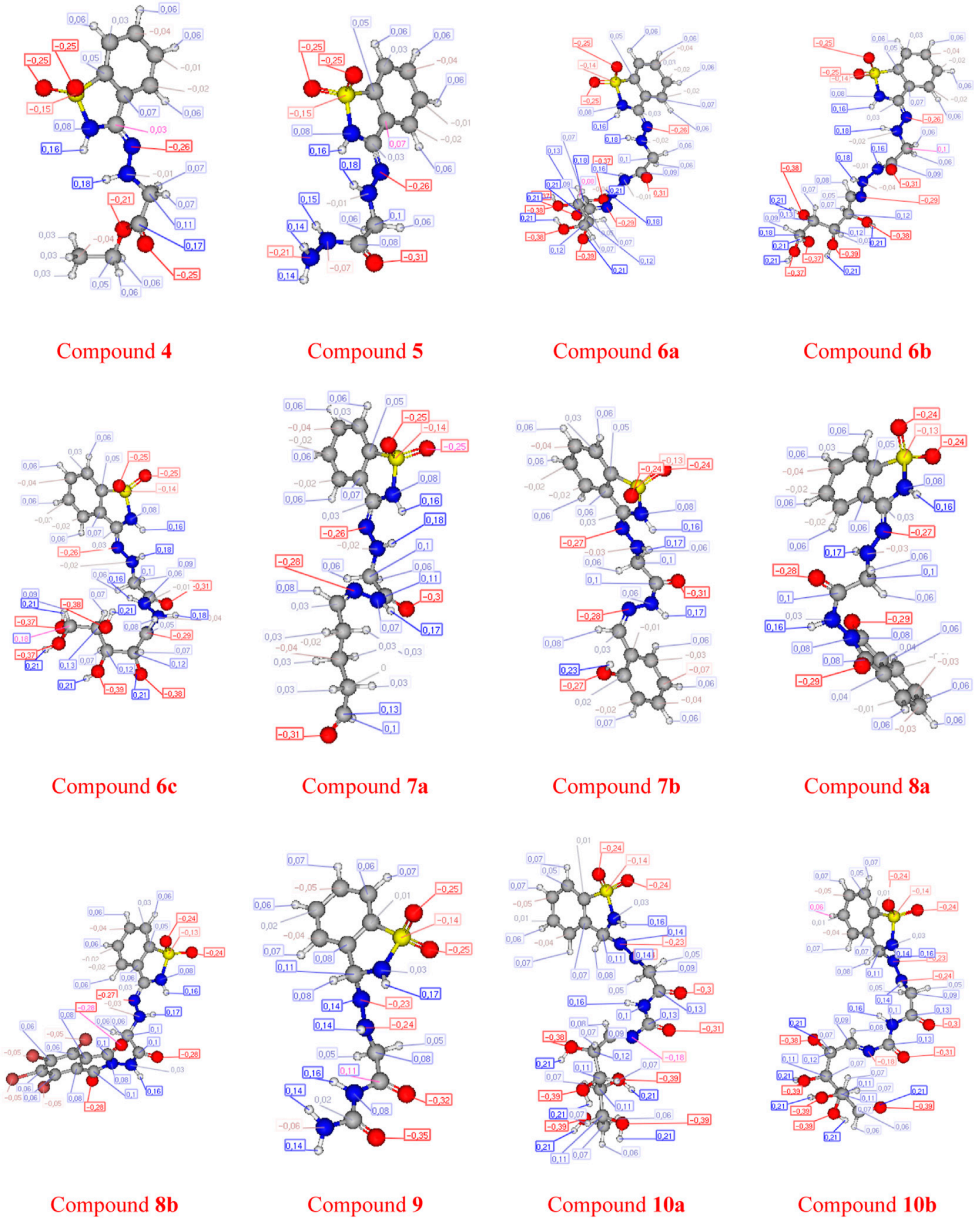
Atomic charge calculation of compounds.

The analysis of conformations and the calculation of the atomic charge shown in Figures 8, 9, of the tested compounds are quite helpful in predicting the efficiency of a drug to the target interaction and, simultaneously, gaining insight into the drug potency and postulate about the favorable conformation of a drug and the antibacterial/antitumor pharmacophore sites. In this study, compounds **6a** and **10a** were shown to be more efficient in inhibiting bacteria than the rest of the compounds tested, resulting in the experimental data reported here.

The POM analyses of the compounds **6b**, **6c,** and **10a** show a good drug score (DS = 83, 83, and 80%, respectively). In addition, the same compounds represent a modest but positive value of protease inhibition (PI = 11, 11, and 8%, respectively).

### 2.6 MD simulations

The molecular dynamic simulation was carried out to elucidate the stability of protein–ligand complexes. The protein–ligand complexes were subjected to a period of 2ns (t). Accordingly, the MD simulation used Desmond for the four docked complexes. The trajectory analysis of docked complexes was carried out by computing the RMSD (root mean square deviation) and RMSF (root mean square fluctuation) values.

#### 2.6.1 RMSD analysis

**FIGURE 10:**
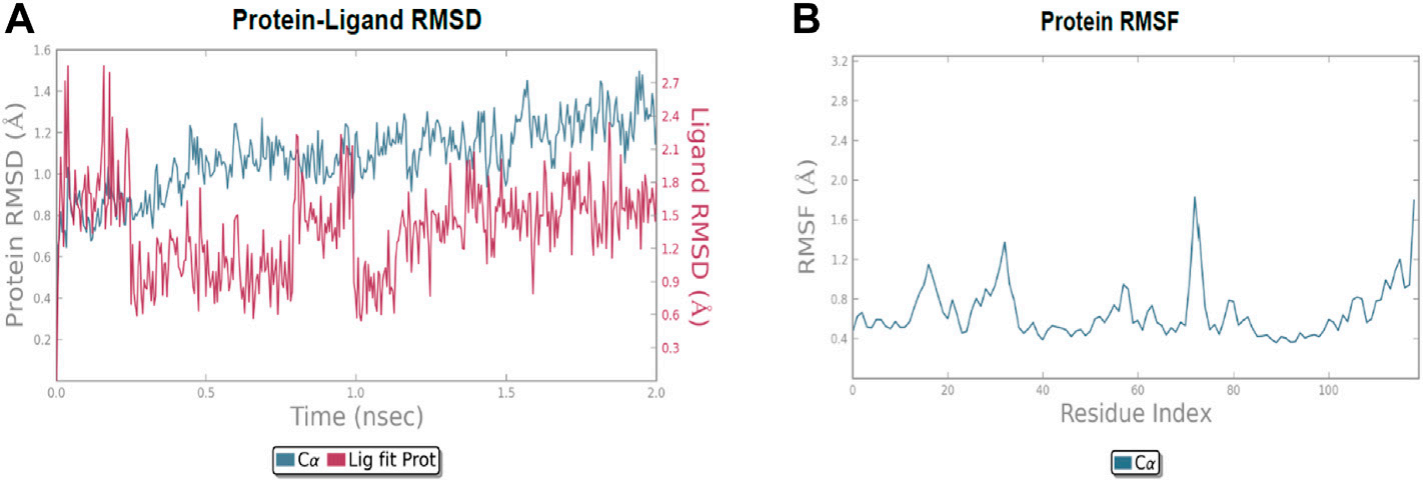
**(A)** RMSD of protein–ligand (**10a** 2OPZ) throughout 2ns and **(B)** RMSF of protein.

**FIGURE 11:**
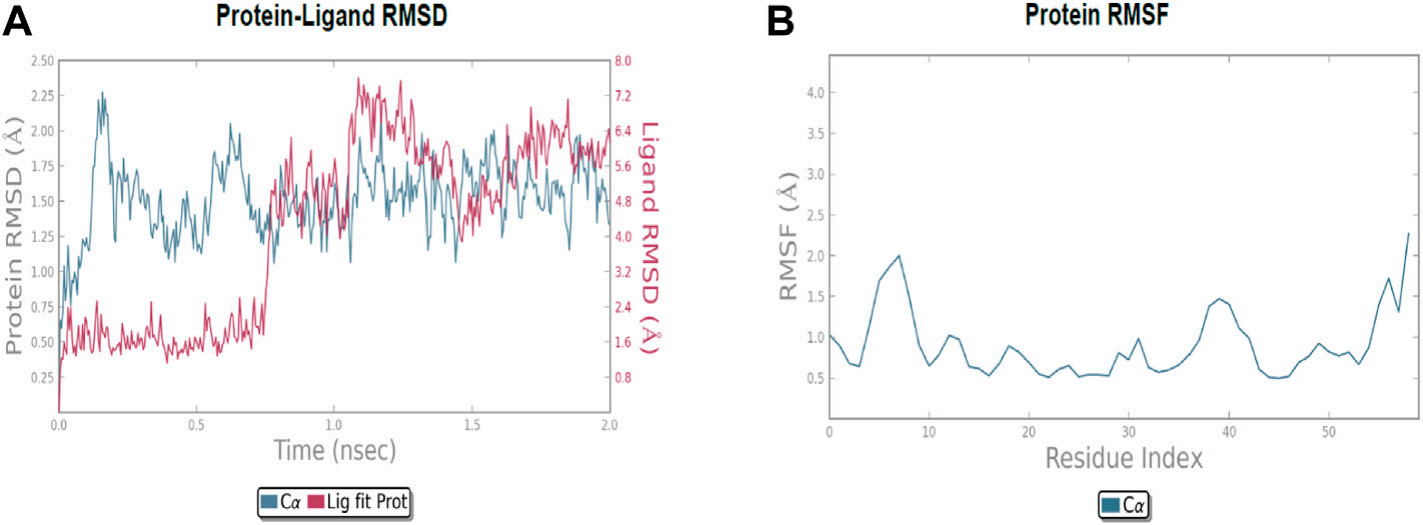
**(A)** RMSD of protein-ligand (6b 2OPZ) throughout 2ns and **(B)** RMSF of protein.

**FIGURE 12:**
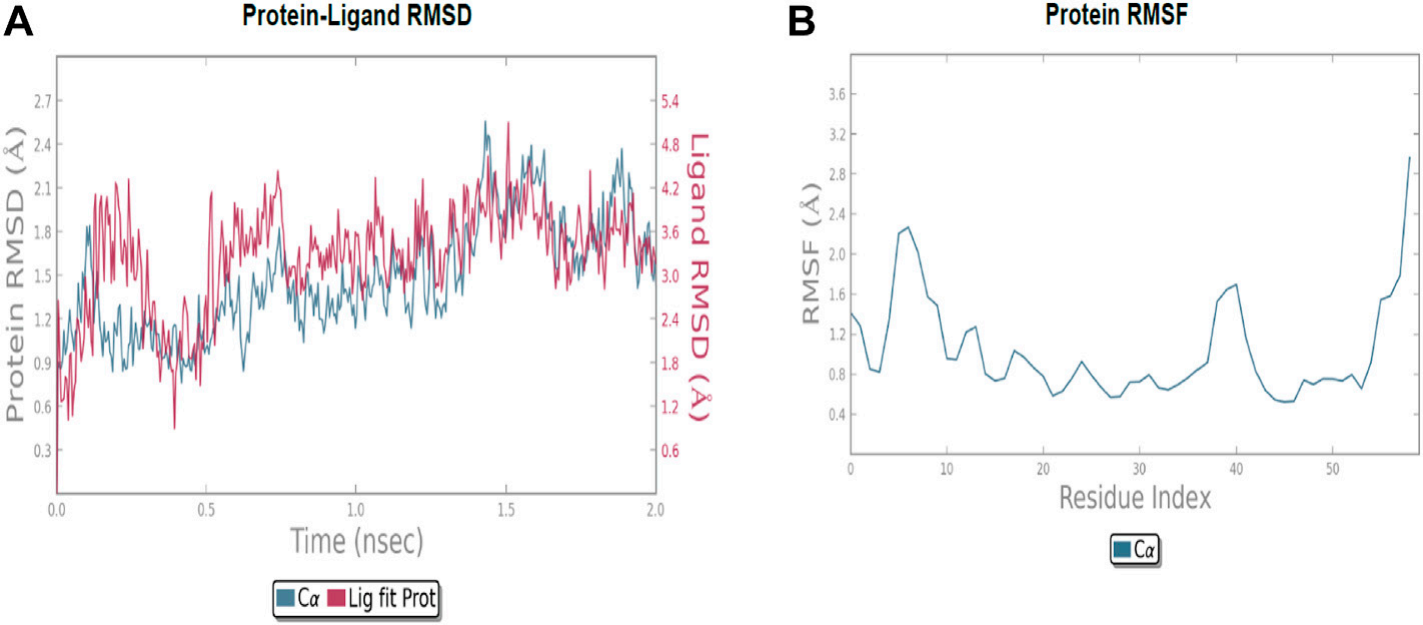
**(A)** RMSD of protein–ligand (**6b** 3W2S) throughout 2ns and **(B)** RMSF of the protein.

**FIGURE 13:**
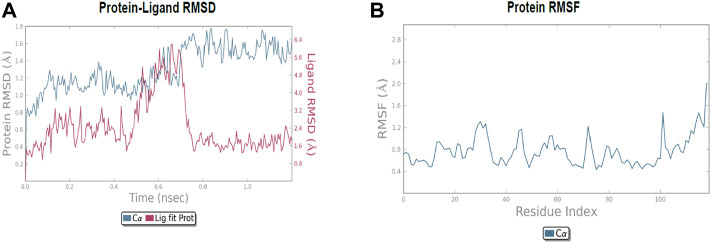
**(A)** RMSD of protein-ligand (**6c** 3W2S) throughout 2ns and **(B)** RMSF of protein.

RMSD is the ultimate measurement for analyzing the stability of MD trajectories. To validate the complex structure (protein–ligand), RMSD was plotted for each system with a time of 2ns throughout the simulation. The RMSD values for the trajectories of each system were found to be stable during the simulation. The total simulation period was 2ns for all the protein complex **10a** 2OPZ. Figure 10A The RMSD value started increasing from the beginning with the range of 1.0–1.6 (Å) for a period of 0.3 ns. The minimum deviation was observed with a range of 0.3–0.8 (Å) for a period of 0.3–0.8 ns. The value of RMSD ranges from 1.3 to 1.6 (Å) for a period of 0.5–0.7 ns with the **6b** 2OPZ complex, as shown in Figure 11. It shows less stability with a minimum deviation from 1 to 1.6 (Å) for 0.7–2.0. ns. On the other hand, the **6b** 3W2S complex in Figure 12A simulation showed a minor deviation for 2ns with a range between 0.9 and 3.2 (Å). In contrast, **6b** 3W2S revealed that RMSD value ranges from 0.3 to 1.1 (Å) for the total simulation period. The RMSD values for acostatin with the **6c** 3W2S complex given in Figure 13A are in the range of 0.75–1.5 (Å) for a period of 0–0.75 ns where the ligand is loosely associated with protein with much deviation.

#### 2.6.2 RMSF analysis

RMSF determines the fluctuation in residues for each system over a given time period during the molecular dynamics simulation. In common, the higher RMSF value indicates more flexibility of the residues, whereas the lower RMSF indicates lower flexibility. The most fluctuating residues for the **10a** 2OPZ, as shown in Figure 10B, complex were found within 16–20, 30–35, and 70–75 residues with RMSF values of 1.2 (Å), 1.5 (Å), and 2.0 (Å), respectively. The fluctuations of the amino acid residues were observed between the residues 25–30, 45–50, 70–75, and 100–103, with values of 1.3 (Å), 1.2 (Å), 1.3 (Å), and 1.6 (Å), respectively, on **6b** 2OPZ complexes, Figure 11B. For the **6b** 3W2S complex, Figure 12B reveals RMSF values of 2.1 (Å) and 1.6 (Å) with minimum fluctuations that showed limited flexibility between 5–10 and 38–44 residues. MD simulation for the **6c** 3W2S complex, Figure 13B, showed less fluctuation amino acid residues with RMSF values of 2.0 (Å) between 70 and 75 residues.

### 2.7 Biological study

#### 2.7.1 Antimicrobial activity

From the results obtained in Table 6, one can notice that compounds **6b, 6c, and 10a** have the highest inhibition zone diameter for ***Staphylococcus,*** while compounds 6c and 10a have the highest inhibition zone diameter for ***Escherichia coli***, which indicates that **10a** is the most effective compound, and this result is matched with the results obtained from the theoretical results.

**TABLE 6:** Anticancer activities of selected compounds in DMSO solutions.

Compound	IC50 ± SD (µM)	
	M-14	Ovcar-3
6a	16.59 ± 0.08	16.76 ± 0.16
6b	10.03 ± 0.43	9.29 ± 0.05
6c	10.6 ± 0.06	10.22 ± 0.01
6d	16.17 ± 0.05	18.09 ± 0.81
7a	12.84 ± 0.43	11.79 ± 0.31
7b	11.47 ± 0.32	11.00 ± 0.28
8a	15.18 ± 0.06	15.25 ± 0.06
8b	10.06 ± 0.06	9.64 ± 0.24
9	14.06 ± 0.87	14.02 ± 0.40
10a	8.66 ± 0.01	7.64 ± 0.01
CisPt	8.96 ± 0.26	7.94 ± 0.43

#### 2.7.2 Antitumor activity of compounds

**FIGURE 14:**
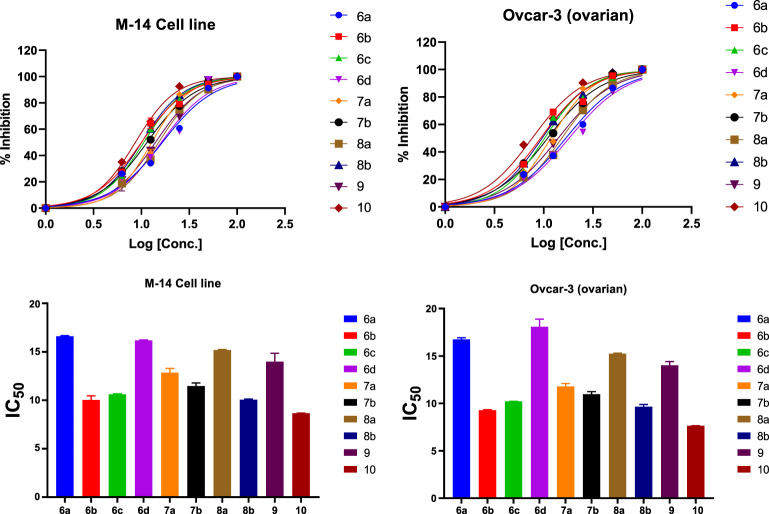
% of inhibition and IC_50_ of selected compounds in DMSO solutions toward M-14 and Ovcar-3 cell lines.

The synthesized compounds were tested on 2 cell lines, namely, **Ovcar-3** and **M-14**, and their efficacies agree with the results obtained by the theoretical studies. The **Ovcar-3** cell line is well-established and one of the most cited model systems for ovarian carcinoma. Skin cancer is another common cancer; the synthesized compounds were tested on a melanoma skin cell line **(M-14**). The IC_50_ results for the **M-14** and **Ovcar-3 cell lines** are matched well in Figure 14, the results were obtained by molecular docking, and the most highly active compounds are **6b, 6c, 8b, and 10a.** The results obtained were compared with cisplatin as a reference control. Cisplatin is a drug used in treating bladder, head, neck, lung, ovarian, and testicular cancers. The drug mechanism of action is primarily through DNA-binding, which prevents the transcription process in the cancer cell. The IC50 values of compounds 6b, 6c, and 10a for both cell lines are compatible with those of cisplatin and are comparable to the cytotoxic effect of cisplatin.

## 3 Materials and methods

All reactions were performed, excluding moisture, dried solvents, and uncorrected melting points. The IR spectra were recorded as potassium bromide pallets on an Aldrich FT-IR spectrometer (Central Laboratory at Faculty of Science, Benha, Ain Shams, and Cairo Universities). Mass spectra were recorded on GCMS (gas chromatography–mass spectrometer) Shimadzu QP- 2010 Plus (Microanalytical center, Ain shams University). Elemental analysis was determined using UV light on the Ain Shams University elementary analysis system. The Bruker Spectro spin DPX-400MHz was used to record the ^1^H NMR (500 MHz, DMSO-*d*_6_) and ^13^C NMR (125 MHz, Chloroform-*d*) spectra. Chemical shift (d) values were stated in parts per million (ppm) using internal standard tetramethylsilane. The D_2_O exchange confirmed that the exchangeable protons (OH and NH) and some other labial hydrogens are exchangeable. LC–MS/MS (PerkinElmer) was used to record the mass spectra, presented as m/z. Elemental analysis was achieved using the PerkinElmer 240 analyzer. The purity of synthesized compounds and the progress of the reaction were evaluated by ascending thin layer chromatography (TLC) (silica gel Fluka, 706, 43-50 EA) using methanol/chloroform (9:1 v/v) and methylene chloride/chloroform (4:1 v/v) as a solvent system.

### 3.1 Chemistry

#### 3.1.1 Synthesis of compounds 1–5

Synthesis of (Z)-3-hydrazineylidene-2,3-dihydrobenzo [d]isothiazole 1,1-dioxide (1).

Saccharin 1.5 g was dissolved in 30 ml of ethanol and a few drops of glacial acetic acid. Then, 3 ml of hydrazine hydrate was added dropwise. The reaction mixture was heated under reflux for 12 h. The solid formed was filtered off, dried, and recrystallized from methanol as white crystals in yield (76%); m. p. 187–190°C. IR (KBr) ʋ cm^−1^: 3323 NH, aromatic CH, 1,659, C = N, and 1340 for SO_2_. ^1^H NMR δ: 7.98 (m, *J* = 7.5, 1.5 Hz, 1H), 7.83 (m, *J* = 8.6, 1.4 Hz, 1H), 7.60 (m, *J* = 7.5, 1.5 Hz, 1H), 7.44–7.37 (m, 2H), and 6.66 (s, 2H). ^13^C NMR δ: 139.70, 134.85, 133.48, 131.13, 130.51, 124.84, and 120.74. Elemental analysis calculated for C_7_H_7_N_3_SO_2_ (197); C, 42.63; H, 3.55; N, 21.31. Found: C, 42.60; H, 3.52; N, 21.27%.

Synthesis of (Z)-((1,1-dioxidobenzo [d]isothiazol-3(2H)-ylidene)amino)glycine (2).

Hydrazino derivative **1** (0.01 mol) and chloroacetic acid (0.012 mol) were dissolved in xylene and refluxed for 6 h. The solid product was filtered under hot conditions and the filtrate was allowed to cool. The solid product was recrystallized from xylene as orange crystals in yield (65%); m. p. 170–172°C. IR (KBr) ʋ cm^−1^: 3,423 (broad) OH of acid, NH, aromatic CH), 2,920, aliphatic CH, 1717, CO of acid 1637 C=N, 1,398 and 1258 for the SO_2_ group. ^1^H NMR δ: 8.04–7.95 (m, 2H), 7.78 (t, *J* = 6.9 Hz, 1H), 7.64 (m, *J* = 7.5, 1.5 Hz, 1H), 7.57–7.48 (m, 2H), and 3.82 (d, *J* = 7.0 Hz, 2H). ^13^C NMR δ: 172.10, 137.06, 134.69, 133.33, 130.53, 129.13, 123.85, 120.93, and 50.08. Elemental analysis calculated for C_9_H_9_N_3_O_4_S (255); C, 42.35; H, 3.53; N, 16.47. Found: C, 42.31; H, 3.50; N, 16.42%.

Synthesis of ethyl (Z)-((1,1-dioxidobenzo [d]isothiazol-3(2H)-ylidene)amino)glycinate (4).

Treatment of [(2Z)-2-(1,1-dioxo-1,2-dihydro-3H-1λ^6^, 2-benzothiazol-3-ylidene) hydrazinyl] acetic acid **2** (10 mmol) with few drops from concentrated sulfuric acid in 15 ml of absolute ethanol. The mixture was heated under reflux for 2 h. The solid product was recrystallized from ethanol as brown crystals in yield (75%); mp. 80–82°C. IR (KBr) ʋ cm^−1^: 3,384, 3,273, NH, 3,093 aromatic CH, 2,980 aliphatic CH, 1720 CO, 1,592, C=N, and 1,336, 1,256 for the SO_2_ group. ^1^H NMR δ: 8.04–7.95 (m, 1H), 7.78 (t, *J* = 6.5 Hz, 1H), 7.64 (m, *J* = 7.5, 1.5 Hz, 1H), 7.57–7.48 (m, 1H), 4.13 (q, *J* = 6.6 Hz, 1H), 3.82 (d, *J* = 6.5 Hz, 1H), and 1.21 (t, *J* = 6.6 Hz, 2H). ^13^C NMR δ: 170.96, 137.07, 134.69, 133.33, 130.53, 129.13, 123.85, 120.93, 61.39, 50.07, and 14.14. Elemental analysis calculated for C_11_H_13_N_3_O_4_S (283); C, 46.64; H, 4.59; N, 14.84. Found: C, 46.60; H, 4.50; N, 14.81%.

Synthesis of (Z)-2-(2-(1,1-dioxidobenzo [d]isothiazol-3(2H)-ylidene)hydrazineyl)acetohydrazide (5).

Ethyl [(2Z)-2-(1,1-dioxo-1,2-dihydro-3H-1λ^6^, 2-benzothiazol-3-ylidene) hydrazinyl] acetate **4** (3 g) was dissolved in 40 ml of ethanol and a few drops of glacial acetic acid, then 3 ml of hydrazine hydrate was added dropwise. The reaction mixture was heated under reflux for 12 h. The solid formed was filtered off, dried, and recrystallized from ethanol as white crystals in yield (78%); m. p. 210–212°C. IR (KBr) ʋ cm^−1^: 3,441 (broad) NH–NH_2_, 3,167, NH, 3,018, aromatic CH, 2,898, aliphatic CH, 1,661 for C=N and 1,661, CO (imide), 1,601, C=N and 1,330, 1,206 cm SO_2_. ^1^H NMR δ: 8.95 (t, J = 3.8 Hz, 1H), 8.04–7.95 (m, 2H), 7.64 (m, J = 7.5, 1.5 Hz, 1H), 7.57–7.48 (m, 3H), 4.25 (d, J = 3.7 Hz, 2H), and 3.68 (d, J = 6.4 Hz, 2H).^13^C NMR δ: 169.86, 137.45, 134.69, 133.33, 130.53, 129.12, 123.85, 120.93, and 49.53. Elemental analysis calculated for C_9_H_11_N_5_O_3_S (269); C, 40.14; H, 4.09; N, 26.02. Found: C, 40.11; H, 4.05; N, 26.00%.

General procedure for the preparation of sugar hydrazine (6–10).

The hydrazide derivative (2.69, 10 mmol) in ethanol (15 ml) was added to a well-stirred solution of the respective monosaccharides (10 mmol) in water (2 ml) and glacial acetic acid (1 ml). The mixture was heated under reduced pressure (water pump) and left to cool. The formed precipitate was washed with water and cold ethanol, dried, and recrystallized from ethanol to afford the corresponding sugar hydrazone in 80–90% yield.

Synthesis of 2-(2-((Z)-1,1-dioxidobenzo [d]isothiazol-3(2H)-ylidene)hydrazineyl)-1-(3-((E)-2,3,4,5,6-pentahydroxyhexylidene)triazaneyl)ethan-1-one (6a).

The use of glucose resulted in compound **6a** as orange solid, m. p. > 300°C. Yield 84%. IR (KBr) ʋ cm^−1^: 3,379 (broad) OH groups, 3,224 (broad) NH, aromatic CH, aliphatic CH groups, 1701 CO, 1,631 (C=N), and 1,336, 1177 for the SO_2_ group. ^1^H NMR δ: 9.21 (d, *J* = 5.1 Hz, 1H), 8.25 (d, *J* = 5.1 Hz, 1H), 8.04–7.95 (m, 2H), 7.64 (td, *J* = 7.5, 1.5 Hz, 1H), 7.57–7.48 (m, 3H), 6.89 (m, *J* = 8.9, 1.7 Hz, 1H), 4.82 (d, *J* = 5.5 Hz, 1H), 4.64–4.56 (m, 2H), 4.51 (d, *J* = 4.8 Hz, 1H), 4.44 (m, *J* = 9.0, 8.1, 4.8 Hz, 1H), 4.35 (d, *J* = 4.8 Hz, 1H), 3.83 (m, *J* = 9.0, 8.0, 5.5, 1.6 Hz, 1H), 3.77–3.70 (m, 2H), 3.68 (d, *J* = 6.6 Hz, 2H), 3.59 (m, *J* = 8.8, 7.2, 4.8 Hz, 1H), and 3.52–3.45 (m, 1H). ^13^C NMR δ: 170.23, 144.67, 137.54, 134.69, 133.33, 130.53, 129.12, 123.85, 120.93, 74.60, 72.22, 72.11, 69.77, 61.56, and 49.51. Elemental analysis calculated for C_15_H2_21_N_5_O_8_S (431); C, 41.76; H, 4.87; N, 16.24. Found: C, 41.70; H, 4.81; N, 16.20%.

Synthesis of 2-(2-((Z)-1,1-dioxidobenzo [d]isothiazol-3(2H)-ylidene)hydrazineyl)-1-(3-((E)-2,3,4,5,6-pentahydroxyhexylidene)triazaneyl)ethan-1-one (6b).

The use of galactose resulted in compound **6b** as a deep yellow semisolid, m. p. > 300°C. Yield 84%. IR (KBr) ʋ cm^−1^: 3,400 (broad) OH groups, 3,338 NH, 3,093 aromatic CH, 2,972 aliphatic CH, 1719, CO, 1592 C = N, and 1,336, 1178 for the SO_2_ group. ^1^H NMR δ: 9.21 (d, *J* = 5.1 Hz, 1H), 8.25 (d, *J* = 5.1 Hz, 1H), 8.04–7.95 (m, 2H), 7.64 (td, *J* = 7.5, 1.5 Hz, 1H), 7.57–7.48 (m, 3H), 6.89 (dd, *J* = 8.9, 1.7 Hz, 1H), 4.82 (d, *J* = 5.5 Hz, 1H), 4.64–4.56 (m, 2H), 4.51 (d, *J* = 4.8 Hz, 1H), 4.44 (ddd, *J* = 9.0, 8.1, 4.8 Hz, 1H), 4.35 (d, *J* = 4.8 Hz, 1H), 3.83 (m, *J* = 9.0, 8.0, 5.5, 1.6 Hz, 1H), 3.77–3.70 (m, 2H), 3.68 (d, *J* = 6.6 Hz, 2H), 3.59 (m, *J* = 8.8, 7.2, 4.8 Hz, 1H), and 3.52–3.45 (m, 1H). ^13^C NMR δ: 170.23, 144.67, 137.54, 134.69, 133.33, 130.53, 129.12, 123.85, 120.93, 74.60, 72.22, 72.11, 69.77, 61.56, and 49.51. Elemental analysis calculated for C_15_H2_21_N_5_O_8_S (431); C, 41.76; H, 4.87; N, 16.24. Found: C, 41.73; H, 4.84; N, 16.23%.

Synthesis of 2-(2-((Z)-1,1-dioxidobenzo [d]isothiazol-3(2H)-ylidene)hydrazineyl)-1-(3-((E)-2,3,4,5,6-pentahydroxyhexylidene)triazaneyl)ethan-1-one (6c).

The use of mannose resulted in compound **6c** as a brown solid, m. p. > 300°C. Yield 79%. IR (KBr) ʋ cm^−1^: 3,340 and 3,234 (broad) for OH and NH groups, respectively, aromatic CH and aliphatic CH groups, 1630 CO, 1583 C=N, and 1335,1150 for the SO_2_ group. ^1^H NMR δ: 9.21 (d, *J* = 5.1 Hz, 1H), 8.25 (d, *J* = 5.1 Hz, 1H), 8.04–7.95 (m, 2H), 7.64 (m, *J* = 7.5, 1.5 Hz, 1H), 7.57–7.48 (m, 3H), 6.89 (dd, *J* = 8.9, 1.7 Hz, 1H), 4.82 (d, *J* = 5.5 Hz, 1H), 4.64–4.56 (m, 2H), 4.51 (d, *J* = 4.8 Hz, 1H), 4.44 (ddd, *J* = 9.0, 8.1, 4.8 Hz, 1H), 4.35 (d, *J* = 4.8 Hz, 1H), 3.83 (m, *J* = 9.0, 8.0, 5.5, 1.6 Hz, 1H), 3.77–3.70 (m, 2H), 3.68 (d, *J* = 6.6 Hz, 2H), 3.59 (m, *J* = 8.8, 7.2, 4.8 Hz, 1H), and 3.52–3.45 (m, 1H). ^13^C NMR: δ 170.23, 144.67, 137.54, 134.69, 133.33, 130.53, 129.12, 123.85, 120.93, 74.60, 72.22, 72.11, 69.77, 61.56, and 49.51. Elemental analysis calculated for C_15_H_21_N_5_O_8_S (431); C, 41.76; H, 4.87; N, 16.24. Found: C, 41.71; H, 4.80; N, 16.22%.

General procedure for the preparation of Schiff base derivatives.

A mixture of hydrazide derivative (2.69 g, 10 mmol) and appropriate aldehyde, namely, glutaric dialdehyde, salicylaldehyde, or anhydride, namely, phthalic anhydride, tetrabromo phthalic anhydride (10 mmol), and glacial acetic acid (1 ml) and 15 ml of ethanol were left under reflux for (4 h). The solid product was filtered after cooling, drying, and crystallizing from ethanol to give compounds in good yield (85–90%) **7a, 7b, 8a, and 8b**.

Synthesis of 2-(2-((Z)-1,1-dioxidobenzo [d]isothiazol-3(2H)-ylidene)hydrazineyl)-1-(3-((E)-pentylidene)triazaneyl)ethan-1-one (7a).

The product obtained was a black crystal, m. p. (240-242) °C. Yield 82%. IR (KBr) ʋ cm^−1^: 3,211 (broad) NH, 3,062, aromatic CH, 2,934; 2,865 aliphatic CH, 1,688; 1629 CO, 1578 C=N, and 1,334, 1,118 for SO_2_. ^1^H NMR δ: 9.23 (d, *J* = 4.9 Hz, 1H), 8.04–7.95 (m, 2H), 7.90 (d, *J* = 4.9 Hz, 1H), 7.68–7.48 (m, 4H), 6.96 (m, *J* = 5.8, 0.9 Hz, 1H), 3.68 (d, *J* = 6.6 Hz, 2H), 2.13 (td, *J* = 8.6, 5.9 Hz, 2H), 1.53 (m, *J* = 8.6, 6.4, 1.0 Hz, 2H), 1.34 (m, *J* = 6.8 Hz, 2H), and 0.86 (t, *J* = 7.0 Hz, 3H).^13^C NMR δ: 170.23, 145.61, 137.54, 134.69, 133.33, 130.53, 129.12, 123.85, 120.93, 49.51, 31.82, 28.21, 22.50, and 13.95. Elemental analysis calculated for C_14_H_16_N_5_O_4_S (350); C, 48.0; H, 4.57; N, 20.0. Found: C, 47.9; H, 4.53; N, 19.9%.

Synthesis of 2-(2-((Z)-1,1-dioxidobenzo [d]isothiazol-3(2H)-ylidene)hydrazineyl)-1-(3-((E)-2-hydroxybenzylidene)triazaneyl)ethan-1-one (7b).

The product obtained was a yellow crystal, m. p. (260-262) °C. Yield 71%. IR (KBr) ʋ cm^−1^: 3383 OH (phenolic), 3217 NH, 3,042 aromatic CH, 2,920 aliphatic CH, 1688 CO, 1572 C=N, and 1,331, 1156 for SO_2_. ^1^H NMR δ: 9.36 (d, *J* = 4.8 Hz, 1H), 8.21 (s, 1H), 8.04–7.95 (m, 2H), 7.68–7.59 (m, 1H), 7.61–7.52 (m, 1H), 7.55–7.48 (m, 1H), 7.50 (s, 1H), 7.37–7.26 (m, 2H), 6.97–6.88 (m, 2H), and 3.68 (d, *J* = 6.6 Hz, 2H).^13^C NMR δ: 170.23, 157.32, 143.75, 137.54, 134.69, 133.33, 132.63, 130.53, 129.94, 129.12, 123.85, 120.93, 117.13, 113.12, and 49.51. Elemental analysis calculated for C_16_H_15_N_5_O_4_S (373): C, 51.47; H, 4.02; N, 18.77. Found: C, 51.43; H, 4.00; N, 18.73%.

Synthesis of 2-(2-(1,1-dioxidobenzo [d]isothiazol-3(2H)-ylidene)hydrazineyl)-N-(1,3-dioxoisoindolin-2-yl)acetamide (8a).

The product obtained was a pale-yellow crystal, m. p. (310-312) °C. Yield 86%. IR (KBr) ʋ cm^−1^: 3,365 cm^−1^, 3,240 cm^−1^ (broad) NH groups, aromatic CH, aliphatic CH, 1770 cm^−1^; 1727 cm^−1^, 1750 cm^−1^ CO, 1,638 cm^−1^ C=N, 1,336 cm^−1^ and 1,159 cm^−1^ for SO_2_. ^1^H NMR (CDCl_3_): ^1^H NMR (500 MHz, DMSO-*d*_6_) δ 8.04–7.95 (m, 2H), 7.91 (q, *J* = 1.1 Hz, 4H), 7.64 (m, *J* = 7.5, 1.5 Hz, 1H), 7.57–7.48 (m, 3H), and 3.70 (d, *J* = 5.7 Hz, 2H). ^13^C NMR δ: 167.17, 164.74, 137.52, 134.69, 134.66, 133.33, 132.64, 130.53, 129.12, 123.85, 123.69, 120.93, and 51.16. Elemental analysis calculated for C_17_H_13_N_5_O_5_S (399); C, 51.13; H, 3.26; N, 17.54. Found: C, 51.11; H, 3.24; N, 17.50%.

Synthesis of 2-(2-(1,1-dioxidobenzo [d]isothiazol-3(2H)-ylidene)hydrazineyl)-N-(4,5,6,7-tetrabromo-1,3-dioxoisoindolin-2-yl)acetamide (8b).

The product obtained was a yellow crystal, m. p. (240-245) °C. Yield 83%. IR (KBr) ʋ cm^−1^: 3,371, 3,326, 3,243 (broad) NH, aromatic CH, aliphatic CH, 1777, 1705 CO, 1638 C=N, 1,336 and 1159 for SO_2_. ^1^H NMR (CDCl_3_): ^1^H NMR (500 MHz, DMSO-*d*_6_) δ 8.04–7.95 (m, 2H), 7.64 (m, *J* = 7.5, 1.5 Hz, 1H), 7.57–7.48 (m, 3H), and 3.70 (d, *J* = 5.7 Hz, 2H).^13^C NMR δ: 167.17, 162.67, 137.52, 134.69, 133.50, 133.33, 130.53, 129.48, 129.12, 123.85, 123.65, 120.93, and 51.16. Elemental analysis calculated for C_17_H_9_N_5_SO_5_Br_4_ (715); C, 28.53; H, 1.26; N, 9.79. Found: C, 28.50; H, 1.22; N, 9.76%.

Synthesis of 2-(2-(1,1-dioxidobenzo [d]isothiazol-3(2H)-ylidene)hydrazineyl)-N-(1,3-dioxoisoindolin-2-yl)acetamide (9).

The mixture of urea (2 mmol) and compound **4** (2.5 mmol) in 40 ml of absolute ethanol and a few drops of glacial acetic acid was refluxed for 12 h. The resulting solution was concentrated, left to cool, and the solid formed was filtered off dried and recrystallized from ethanol as white crystals in yield (72%); m. p. (225-228) °C. IR (KBr) ʋ cm^−1^: 3327 NH, 3,167 (broad for NH and aromatic CH), 2,942 aliphatic CH, 1777, 1705 CO, 1607 C=N, 1,338 and 1,159 for SO_2_.

^1^H NMR δ: 7.98–7.89 (m, 1H), 7.68–7.61 (m, 1H), 7.56–7.46 (m, 2H), 7.22 (s, 2H), 5.88–5.79 (m, 2H), 4.59 (dt, *J* = 5.1, 4.5 Hz, 1H), 3.53–3.40 (m, 2H), and 2.02 (dd, *J* = 5.2, 4.2 Hz, 1H). ^13^C NMR δ: 167.18, 153.89, 136.92, 133.23, 131.93, 127.79, 127.74, 122.96, 63.30, and 52.18. Elemental analysis calculated for C_10_H_11_N_5_O_4_S (297); C, 40.40; H, 3.70; N, 23.57. Found: C, 40.36; H, 3.66; N, 23.54%.

General procedure for the preparation of glycoside derivative.

Compound **9** (2.45 g, 10 mmol) in ethanol (10 ml) was added to a well-stirred solution of the respective monosaccharides (glucose or galactose) in distilled water (2 ml) and glacial acetic acid (1 ml). The mixture was heated under reflux for 5–6 h, and the resulting solution was concentrated under reduced pressure (water pump) and left to cool. The precipitate formed was filtered off, washed with water and cold ethanol, dried, and recrystallized from ethanol to give the corresponding compounds **10a** and **10b,** respectively.

Synthesis of (E)-2-(2-(1,1-dioxido-2,3-dihydrobenzo [d]isothiazol-3-yl)hydrazineyl)-N-((2,3,4,5,6-pentahydroxyhexylidene)carbamoyl)acetamide (10a).

The use of glucose resulted in compound **10a** as white crystals in yield (86%); mp (180-182) °C. IR (KBr) ʋ cm^−1^: 3,406 (broad) for (OH, NH, aromatic CH), 2,931, 2,867 for aliphatic CH, 1774 and 1719 for CO, 1,613 for the C = N, 1,315 and 1125 SO_2_ group, ^1^H NMR δ: 7.98–7.89 (m, 1H), 7.68–7.60 (m, 2H), 7.56–7.46 (m, 2H), 5.88–5.79 (m, 2H), 5.08 (d, *J* = 4.9 Hz, 1H), 4.76 (d, *J* = 5.5 Hz, 1H), 4.69 (m, *J* = 9.3, 8.0, 4.9 Hz, 1H), 4.64–4.55 (m, 3H), 4.23 (d, *J* = 4.8 Hz, 1H), 3.82–3.69 (m, 3H), 3.64–3.44 (m, 4H), and 2.02 (dd, *J* = 5.1, 4.2 Hz, 1H).^13^C NMR δ: 166.41, 162.85, 160.87, 136.92, 133.23, 131.93, 127.76 (d, *J* = 6.7 Hz), 122.96, 74.00, 72.11, 71.11 (d, *J* = 7.6 Hz), 63.30, 61.56, and 52.11. Elemental analysis calculated for C_16_H_21_N_5_O_9_S (459); C, 41.83; H, 4.58; N, and 15.25. Found: C, 41.80; H, 4.54; N, 15.20%.

Synthesis of (E)-2-(2-(1,1-dioxido-2,3-dihydrobenzo [d]isothiazol-3-yl)hydrazineyl)-N-((2,3,4,5,6-pentahydroxyhexylidene)carbamoyl)acetamide (10b).

The use of glucose resulted in compound **10b** as white crystals in yield (86%); m. p. (180-182) ° C. IR (KBr) ʋ cm^−1^: 3,459 (broad) (OH, NH), 2,973, aliphatic CH, 1720, 1672 CO, 1,623, 1,592 for C=N and 1,336, 1,138 for the SO_2_ group. ^1^H NMR δ: 7.98–7.89 (m, 1H), 7.68–7.60 (m, 2H), 7.56–7.46 (m, 2H), 5.88–5.79 (m, 2H), 5.08 (d, *J* = 4.9 Hz, 1H), 4.76 (d, *J* = 5.5 Hz, 1H), 4.69 (m, *J* = 9.3, 8.0, 4.9 Hz, 1H), 4.64–4.55 (m, 3H), 4.23 (d, *J* = 4.8 Hz, 1H), 3.82–3.69 (m, 2H), 3.64–3.49 (m, 5H), and 2.02 (dd, *J* = 5.1, 4.2 Hz, 1H).

^13^C NMR δ: 166.41, 162.85, 160.87, 136.92, 133.23, 131.93, 127.76 (d, *J* = 6.7 Hz), 122.96, 74.00, 72.11, 71.11 (d, *J* = 7.6 Hz), 63.30, 61.56, and 52.11. Elemental analysis calculated for C_16_H_21_N_5_O_9_S (459); C, 41.79; H, 4.55; N, 15.25. Found: C, 41.80; H, 4.54; N, 15.21%.

### 3.2 Modeling and computational studies

#### 3.2.1 DFT theory

Theoretical DFT simulation was achieved in the gas phase using the B3LYP/6-311G (d,p) basis set introduced into Gaussian 9. This included predicting the geometrical optimization on each prepared compound **6a–c, 8a, b, and 10 a–b** to determine the lowest energy molecular structure, followed by calculating the frequency at the optimum geometrical structure, during which several thermochemical parameters were taken into account. Due to the absence of imaginary frequency, all optimized geometrical structures of the prepared compounds (Ali et al., 2018; Rachedi et al., 2019; and Shawon et al., 2018; Jayakumar and Anishetty, 2014; Wist et al., 2007; and Abdellattif et al., 2020) are stable. Therefore, the calculations were carried out for the prepared compounds. This entailed performing a geometrical optimization on each compound to evaluate the minimum energy structure and then calculating the frequency at the optimized geometry while also computing various thermochemical quantities.

#### 3.2.2 Molecular docking

The crystal structures of the proteins identified for the melanoma cancer protein (2OPZ) (Skehan et al., 1990; Shawon et al., 2018 Sogabe et al., 2013) and the ovarian cancer protein (3W2S) were obtained from the Protein Data Bank. Water molecules around the protein were removed, and hydrogen atoms were added. The parameters and charges were assigned with the MMFF94x force field. After alpha-site spheres were generated using the site finder module of MOE, our compound was docked in the active site using the DOCK module of MOE. The dock scoring in MOE software was calculated using the London dG scoring function and refined using two different methods (Table 7). The planarity of the system was maintained, and the best poses were analyzed for the best score (Elkamhawy et al., 2021; Sait et al., 2019).

**TABLE 7:** Docking interaction of all compounds with 2OPZ “melanoma cancer protein.”

Compound	Ligand	Receptor	Interaction	Distance E	(kcal/mol)
6a	N 12	OE1 GLN 319 (A)	H-donor	3.14	−1.7
	N 48	OE2 GLU 314 (A)	H-donor	3.54	−1.3
	O 19	NZ LYS 311 (A)	H-acceptor	2.93	−7.6
	O 31	NZ LYS 322(A)	H-acceptor	3.18	−1.2
6b	N 20	OE1 GLN319 (A)	H-donor	3.19	−1.0
	O 26	O THR 308 (A)	H-donor	2.88	−2.2
	O 31	O THR 308(A)	H-donor	2.84	−2.0
	O 38	N THR 308 (A)	H-acceptor	3.23	−1.4
	C 29	5-ring TRP 323 (A)	H-pi	4.59	−1.0-1.2
6c	N 20	OE2 GLU 314 (A)	H-donor	3.04	
	O 27	O THR 308 (A)	H-donor	3.11	−1.0
	O 31	O THR 308 (A)	H-donor	2.75	−1.3
	O 38	O THR 308 (A)	H-donor	2.85	−2.2
	O 19	NZ LYS 322 (A)	H-acceptor	2.98	−4.3
	O 38	N THR 308 (A)	H-acceptor	3.02	−0.9
7a	C 5	OE1 GLU 314 (A)	H-donor	3.41	−0.5
	O 42	O VAL 298 (A)	H-donor	3.09	−1.3
	6-ring	CD LYS 297 (A)	pi-H	3.81	−0.6
	6-ring	NZ LYS 297 (A)	pi-cation	4.12	−0.9
7b	6-ring	CA GLY 305 (A)	pi-H	3.99	−0.8
8a	C 5	OE1 GLU 314 (A)	H-donor	3.28	−0.5
	N 12	O THR 308 (A)	H-donor	3.00	−4.6
	C 20	6-ring TRP 323 (A)	H-pi	3.90	−0.8
	6-ring	CA GLY 306 (A)	pi-H	3.86	−0.7
8b	N 18	OE2 GLU 314 (A)	H-donor	2.95	−5.1
	N 24	OE2 GLU 314 (A)	H-donor	3.50	−0.5
	O 26	NZ LYS 322 (A)	H- acceptor	2.96	−2.9
	O 40	NE2 GLN 319 (A)	H-acceptor	2.91	−1.8
9	N 26	O THR 308 (A)	H-donor	2.93	−2.3
	N 16	6-ring TRP 323 (A)	H-pi	4.65	−0.5
	6-ring	CA LEU 307 (A)	pi-H	4.33	−0.6
10a	N 20	OE2 GLU 314 (A)	H-donor	3.05	−2.6
	O 35	OE1 GLU 318 (A)	H-donor	2.75	−2.6
	O 29	NZ LYS 311 (A)	H-acceptor	2.96	−6.4
	N 30	NE2 GLN 319 (A)	H-acceptor	3.27	−1.5
	6-ring	N THR 308 (A)	pi-H	4.33	−0.5
1	N 12	OE1 GLN 319 (A)	H-donor	3.07	−1.8
	N 16	OE2 GLU 314 (A)	H-donor	3.30	−1.2
	O 53	OE2 GLU 318 (A)	H-donor	3.29	−0.5
	O 29	NZ LYS 311 (A)	H-acceptor	2.93	−9.9
Reff. M14	N 1	OE2 GLU 314 (A)	H-donor	2.85	−11.0
	N 1	OE1 GLN 319 (A)	H-donor	3.10	−4.8
	O 8	CE LYS 311 (A)	H-acceptor	3.47	−0.5
	O 33	N THR 308 (A)	H-acceptor	2.98	−2.1
	OXT 63	OG1 THR 308 (A)	H-acceptor	2.88	−0.8
	N 1	OE2 GLU 314 (A)	Ionic	2.85	−5.5

#### 3.2.3 MD simulation

After the analysis of the docking results, the most scored docking compounds were selected for further studies using the Desmond software package (David et al., 2005), which was used to study their conformational dynamics under explicit water conditions for a time period of 10 ns time scale. The OPLS-AA force field (Robertson et al., 2015) was used to generate the topologies of the HSA protein in the docked complexes. The LigParGen server (Leela et al., 2017) generated the same force field potentials for the complex inhibitors. Afterward, the systems were immersed in the SPC/E water model (Zielkiewicz, 2005) (Kumari et al., 2014) and neutralized by adding counter Na^+^ and Cl^−^ ions. The further processing involved energy minimization using steepest descent and conjugate gradient algorithms, with a convergence criterion of 0.005 kcal/mol. The minimized systems were subjected to positions restraining and then equilibrated under NVT (constant volume) and NPT (constant pressure) ensemble conditions, each at a 100 ps time scale. The temperature of 300 K was maintained for the system using the Berendsen weak coupling method, and the pressure of 1 bar was maintained utilizing the Parrinello–Rahman barostat in the equilibration stage. Furthermore, the final production stage was carried out using the LINCS algorithm. The generated trajectories were analyzed for protein-inhibitor distances, H-bonds, RMSD, and Rg changes. Finally, the molecular mechanics Poisson–Boltzmann surface area (MM-PBSA) protocols implemented in the g_mmpbsa package (Muanza et al., 1994) were used to calculate binding free energy between the HSA and inhibitors.

### 3.3 Biological study

#### 3.3.1 Antimicrobial activity

The agar diffusion method was used for the bacteria and fungi screening process and was maintained on the nutrient agar and Zapek’s dox agar medium, respectively [10–11]. Assay medium flocks containing 50 ml of nutrient agar for bacteria were allowed to reach 40–50°C to be incubated with 0.5 ml of the cell suspension of the tested organism. The flasks were mixed well and poured each into a Petri dish (15 × 2 cm) and allowed to solidify. The synthesized compounds were dissolved each in 2 ml of DMSO. A measure of 100 ml of each compound was placed in these holes using an automatic micropipette. The Petri dishes were ice-fed at 5°C for 1 h to allow the diffusion of the samples through the agar medium and retarded the growth of the test organism. Plates were incubated at 30°C for 24 h for bacteria. The zone of inhibition diameters was measured and compared with the standard, and the values were tabulated.

#### 3.3.2 Antitumor activity of compounds

The cells were purchased from the Egyptian Holding Company for Biological Products and Vaccines (VACSERA, Giza, Egypt) and kept in a tissue culture unit. The cells were grown in the Roswell Park Memorial Institute (RPMI)–1,640 medium supplemented with 10% heat-inactivated fetal bovine serum (FBS), 50 units/ml penicillin, and 50 mg/ml streptomycin and maintained in a humidified atmosphere containing 5% CO_2_ (Wist et al., 2007 and Abdellattif et al., 2020). The cells were maintained as monolayer cultures using serial subculture. The cell culture reagents were obtained from Lonza (Basel, Switzerland). The anticancer activities of the rested compounds were evaluated in Ovcar-3 (ovarian) and M-14 (melanoma). In addition, the sulforhodamine B (SRB) assay method that was described previously (Pezzuto et al., 1991) was used to determine cytotoxicity. Exponentially growing cells were collected using 0.25% trypsin-EDTA and seeded in 96-well plates at 1,000–2,000 cells/well in the RPMI -1640 supplemented medium. After 24 h, the cells were incubated for 72 h with various concentrations of the compounds tested. Following 72 h of incubation, the cells were fixed with 10% trichloroacetic acid for 1 h at 4°C. Wells were stained for 10 min at room temperature with 0.4% sulforhodamine B (SRBC) dissolved in 1% acetic acid. Plates were air-dried for 24 h, and the dye was solubilized with Tris-HCl for 5 min on a shaker at 1,600 rpm. Each well’s optical density (OD) was measured spectrophotometrically at 564 nm using an ELISA microplate reader (ChroMate-4300, Palm City, FL, United States). The IC_50_ values were calculated using a Boltzmann sigmoidal concentration–response curve using non-linear regression fitting models (GraphPad, Prism Version 9, GraphPad Software, San Diego, CA, United States).

## 4 Conclusion

Acetic acid hydrazide was an essential nucleus to synthesize promising saccharine derivatives for biological activities. Many synthetic compounds were promising, as these compounds **(6b, 6c,** and **10a)** gave interesting biological values. The DFT results have been reported that most synthetic series have high binding affinity significantly compounds (**6b**, **6c**, and **10a**). Those molecular docking findings of highest docking score energy. Antimicrobial assessments represented similar results. The same compounds showed higher IC_50_ values in M-14 and Ovcar-3 cell lines; compound **8b** showed high experimental IC_50_ toward Ovcar-3 cell lines. A comparative study between experimental screening and virtual POM analysis shows good convergence. This study showed that compounds **6b**, **6c**, and **10a** are more efficient at inhibiting bacteria than the rest of the tested compounds’ theories, resulting in the experimental and virtual POM data reported here. Md simulations at 2 n-sec elucidated the stability of protein–ligand complexes.

## Data Availability

The original contributions presented in the study are included in the article/Supplementary Material; further inquiries can be directed to the corresponding authors.

## Abbreviations

MOE, Molecular Operating Environment; MD, molecular dynamics; PDB, Protein Data Bank; POM, Petra/Osiris/Molinspiration.
