# Supplementary material for: Novel saccharin analogs as promising antibacterial and anticancer agents: synthesis, DFT, POM analysis, molecular docking, molecular dynamic simulations, and cell-based assay
Source: Front Pharmacol. 2022 Oct 4;13:958379. doi: 10.3389/fphar.2022.958379 (PMC9577234; doi:10.3389/fphar.2022.958379)
Supplement: Supplementary file 1 [file DataSheet1.pdf]

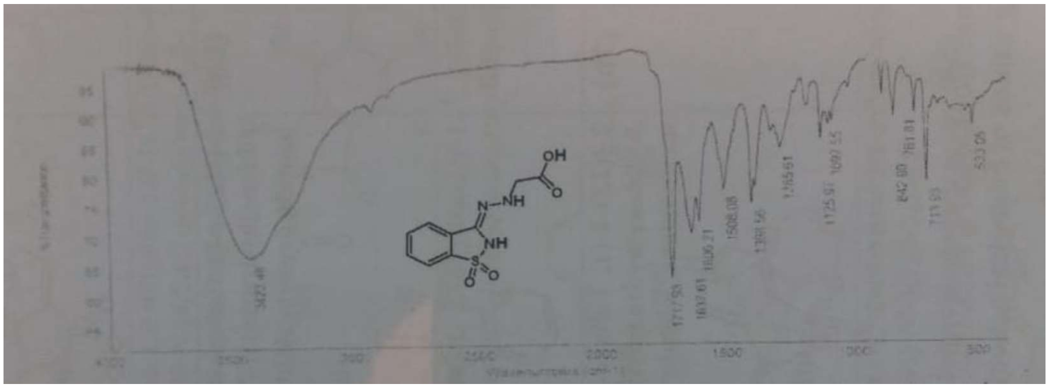

Compound 2 IR

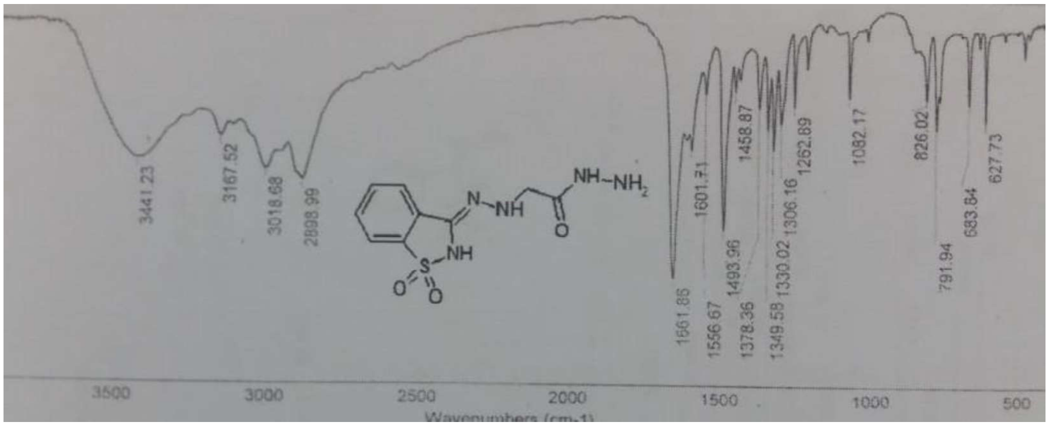

Compound 5 IR

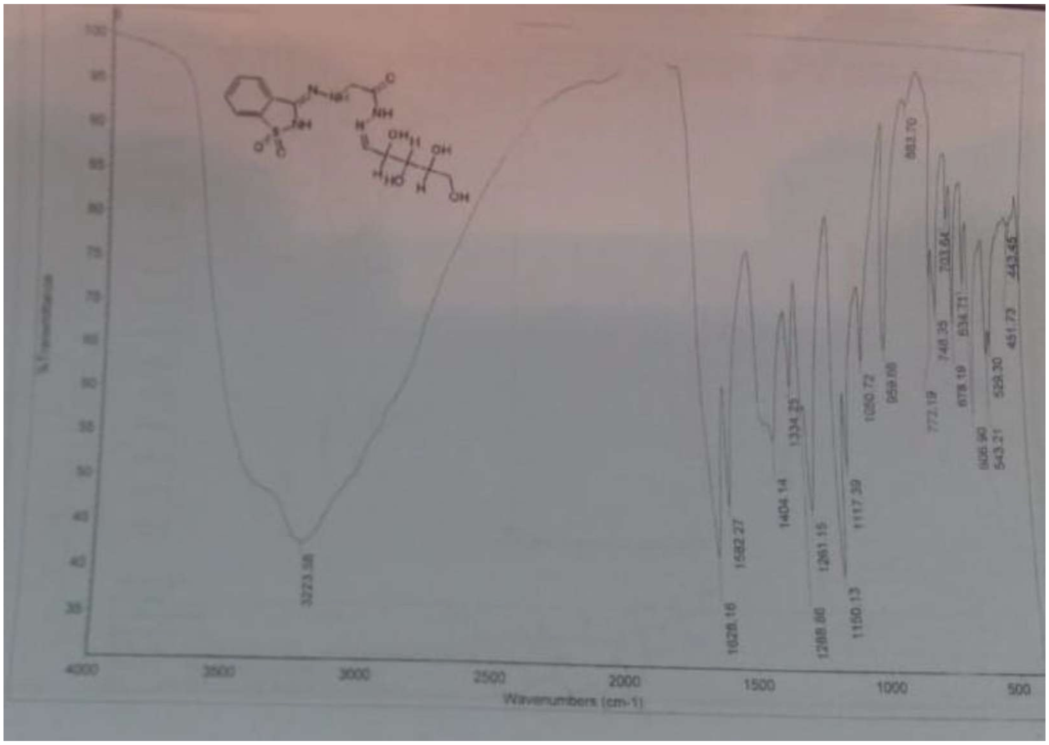

Compound 6a-IR

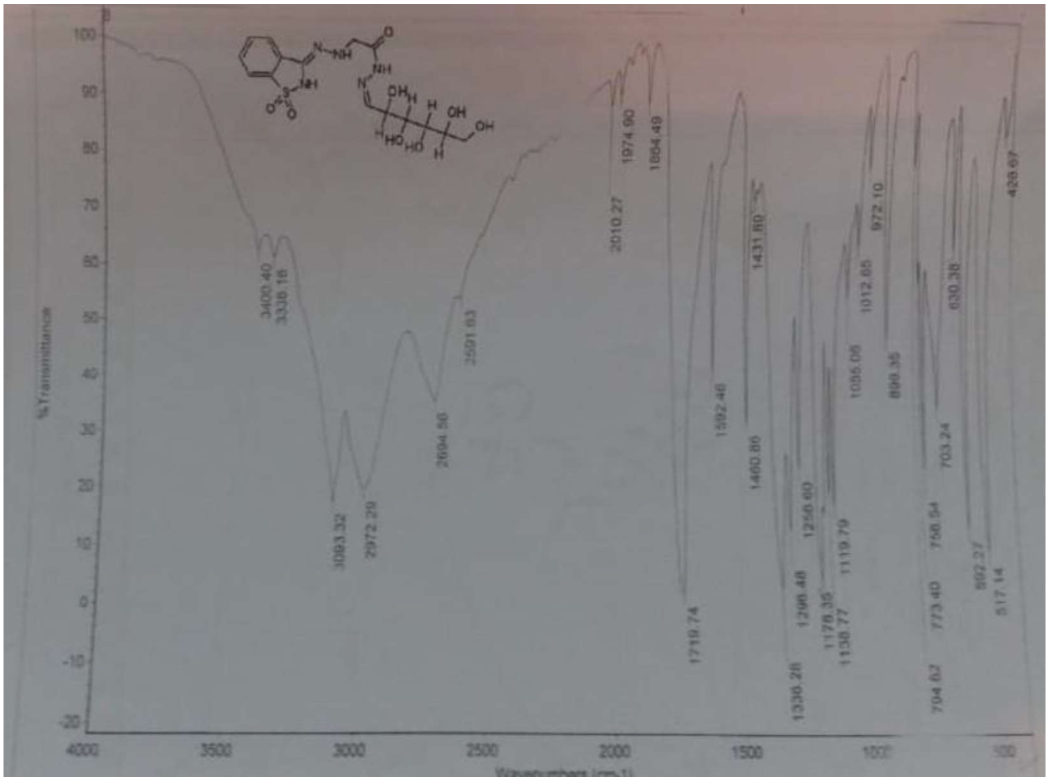

Compound 6b-IR

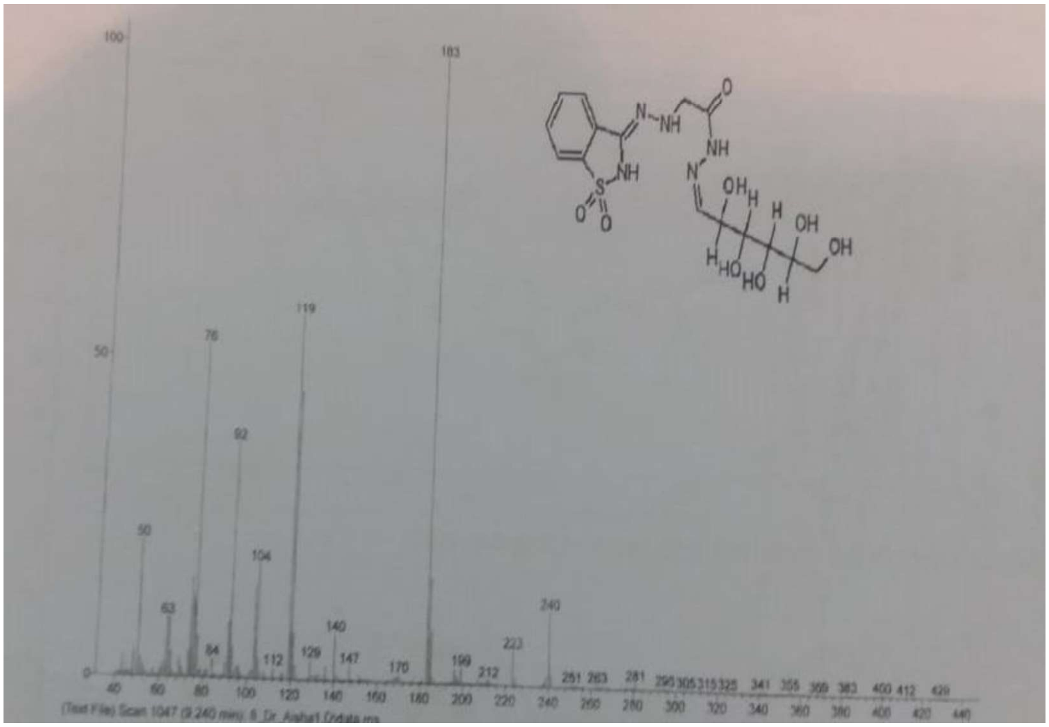

Compound 6b-Mass

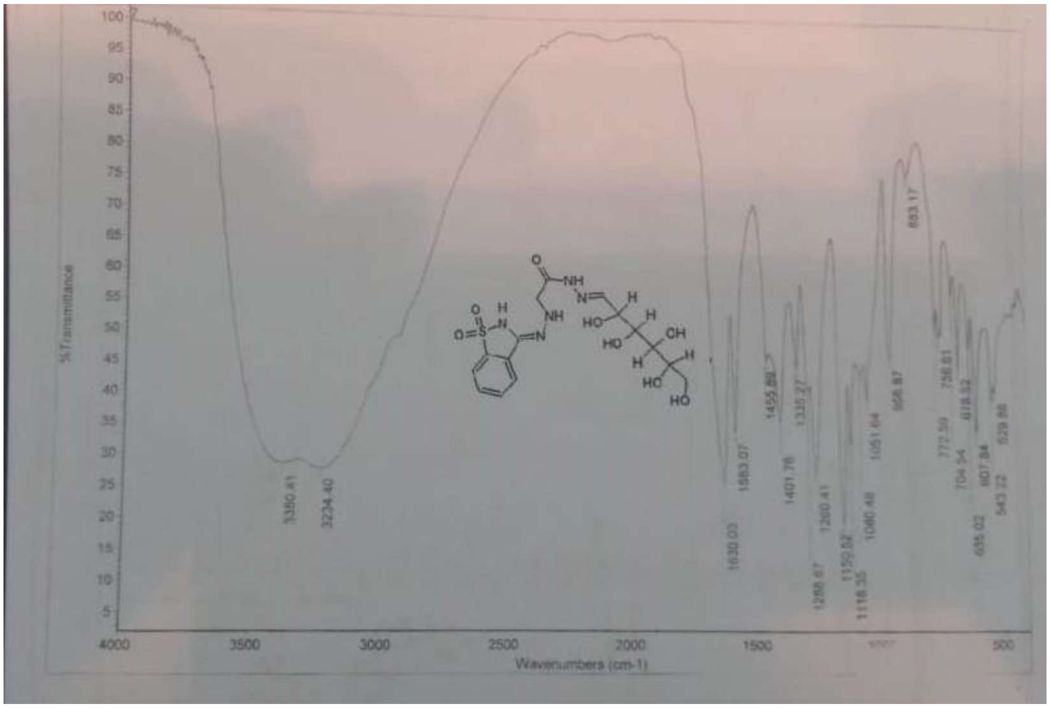

Compound 6c-IR

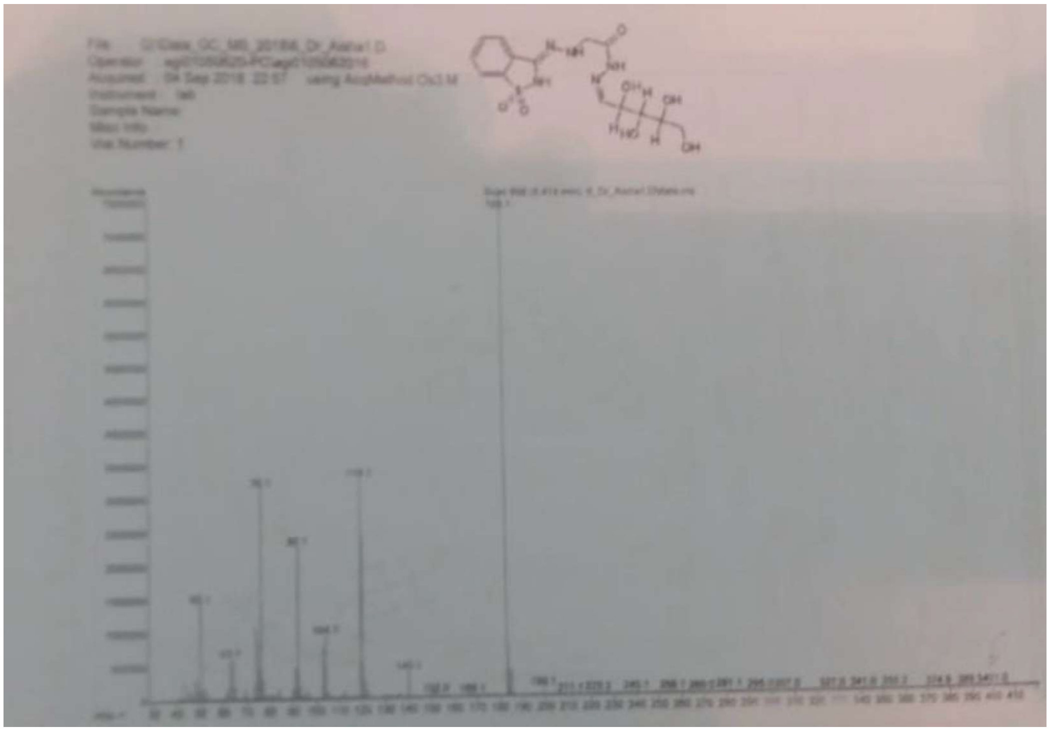

Compound 6d-mass

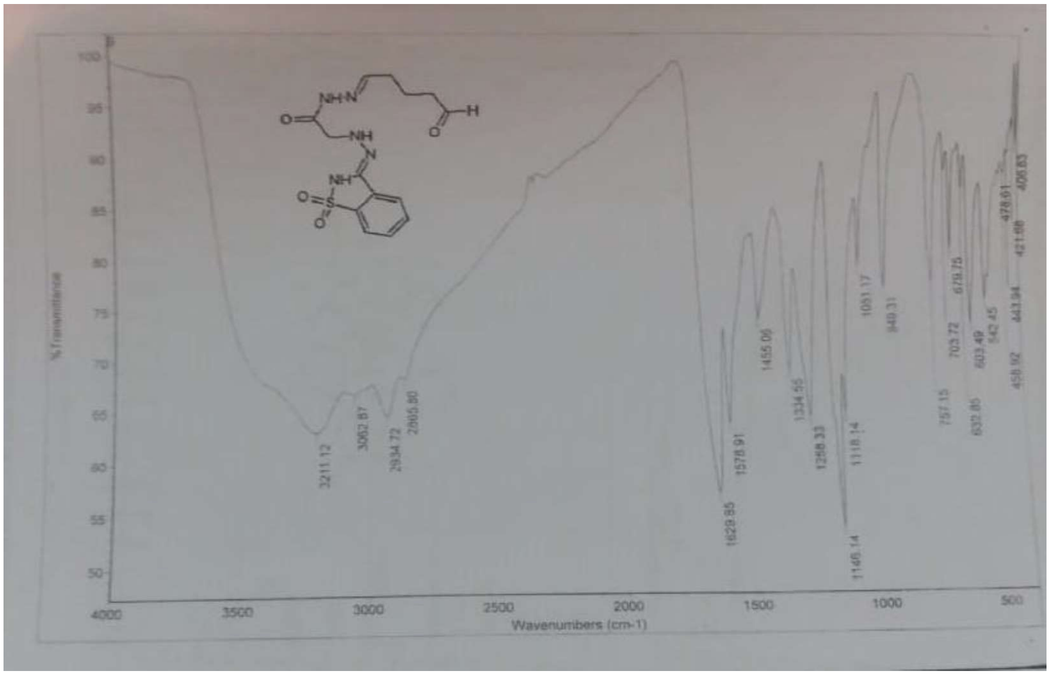

Compound 7a-IR

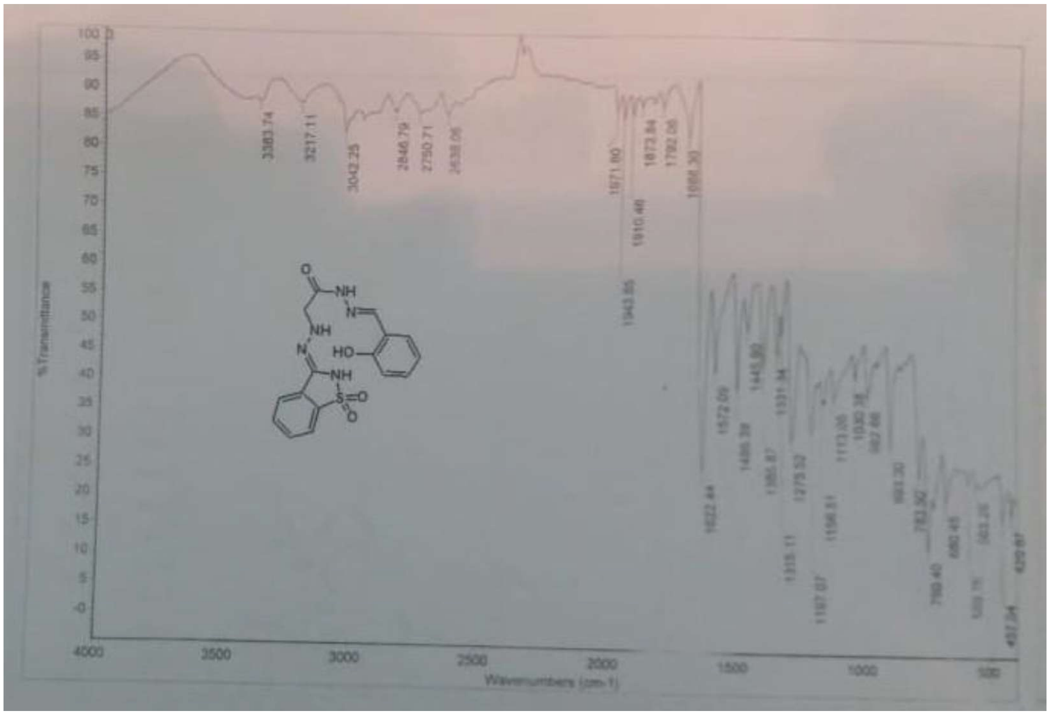

Compound 7b-IR

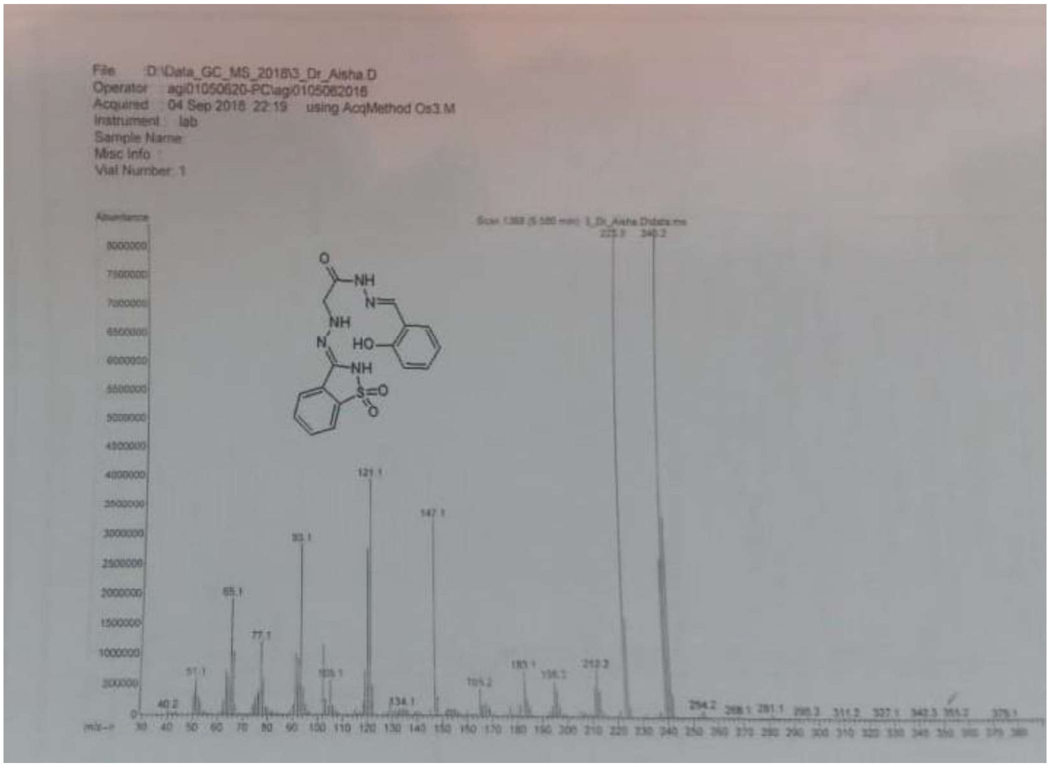

Compound 7b-mass

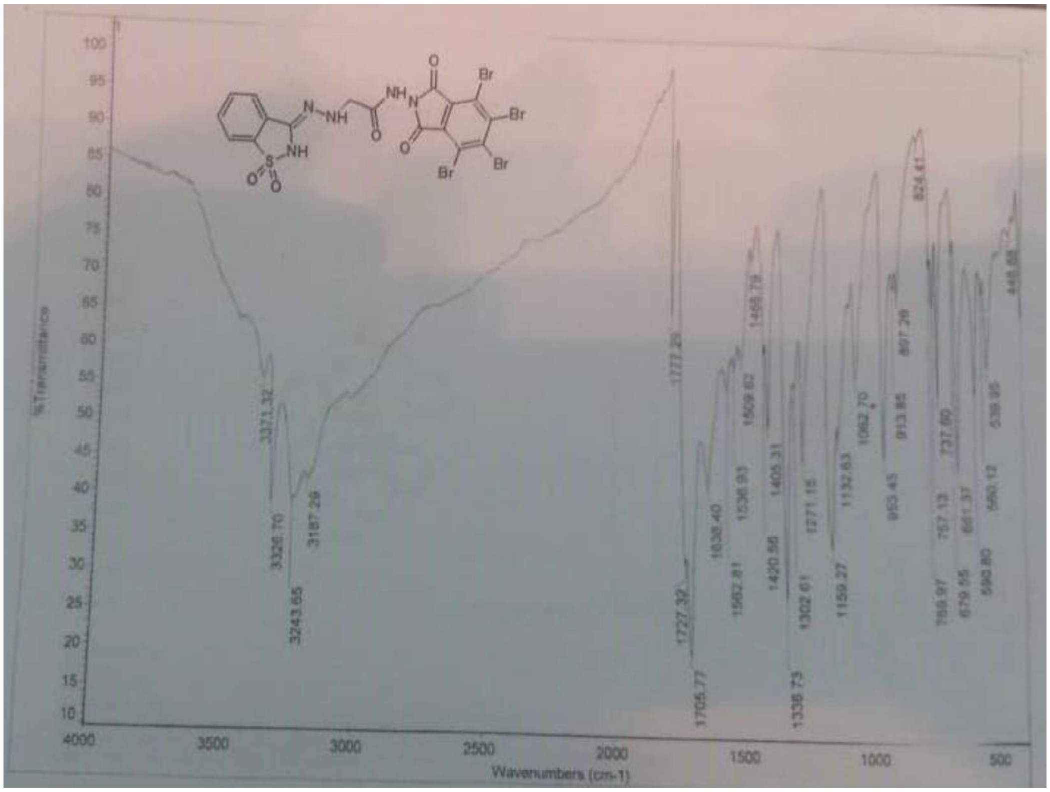

Compound 8b-IR

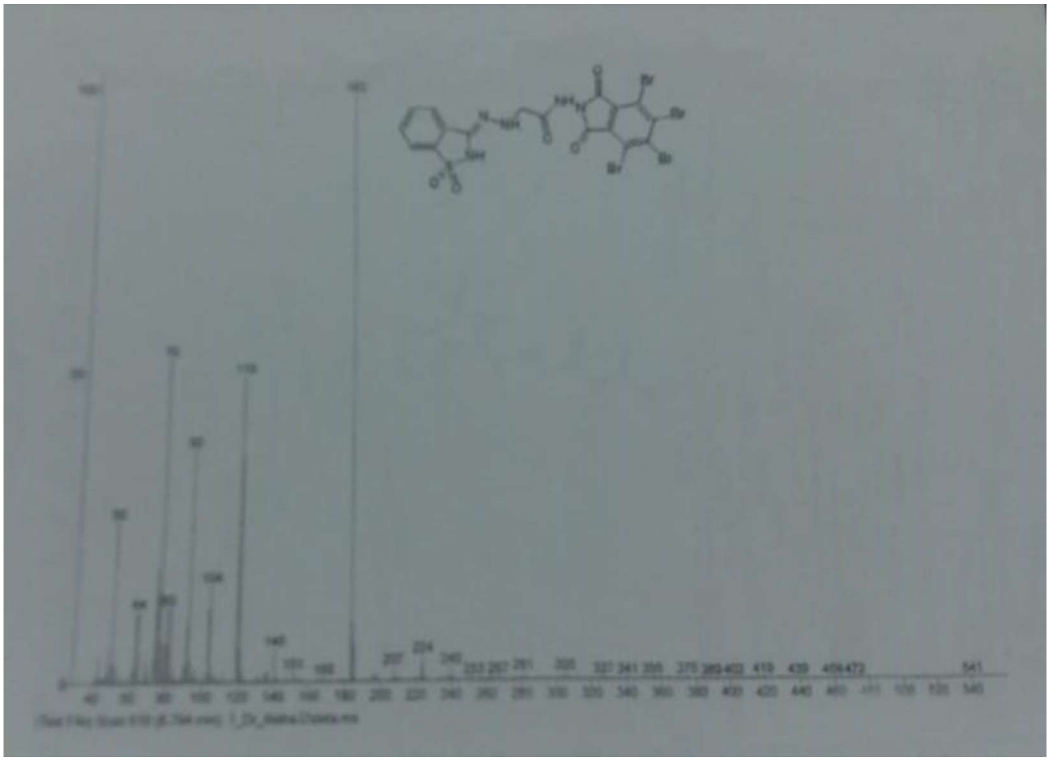

Compound 8b-mass

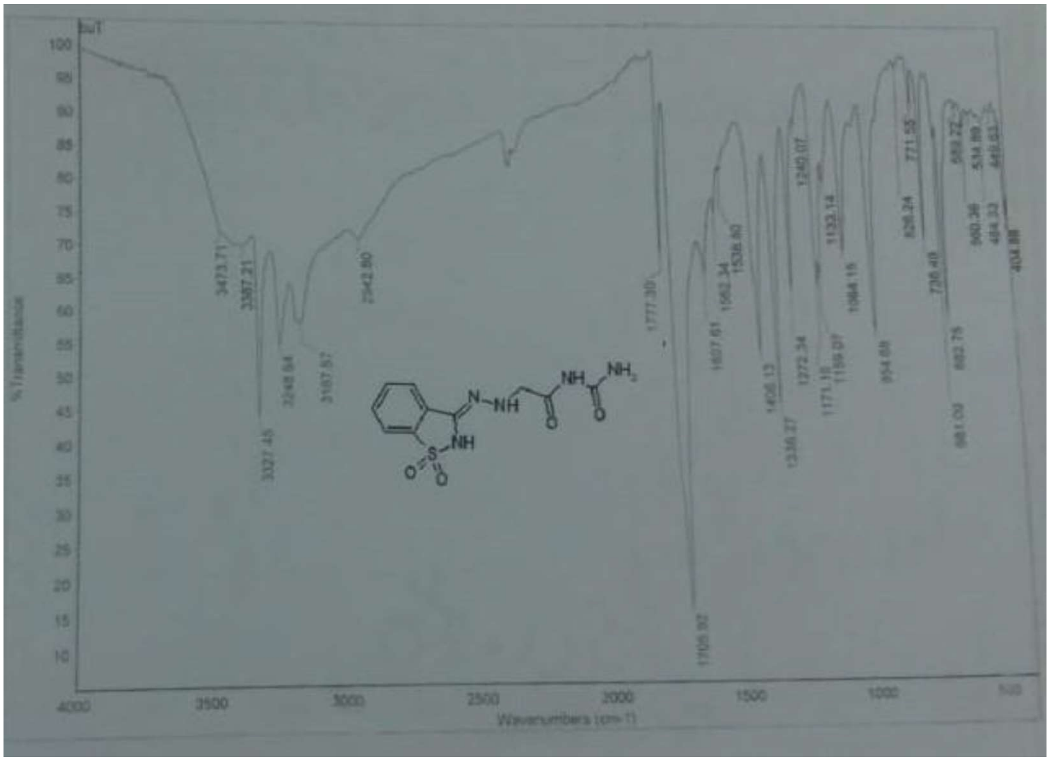

Compound 9-IR

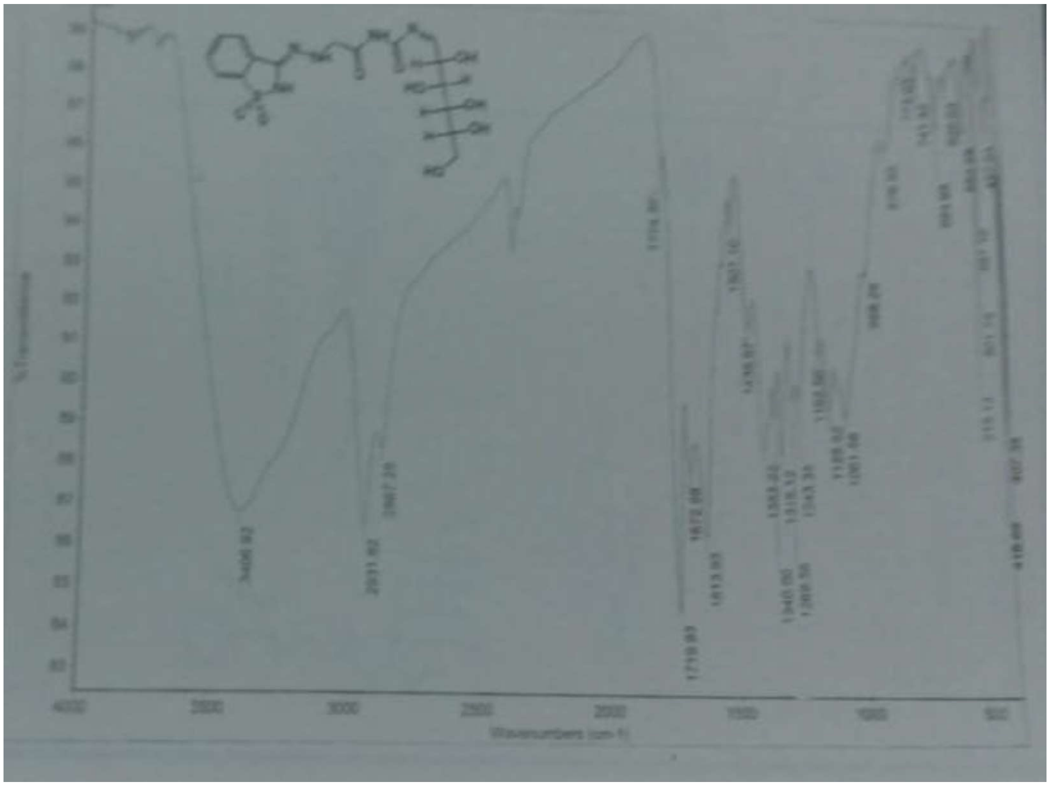

Compound 10a-IR

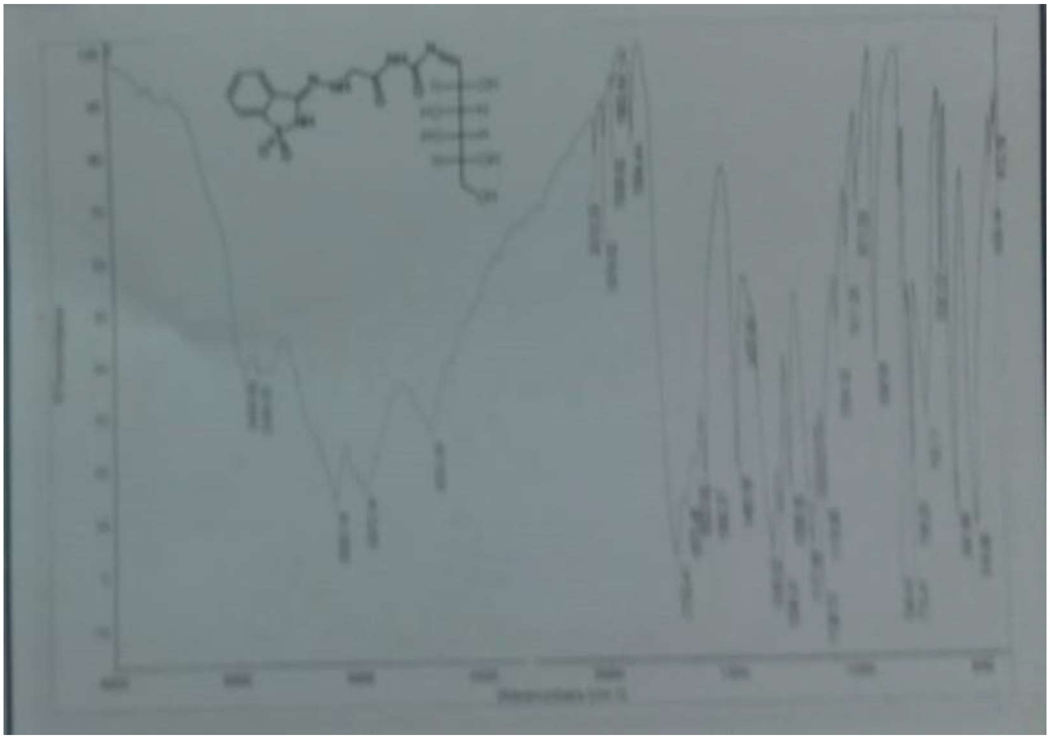

Compound 10b-IR

Compound 1

BRUKER

Current Data Parameters
NAME Dr Rabea D6 H1
EXPNO 10
PROCNO 1

F2 - Acquisition Parameters
Date_ 20190422
Time 14.38
INSTRUM spect
PROBHD 5 mm PABBO BB/
PULPROG zg30
TD 65536
SOLVENT DMSO
NS 16
DS 2
SWH 8012.820 Hz
FIDRES 0.122266 Hz
AQ 4.0894465 sec
RG 201.61
DW 62.400 usec
DE 6.50 usec
TE 298.2 K
D1 1.00000000 sec
TD0 1

======== CHANNEL f1 ========
SFO1 400.1324710 MHz
NUC1 1H
P1 15.00 usec
PLW1 10.39999962 W

F2 - Processing parameters
SI 65536
SF 400.1300066 MHz
WDW EM
SSB 0
LB 0.30 Hz
GB 0
PC 1.00

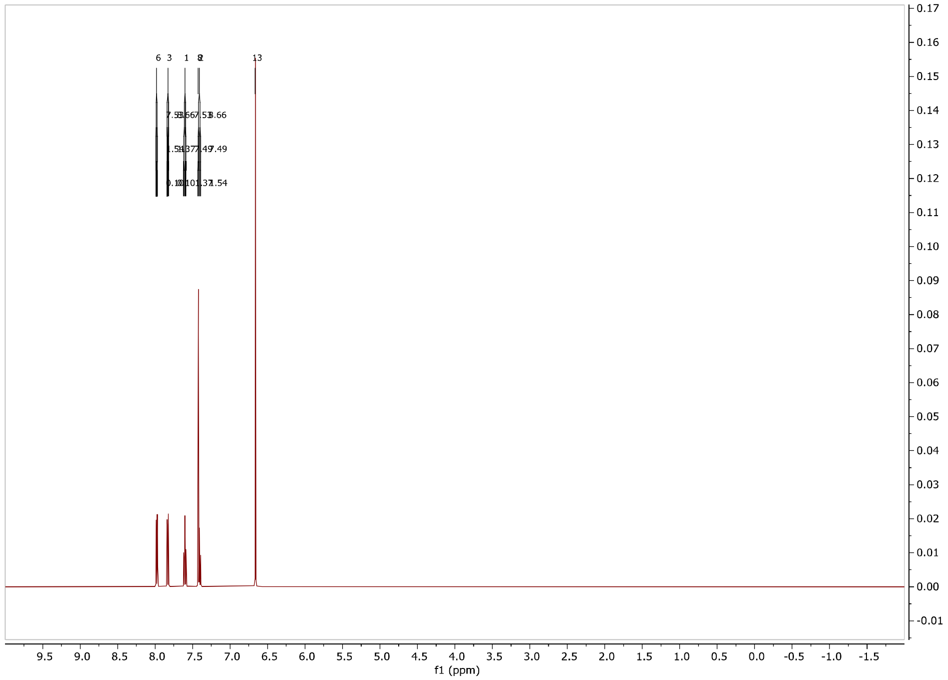

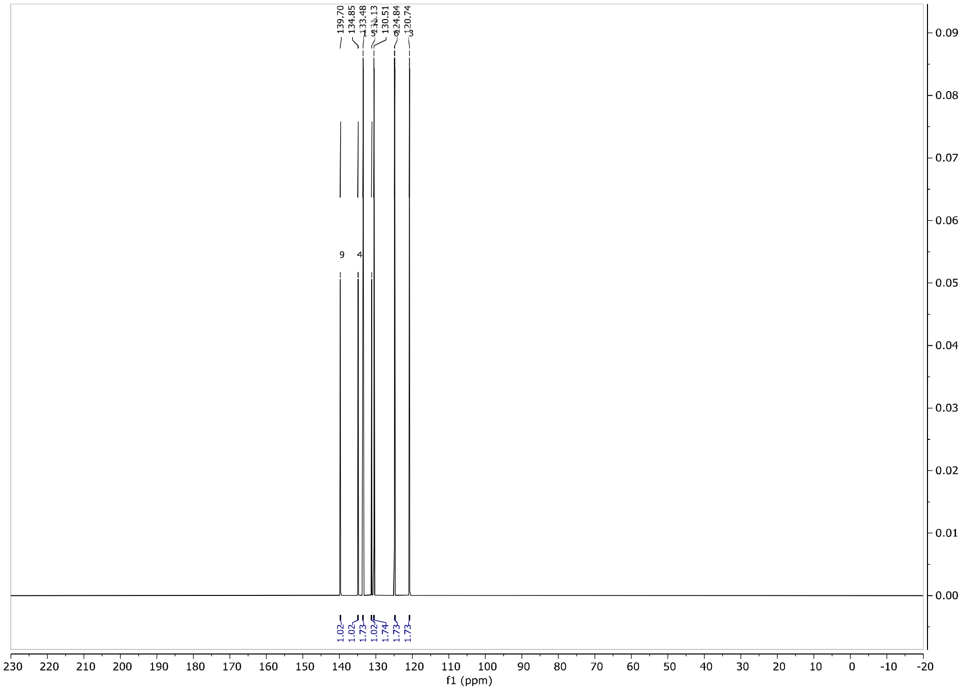

Compound 2

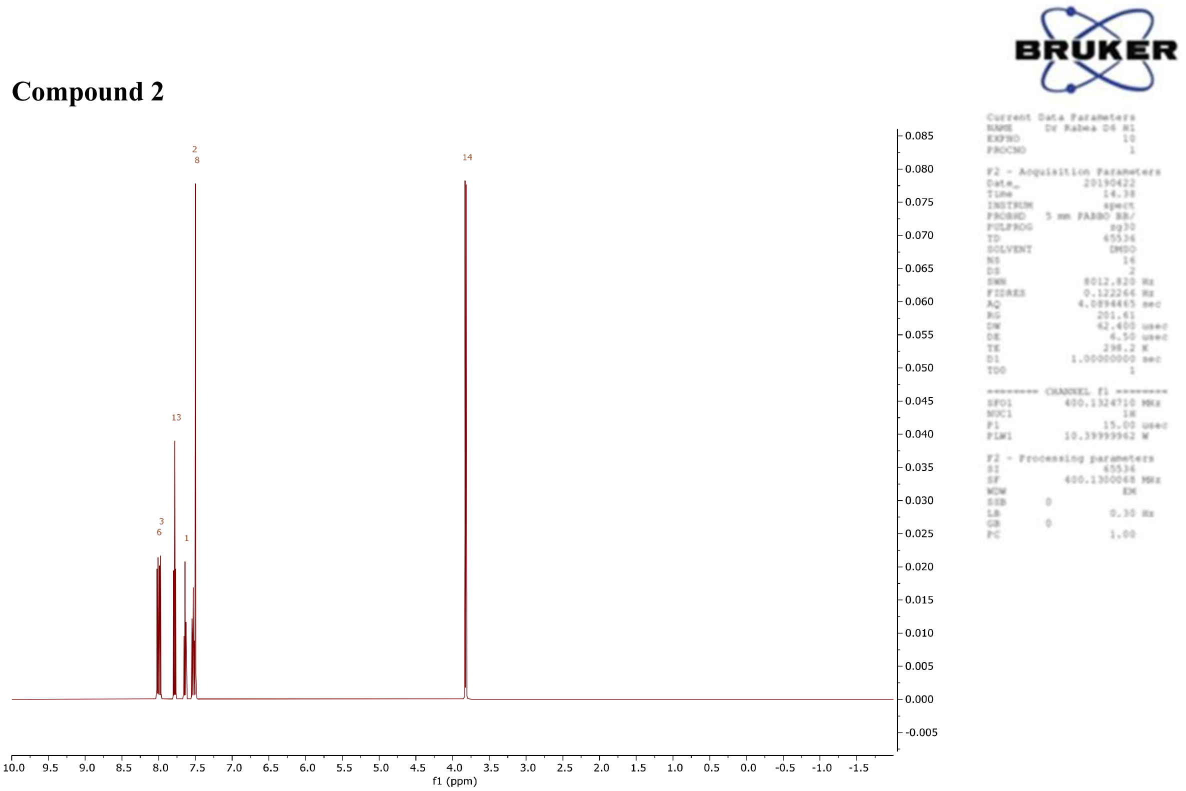

BRUKER
Current Data Parameters
NAME Dr Rabea D6 H1
EXPNO 10
PROCNO 1
F2 - Acquisition Parameters
Date_ 20190422
Time 14.38
INSTRUM spect
PROBHD 5 mm PABBO BB/
PULPROG zg30
TD 65536
SOLVENT DMSO
NS 16
DS 2
SWH 8012.820 Hz
FIDRES 0.122266 Hz
AQ 4.0894465 sec
RG 201.61
DW 62.400 usec
DE 6.50 usec
TE 298.2 K
D1 1.00000000 sec
TD0 1
======== CHANNEL f1 ========
SFO1 400.1324710 MHz
NUC1 1H
P1 15.00 usec
PLW1 10.39999962 W
F2 - Processing parameters
SI 65536
SF 400.1300068 MHz
WDW EM
SSB 0
LB 0.30 Hz
GB 0
PC 1.00
2
8
14
13
3
6
1
f1 (ppm)

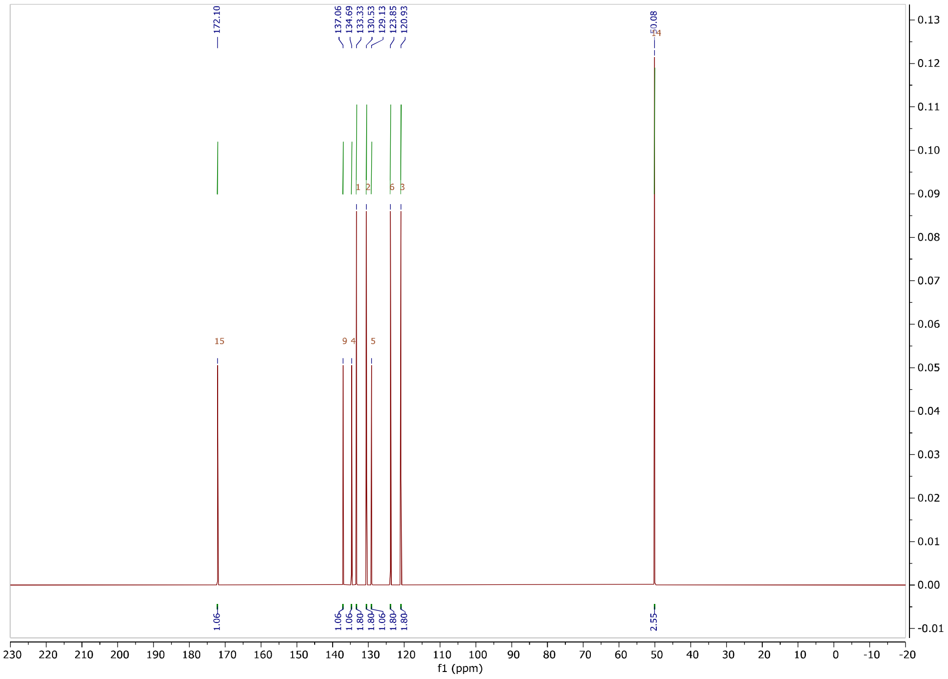

172.10
137.06
134.69
133.33
130.53
129.13
123.85
120.93
50.08
14
15
9 4
5
1 2
6 3
1.06
1.06
1.06
1.80
1.80
1.06
1.80
1.80
2.55
f1 (ppm)

Compound 4

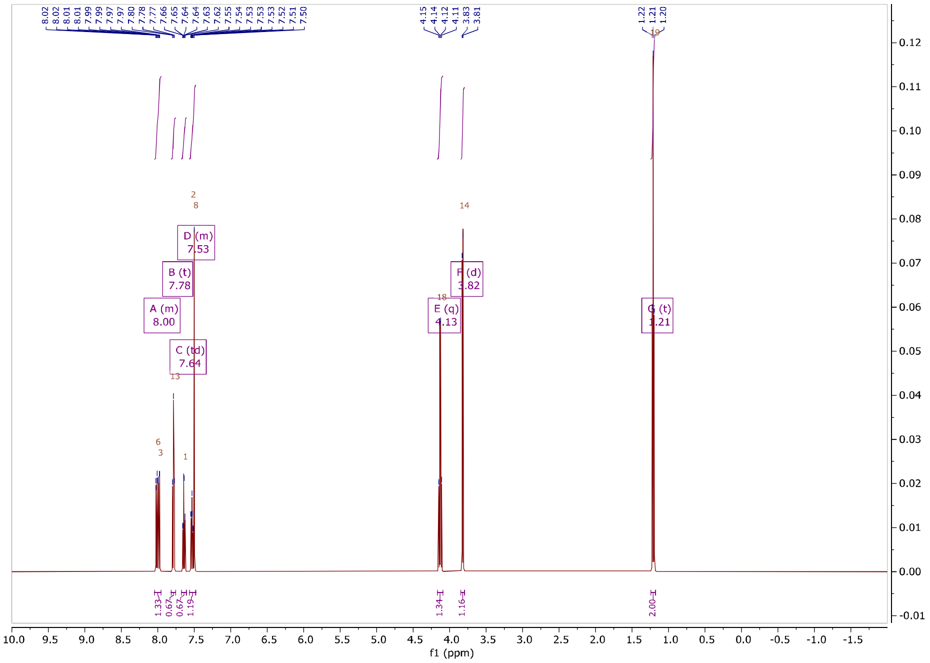

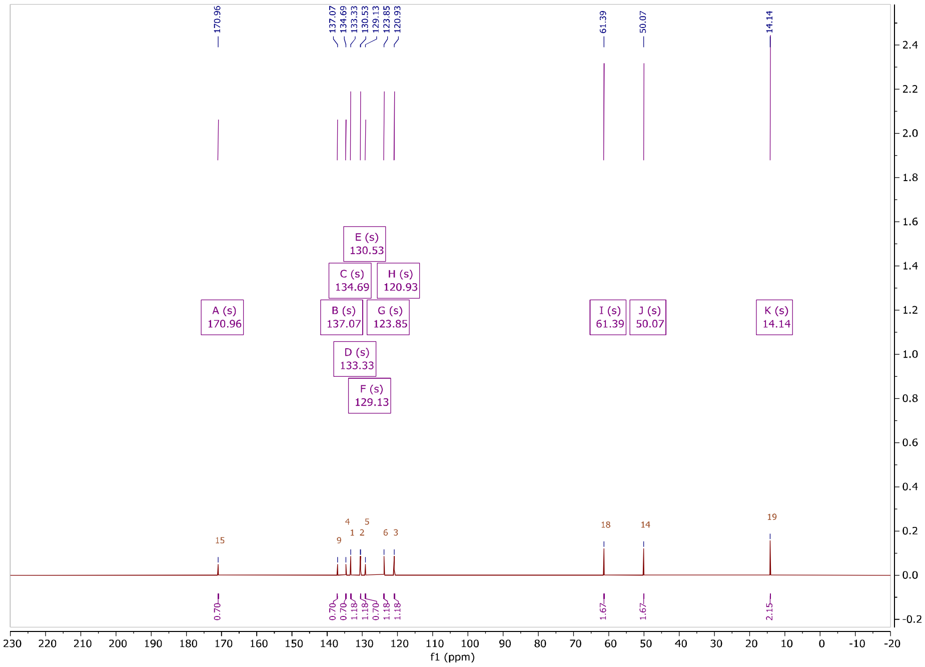


```
Current Data Parameters
NAME      Dr Rabea D6 H1
EXPNO                 10
PROCNO                 1

F2 - Acquisition Parameters
Date_           20190422
Time               14.38
INSTRUM            spect
PROBHD   5 mm PABBO BB/
PULPROG             zg30
TD                 65536
SOLVENT             DMSO
NS                    16
DS                     2
SWH            8012.820 Hz
FIDRES         0.122266 Hz
AQ            4.0894465 sec
RG                201.61
DW                62.400 usec
DE                  6.50 usec
TE                 298.2 K
D1            1.00000000 sec
TD0                    1

======== CHANNEL f1 ========
SFO1        400.1324710 MHz
NUC1                  1H
P1                 15.00 usec
PLW1         10.39999962 W

F2 - Processing parameters
SI                 65536
SF          400.1300048 MHz
WDW                   EM
SSB      0
LB                  0.30 Hz
GB       0
PC                  1.00
```

Compound 5

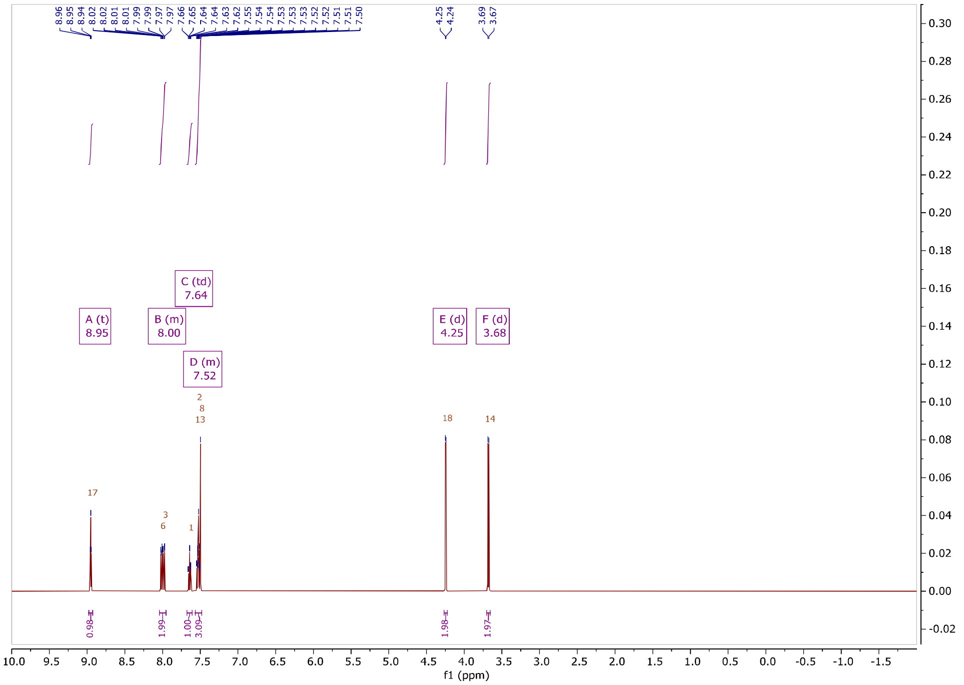


```
Current Data Parameters
NAME        Dr Rabea D6 H1
EXPNO                   10
PROCNO                   1

F2 - Acquisition Parameters
Date_             20190422
Time                 14.38
INSTRUM              spect
PROBHD     5 mm PABBO BB/
PULPROG               zg30
TD                   65536
SOLVENT               DMSO
NS                      16
DS                       2
SWH            8012.820 Hz
FIDRES         0.122266 Hz
AQ            4.0894465 sec
RG                  201.61
DW              62.400 usec
DE                4.50 usec
TE               298.2 K
D1           1.00000000 sec
TD0                      1

======== CHANNEL f1 ========
SFO1       400.1324710 MHz
NUC1                    1H
P1               15.00 usec
PLW1        10.39999962 W

F2 - Processing parameters
SI                   65536
SF         400.1300048 MHz
WDW                     EM
SSB      0
LB                0.30 Hz
GB       0
PC                    1.00
```

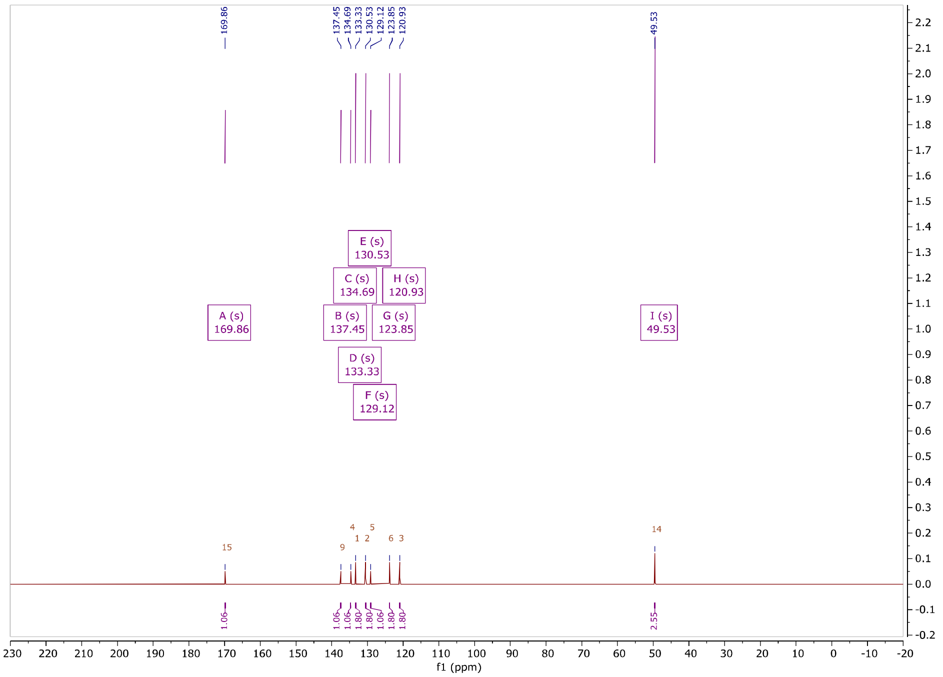

Compound 6a

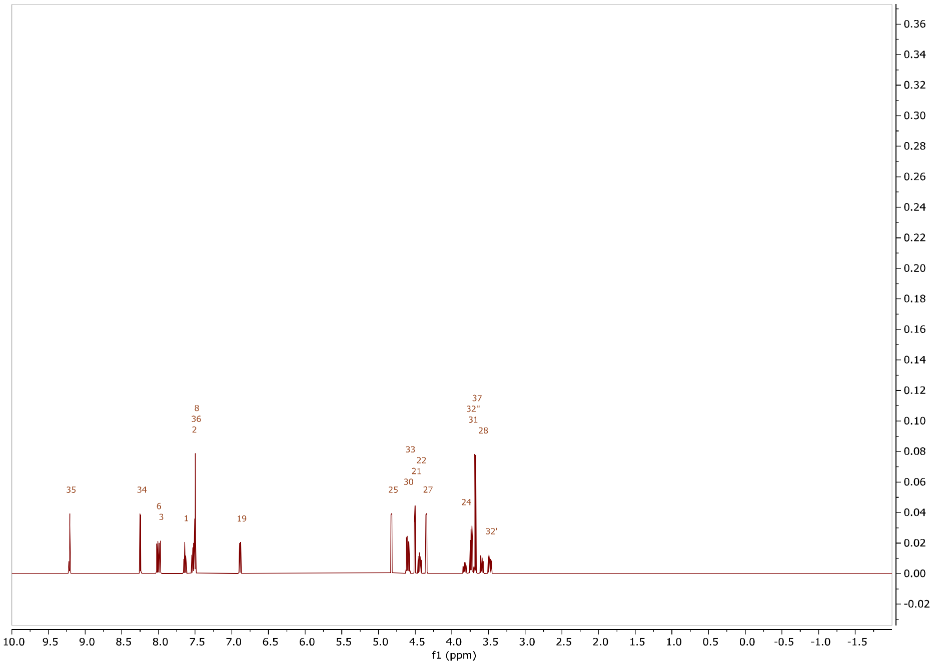

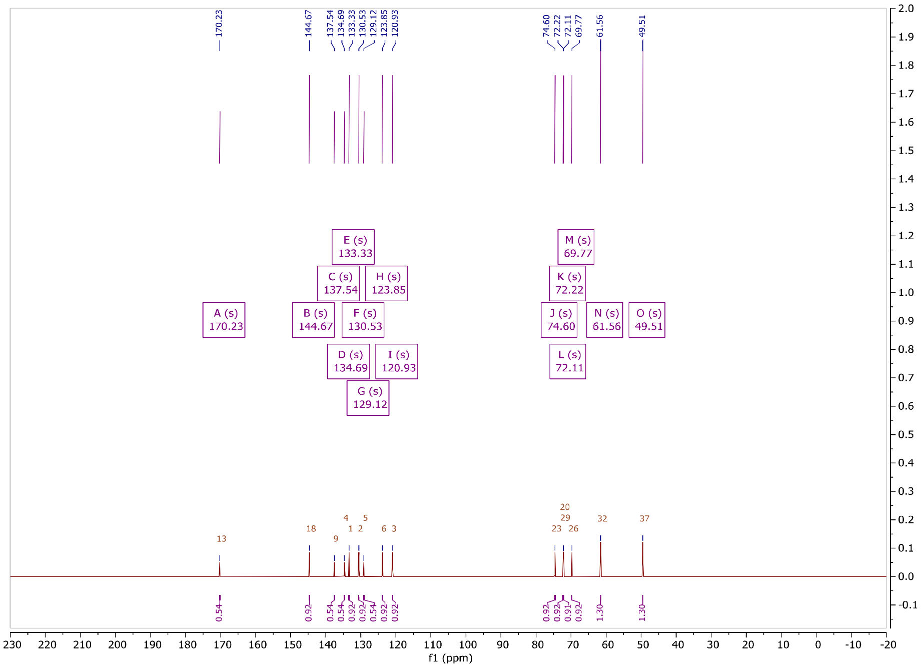

Current Data Parameters
NAME Dr Rabea D6 H1
EXPNO 10
PROCNO 1

F2 - Acquisition Parameters
Date_ 20190422
Time 14.38
INSTRUM spect
PROBHD 5 mm PABBO BB/
PULPROG zg30
TD 65536
SOLVENT DMSO
NS 16
DS 2
SWH 8012.820 Hz
FIDRES 0.122266 Hz
AQ 4.0894465 sec
RG 201.61
DW 62.400 usec
DE 6.50 usec
TE 298.2 K
D1 1.00000000 sec
TD0 1

======== CHANNEL f1 ========
SFO1 400.1324710 MHz
NUC1 1H
P1 15.00 usec
PLW1 10.39999962 W

F2 - Processing parameters
SI 65536
SF 400.1300048 MHz
WDW EM
SSB 0
LB 0.30 Hz
GB 0
PC 1.00

Compound 6b

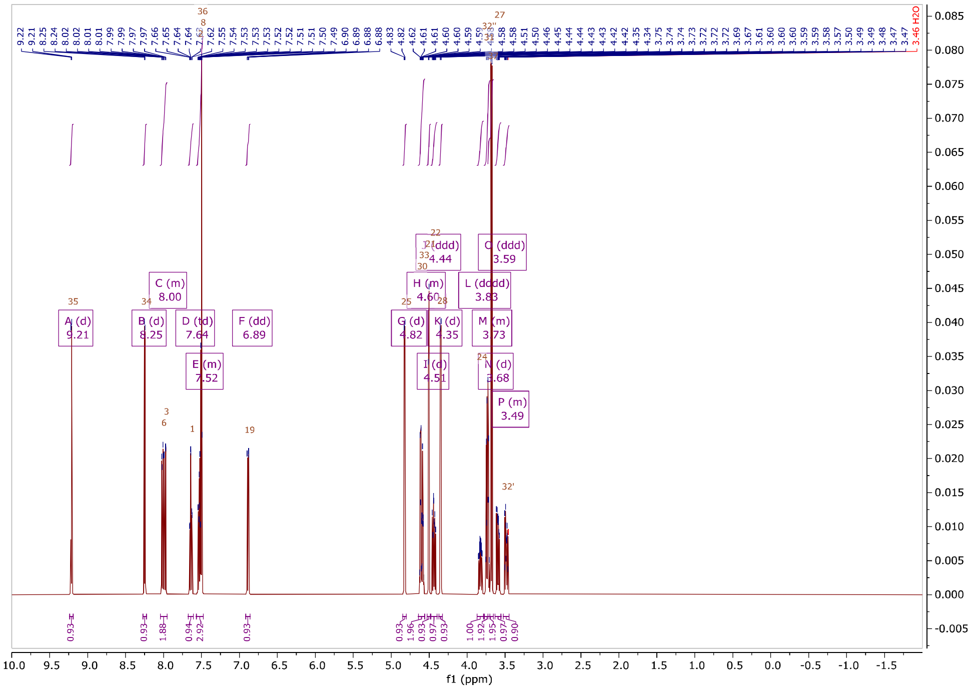

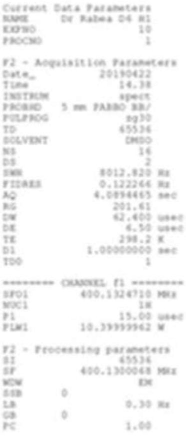

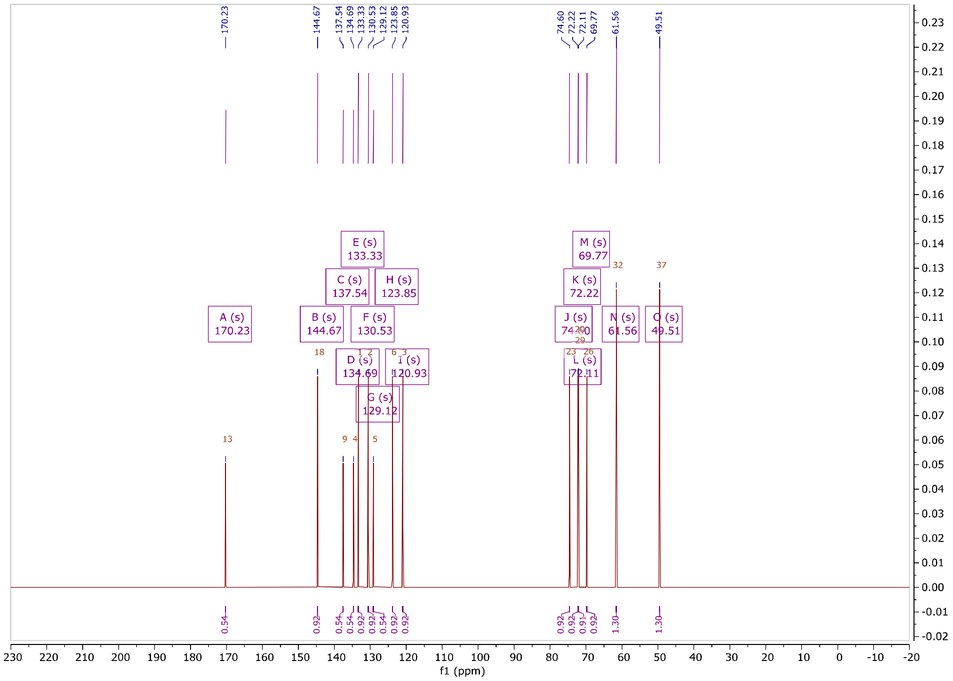

Compound 6c

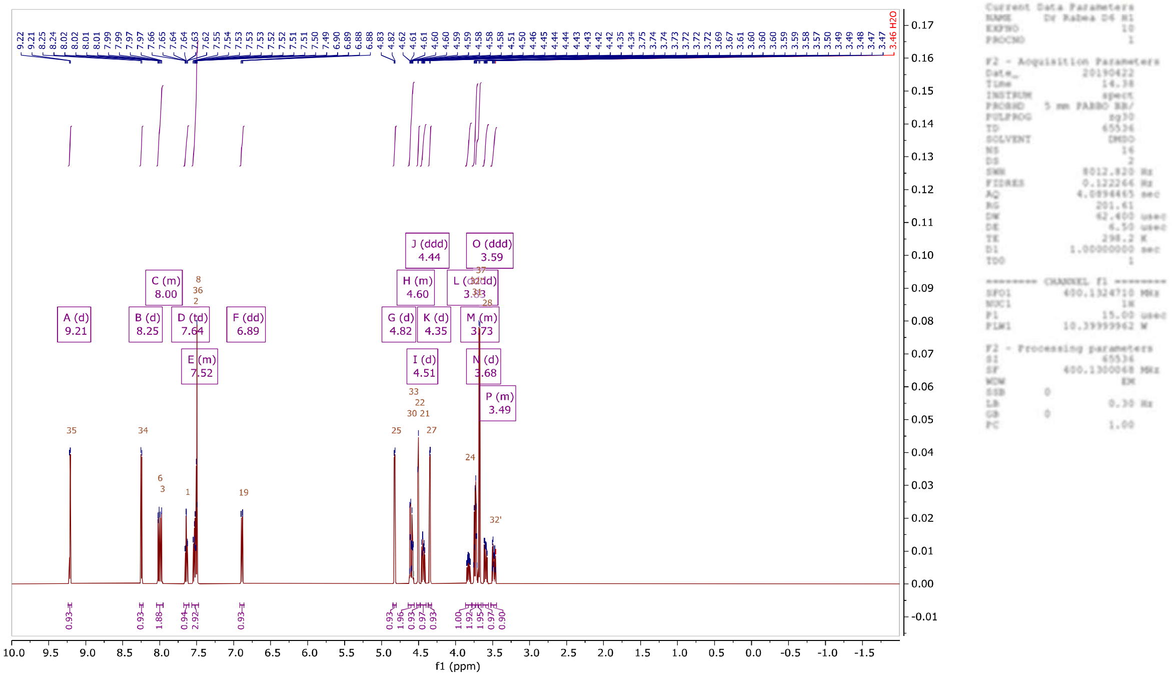
A (d) 9.21
B (d) 8.25
C (m) 8.00
D (td) 7.64
E (m) 7.52
F (dd) 6.89
G (d) 4.82
H (m) 4.60
I (d) 4.51
J (ddd) 4.44
K (d) 4.35
M (m) 3.73
N (d) 3.68
O (ddd) 3.59
P (m) 3.49
f1 (ppm)

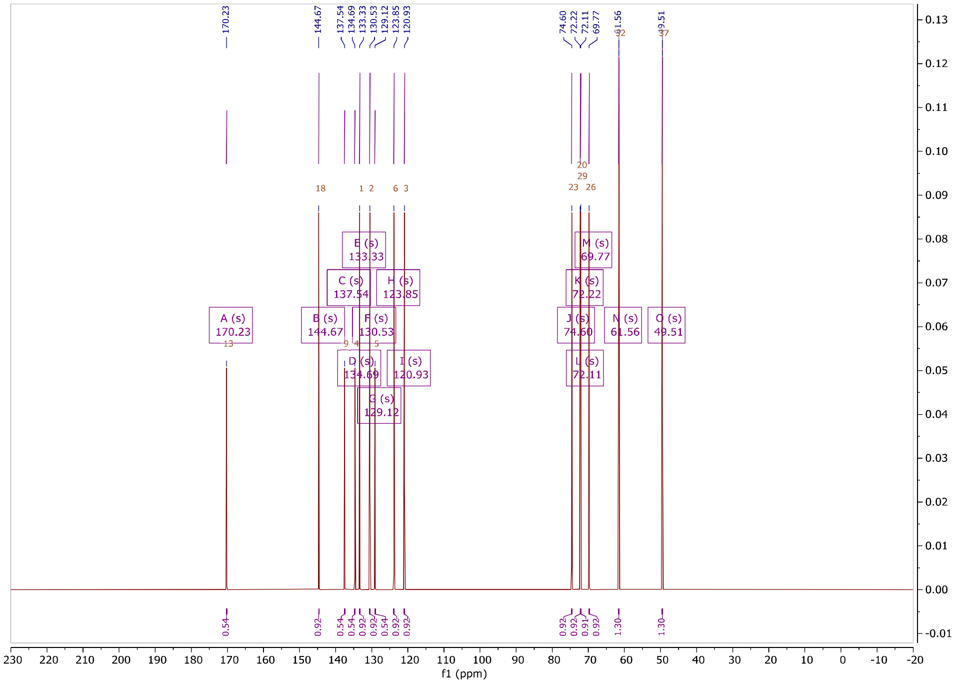
A (s) 170.23
B (s) 144.67
C (s) 137.54
D (s) 134.69
E (s) 133.33
F (s) 130.53
G (s) 129.12
H (s) 123.85
I (s) 120.93
J (s) 74.60
K (s) 72.22
L (s) 72.11
M (s) 69.77
N (s) 61.56
O (s) 49.51
f1 (ppm)

Compound 6d

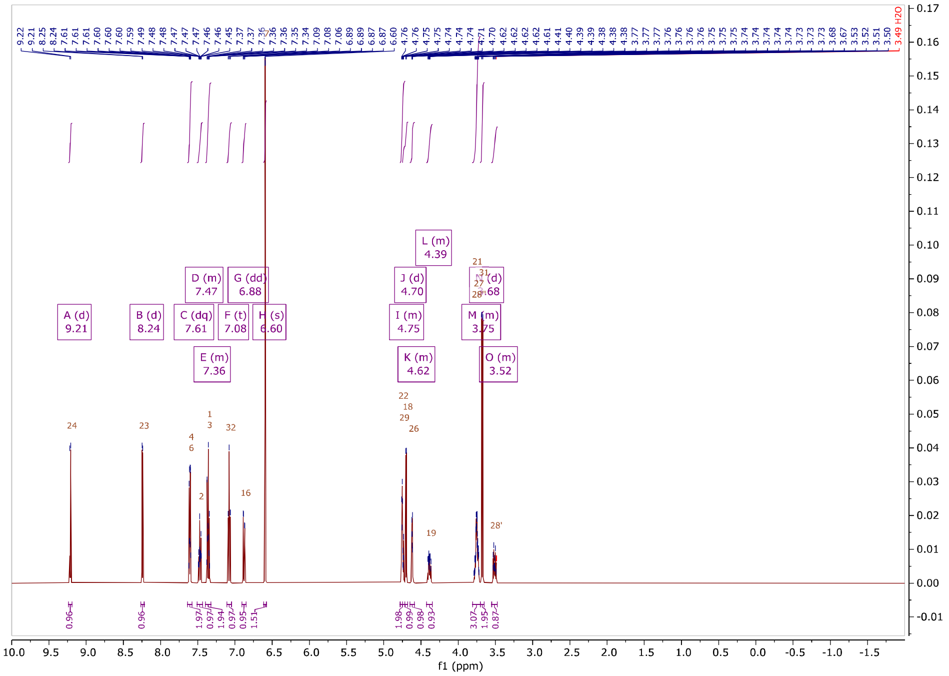


```
Current Data Parameters
NAME      Dr Rabea D6 H1
EXPNO               10
PROCNO               1

F2 - Acquisition Parameters
Date_         20190422
Time             14.38
INSTRUM          spect
PROBHD   5 mm PABBO BB/
PULPROG           zg30
TD               65536
SOLVENT           DMSO
NS                  16
DS                   2
SWH           8012.820 Hz
FIDRES        0.122266 Hz
AQ           4.0894465 sec
RG              201.61
DW              62.400 usec
DE                6.50 usec
TE               298.2 K
D1          1.00000000 sec
TD0                  1

======== CHANNEL f1 ========
SFO1       400.1324710 MHz
NUC1                1H
P1               15.00 usec
PLW1       10.39999962 W

F2 - Processing parameters
SI               65536
SF         400.1300048 MHz
WDW                 EM
SSB      0
LB                0.30 Hz
GB       0
PC                1.00
```

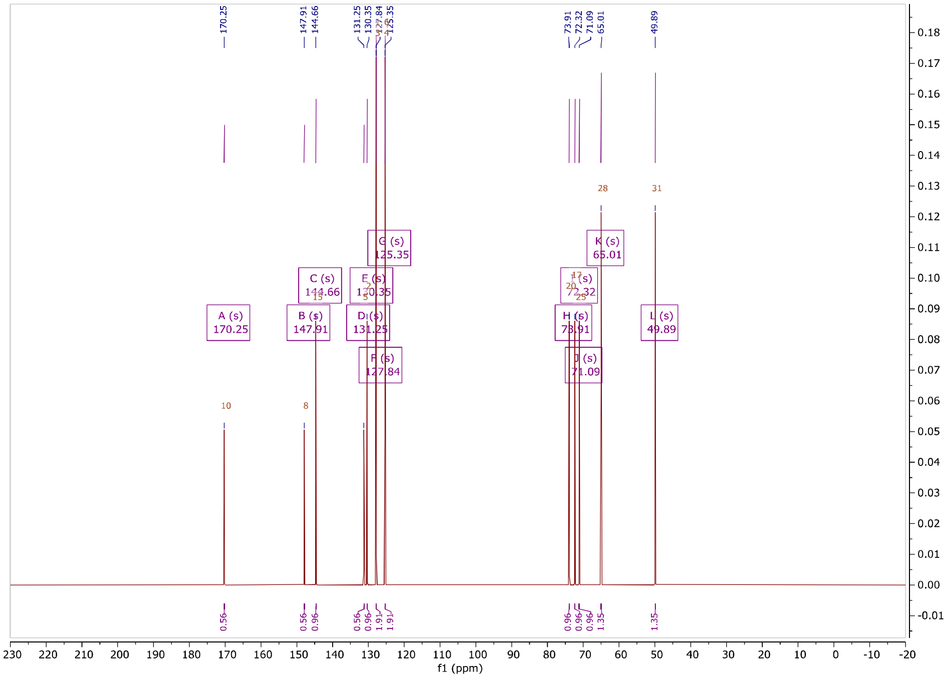

Compound 7a

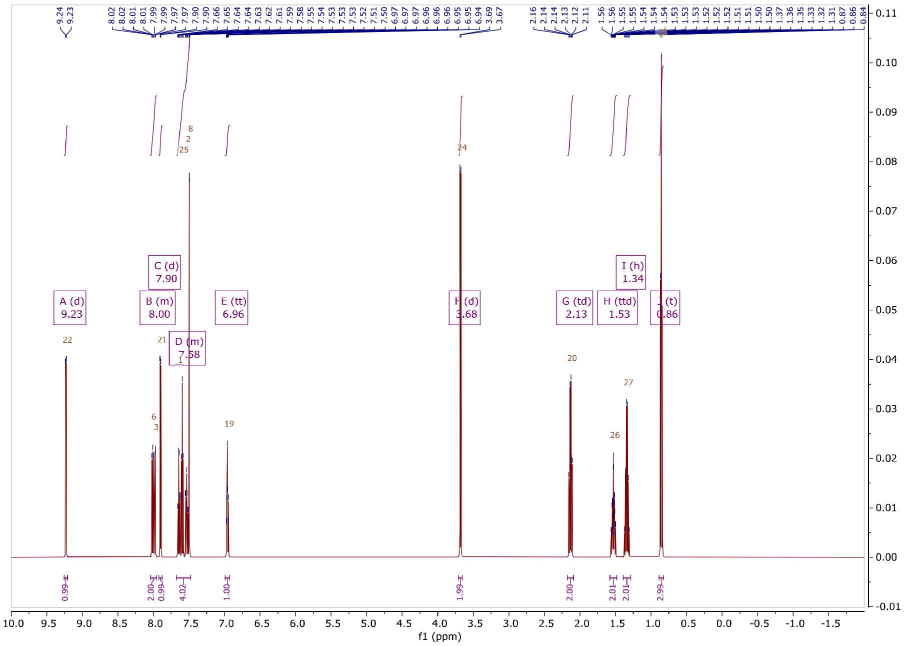

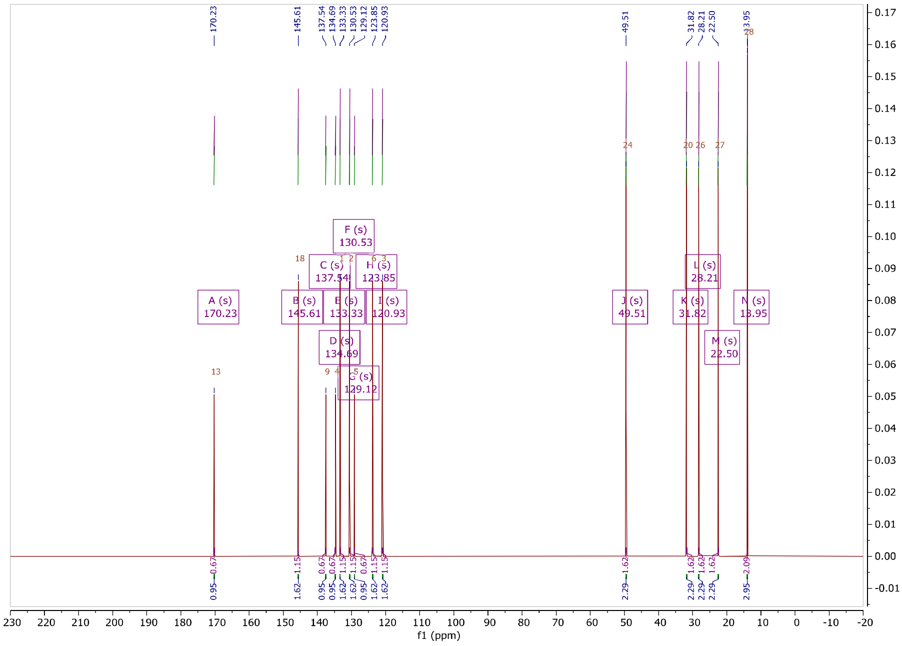

Current Data Parameters
NAME Dr Rabea D6 H1
EXPNO 10
PROCNO 1

F2 - Acquisition Parameters
Date_ 20190422
Time 14.38
INSTRUM spect
PROBHD 5 mm PABBO BB/
PULPROG zg30
TD 65536
SOLVENT DMSO
NS 16
DS 2
SWH 8012.820 Hz
FIDRES 0.122266 Hz
AQ 4.0894465 sec
RG 201.61
DW 62.400 usec
DE 6.50 usec
TE 298.2 K
D1 1.00000000 sec
TD0 1

======== CHANNEL f1 ========
SFO1 400.1324710 MHz
NUC1 1H
P1 15.00 usec
PLW1 10.39999962 W

F2 - Processing parameters
SI 65536
SF 400.1300048 MHz
WDW EM
SSB 0
LB 0.30 Hz
GB 0
PC 1.00

Compound 7b

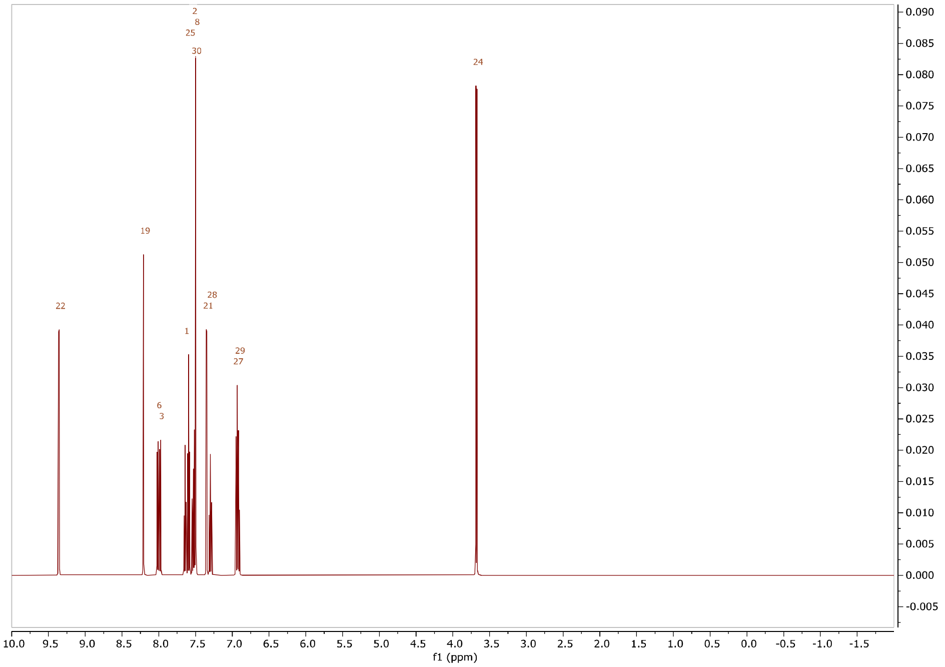

Current Data Parameters
NAME Dr Rabea D6 H1
EXPNO 10
PROCNO 1

F2 - Acquisition Parameters
Date_ 20190422
Time 14.38
INSTRUM spect
PROBHD 5 mm PABBO BB/
PULPROG zg30
TD 65536
SOLVENT DMSO
NS 16
DS 2
SWH 8012.820 Hz
FIDRES 0.122266 Hz
AQ 4.0894465 sec
RG 201.61
DW 62.400 usec
DE 6.50 usec
TE 298.2 K
D1 1.00000000 sec
TD0 1

======== CHANNEL f1 ========
SFO1 400.1324710 MHz
NUC1 1H
P1 15.00 usec
PLW1 10.39999962 W

F2 - Processing parameters
SI 65536
SF 400.1300048 MHz
WDW EM
SSB 0
LB 0.30 Hz
GB 0
PC 1.00

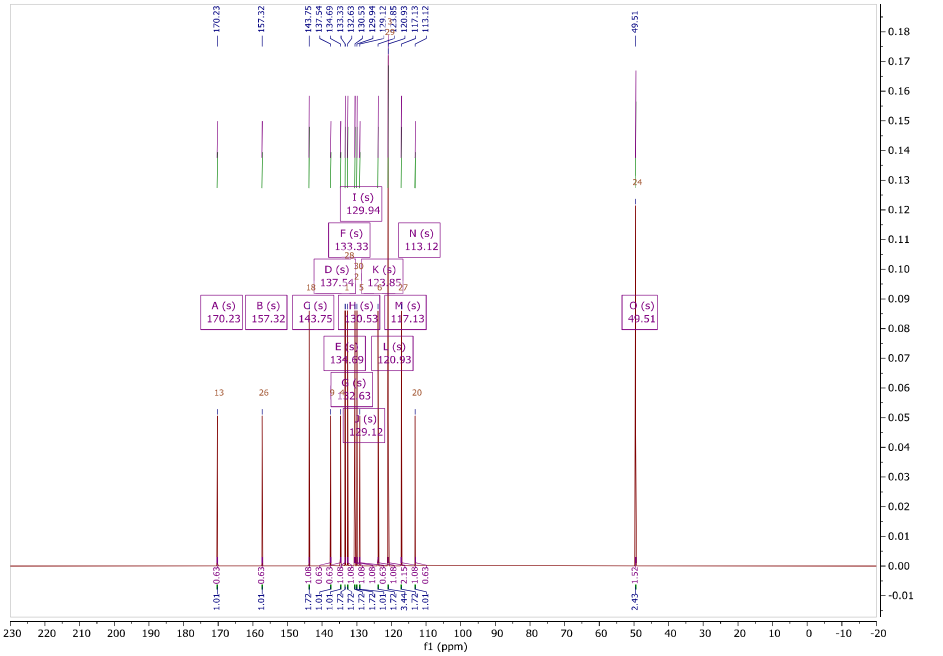

Compound 8a

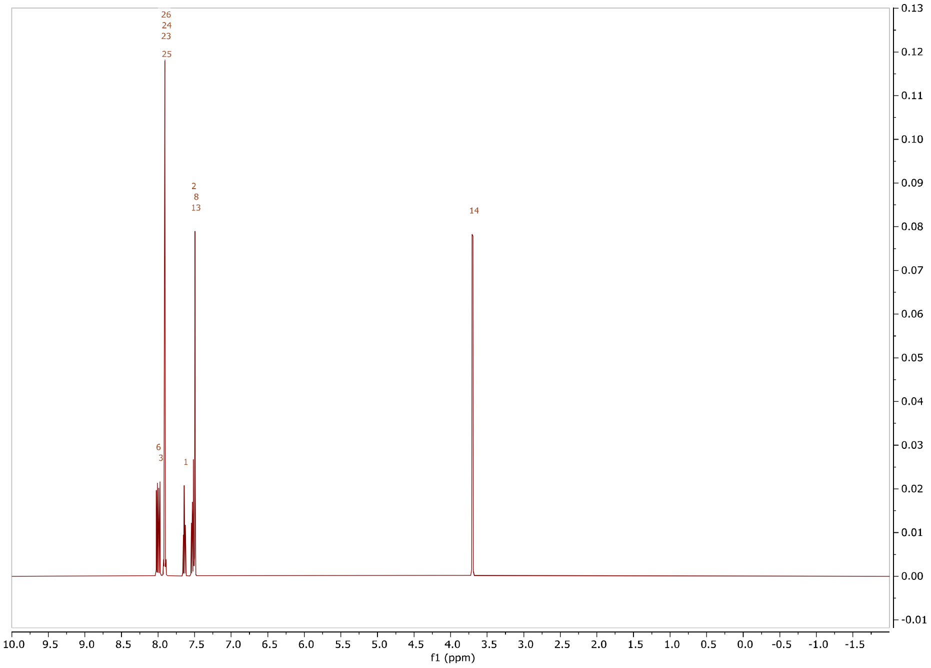

Current Data Parameters	
NAME	Dr Rabea D6 H1
EXPNO	10
PROCNO	1
F2 - Acquisition Parameters	
Date_	20190422
Time	14.38
INSTRUM	spect
PROBHD	5 mm PABBO BB/
PULPROG	zg30
TD	65536
SOLVENT	DMSO
NS	16
DS	2
SWH	8012.820 Hz
FIDRES	0.122266 Hz
AQ	4.0894465 sec
RG	201.61
DW	62.400 usec
DE	6.50 usec
TE	298.2 K
D1	1.00000000 sec
TD0	1
======== CHANNEL f1 ========	
SFO1	400.1324710 MHz
NUC1	1H
P1	15.00 usec
PLW1	10.39999962 W
F2 - Processing parameters	
SI	65536
SF	400.1300048 MHz
WDW	EM
SSB	0
LB	0.30 Hz
GB	0
PC	1.00

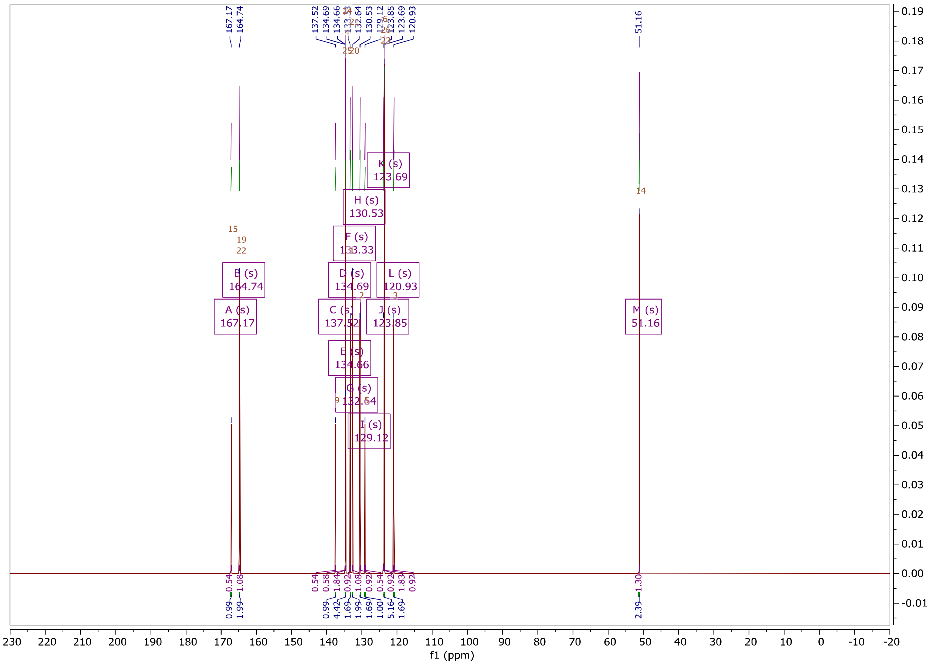

Compound 8b

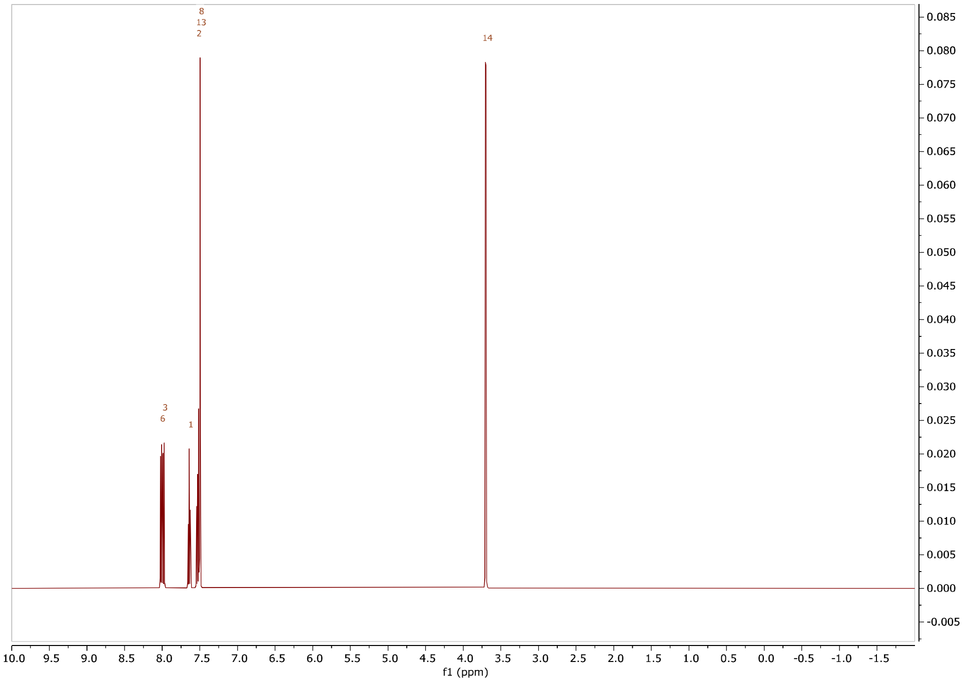

Current Data Parameters		
NAME	Dr Rabea D6 H1	
EXPNO	10	
PROCNO	1	
F2 - Acquisition Parameters		
Date_	20190422	
Time	14.38	
INSTRUM	spect	
PROBHD	5 mm PABBO BB/	
PULPROG	zg30	
TD	65536	
SOLVENT	DMSO	
NS	16	
DS	2	
SWH	8012.820	Hz
FIDRES	0.122266	Hz
AQ	4.0894465	sec
RG	201.61	
DW	62.400	usec
DE	6.50	usec
TE	298.2	K
D1	1.00000000	sec
TD0	1	
======== CHANNEL f1 ========		
SFO1	400.1324710	MHz
NUC1	1H	
P1	15.00	usec
PLW1	10.39999962	W
F2 - Processing parameters		
SI	65536	
SF	400.1300068	MHz
WDW	EM	
SSB	0	
LB	0.30	Hz
GB	0	
PC	1.00	

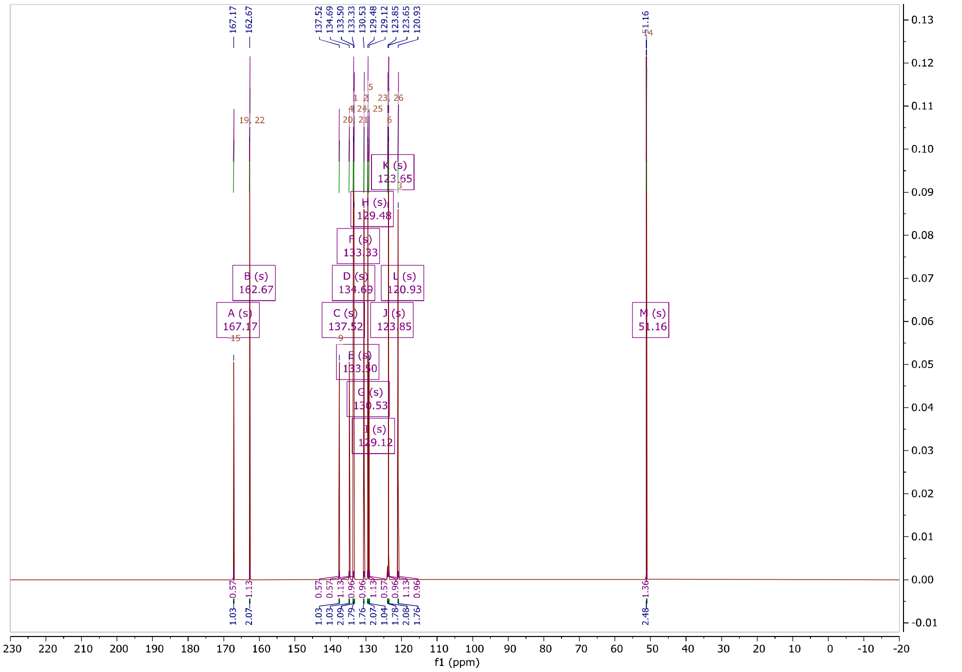

Compound 9

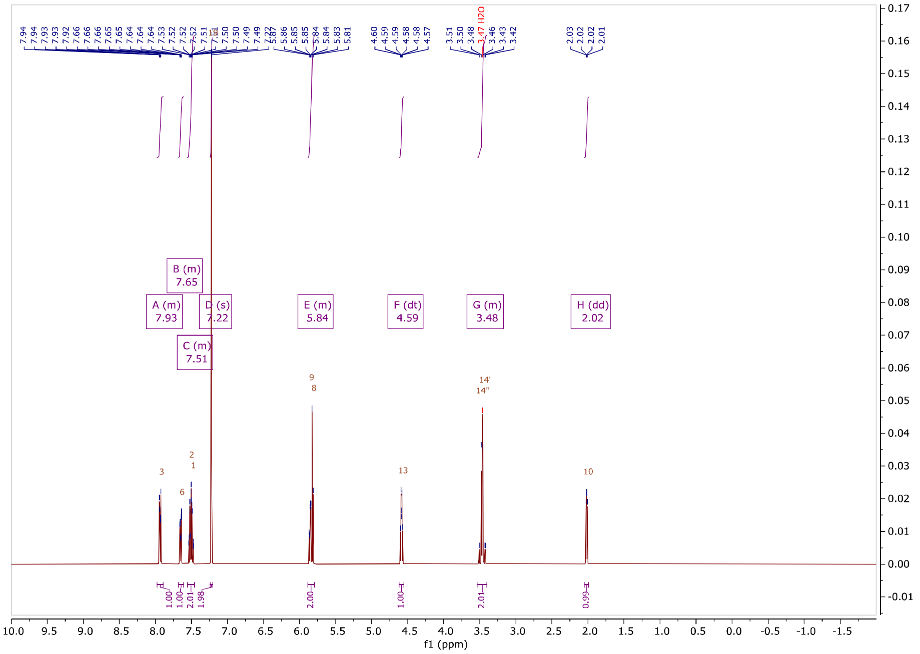
A (m) 7.93
B (m) 7.65
C (m) 7.51
D (s) 7.22
E (m) 5.84
F (dt) 4.59
G (m) 3.48
H (dd) 2.02
3.47 H2O
f1 (ppm)

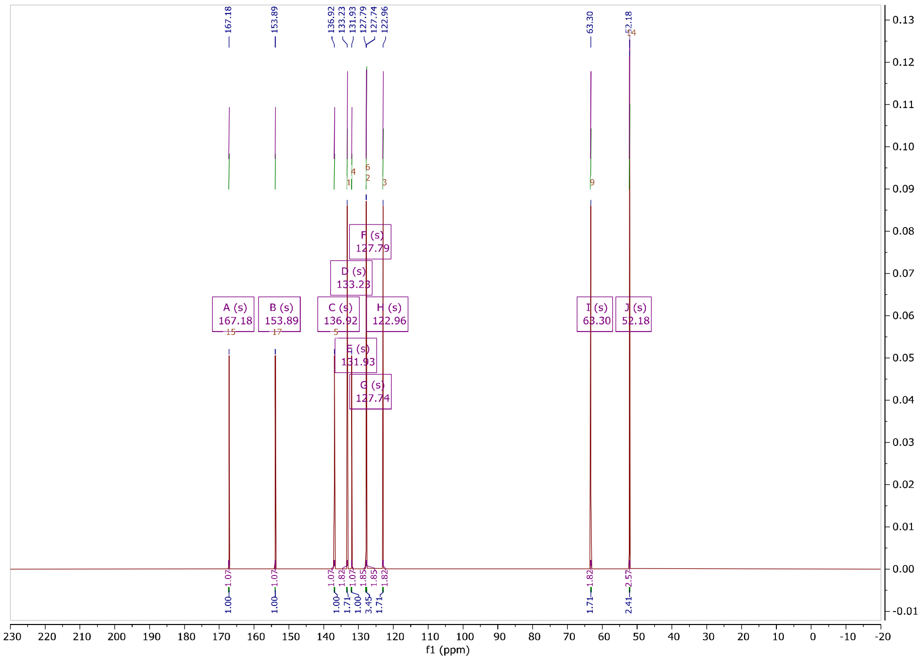
167.18
153.89
136.92
133.23
131.93
127.79
127.74
122.96
63.30
52.18
A (s) 167.18
B (s) 153.89
C (s) 136.92
D (s) 133.23
E (s) 131.93
F (s) 127.79
G (s) 127.74
H (s) 122.96
I (s) 63.30
J (s) 52.18
f1 (ppm)

Compound 10a

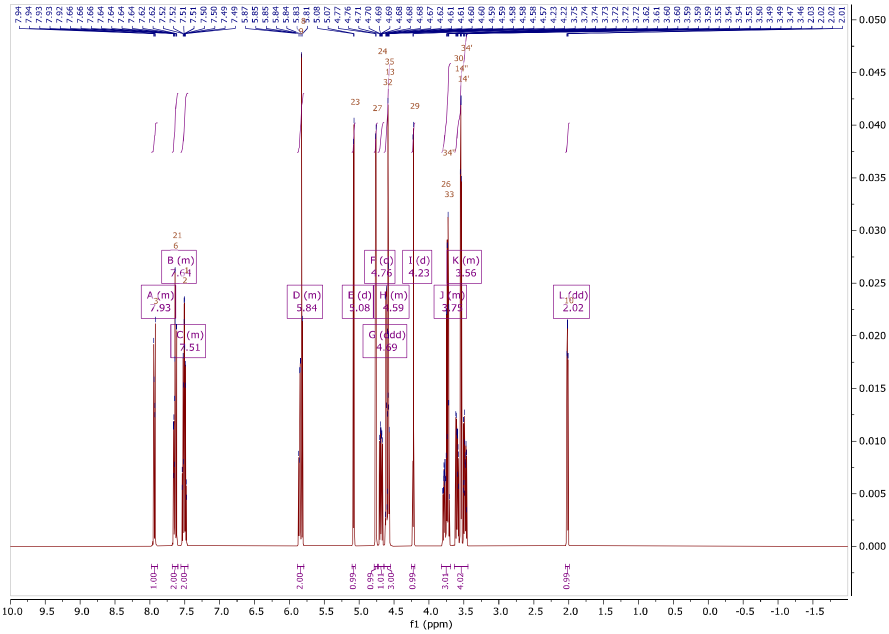

Current Data Parameters	
NAME	Dr Rabea D6 H1
EXPNO	10
PROCNO	1
F2 - Acquisition Parameters	
Date_	20190422
Time	14.38
INSTRUM	spect
PROBHD	5 mm PABBO BB/
PULPROG	zg30
TD	65536
SOLVENT	DMSO
NS	16
DS	2
SWH	8012.820 Hz
FIDRES	0.122266 Hz
AQ	4.0894465 sec
RG	201.61
DW	62.400 usec
DE	6.50 usec
TE	298.2 K
D1	1.00000000 sec
TD0	1
======== CHANNEL f1 ========	
SFO1	400.1324710 MHz
NUC1	1H
P1	15.00 usec
PLW1	10.39999962 W
F2 - Processing parameters	
SI	65536
SF	400.1300068 MHz
WDW	EM
SSB	0
LB	0.30 Hz
GB	0
PC	1.00

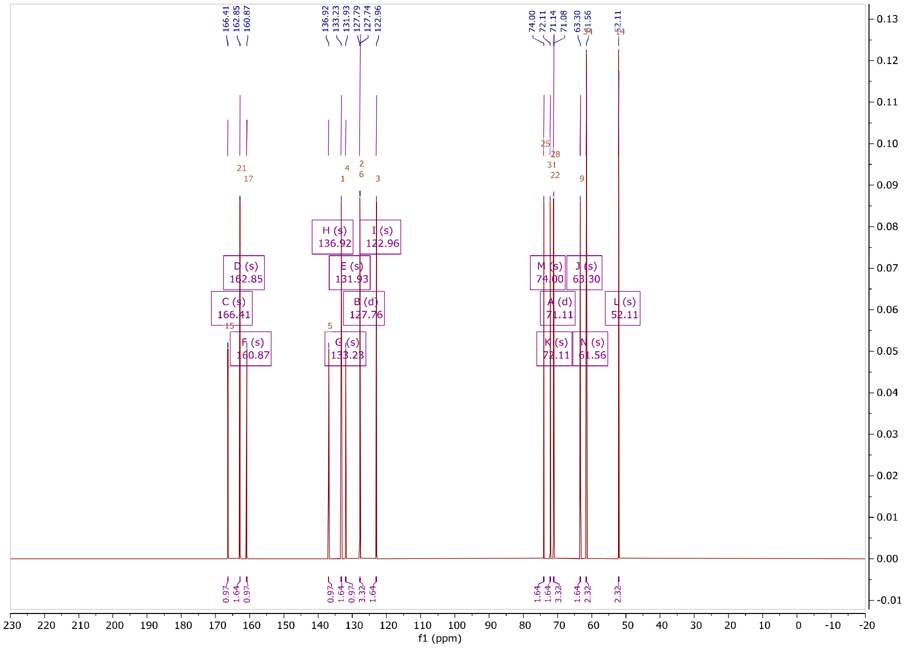

Compound 10b

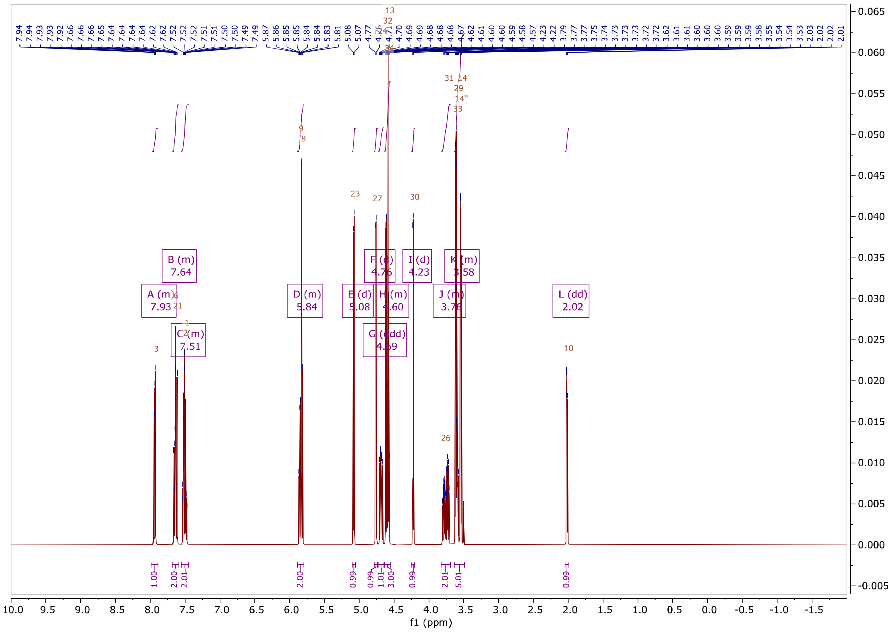

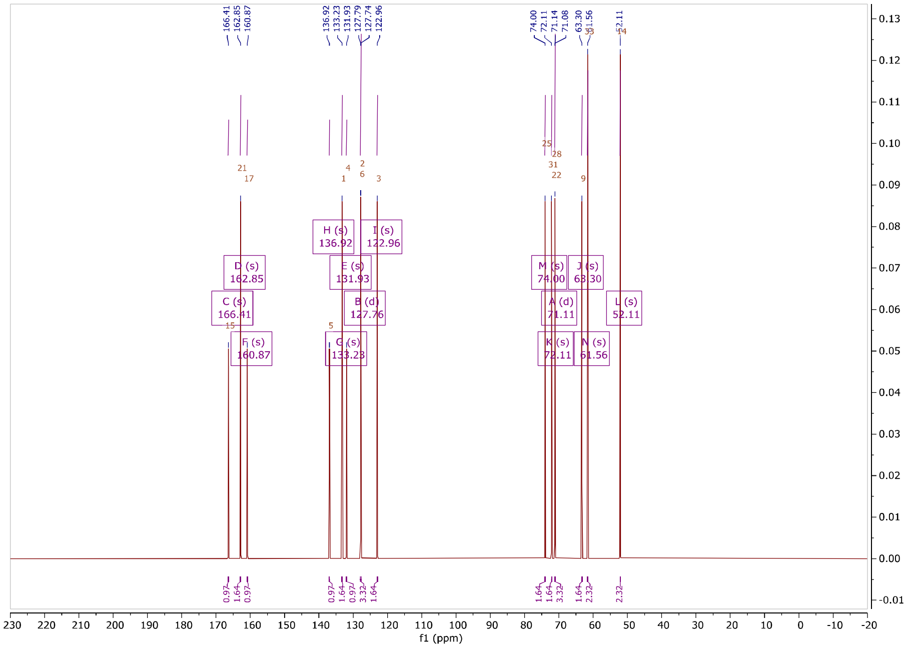

BRUKER

Current Data Parameters
NAME Dr Rabea D6 H1
EXPNO 10
PROCNO 1

F2 - Acquisition Parameters
Date_ 20190422
Time 14.38
INSTRUM spect
PROBHD 5 mm PABBO BB/
PULPROG zg30
TD 65536
SOLVENT DMSO
NS 16
DS 2
SWH 8012.820 Hz
FIDRES 0.122266 Hz
AQ 4.0894465 sec
RG 201.61
DW 62.400 usec
DE 6.50 usec
TE 298.2 K
D1 1.00000000 sec
TD0 1

======== CHANNEL f1 ========
SFO1 400.1324710 MHz
NUC1 1H
P1 15.00 usec
PLW1 10.39999962 W

F2 - Processing parameters
SI 65536
SF 400.1300048 MHz
WDW EM
SSB 0
LB 0.30 Hz
GB 0
PC 1.00
